# New classes of potent heparanase inhibitors from ligand-based virtual screening

**DOI:** 10.1080/14756366.2020.1811701

**Published:** 2020-09-09

**Authors:** Daniele Pala, Laura Scalvini, Gian Marco Elisi, Alessio Lodola, Marco Mor, Gilberto Spadoni, Fabiana F. Ferrara, Emiliano Pavoni, Giuseppe Roscilli, Ferdinando M. Milazzo, Gianfranco Battistuzzi, Silvia Rivara, Giuseppe Giannini

**Affiliations:** a Dipartimento di Scienze degli Alimenti e del Farmaco, Università degli Studi di Parma, Parma, Italy; b Dipartimento di Scienze Biomolecolari, Università degli Studi di Urbino “Carlo Bo”, Urbino, Italy; c Takis s.r.l., Roma, Italy; d R&D Alfasigma S.p.A., Roma, Italy

**Keywords:** Heparanase, heparan sulphate, virtual screening

## Abstract

Heparanase is a validated target in cancer therapy and a potential target for several inflammatory pathologies. A ligand-based virtual screening of commercial libraries was performed to expand the chemical space of small-molecule inhibitors. The screening was based on similarity with known inhibitors and was performed in several runs, starting from literature compounds and progressing through newly discovered inhibitors. Among the fifty-five tested compounds, nineteen had IC_50_ values lower than 5 µM and some showed remarkable potencies. Importantly, tere- and isophthalamides derivatives belong to new structural classes of heparanase inhibitors and some of them showed enzyme affinities (**61** and **63**, IC_50_ = 0.32 and 0.12 µM, respectively) similar to those of the most potent small-molecule inhibitors reported so far. Docking studies provided a comprehensive binding hypothesis shared by compounds with significant structural diversity. The most potent inhibitors reduced cell invasiveness and inhibited the expression of proangiogenic factors in tumour cell lines.

## Introduction

Heparan sulphate (HS) is a glycosaminoglycan polysaccharide composed of repeating polysulfated disaccharide units of glucosamine and hexuronic (glucuronic or iduronic) acid residues^1^. HS is bound to core proteins in HS proteoglycans (HSPGs) which are widely expressed on cellular surfaces, in basement membranes and in the extracellular matrix, where they are involved in the maintenance of structural integrity and insolubility, and in tissue organisation. HSPGs regulate cellular homeostatic processes given the ability of HS to bind several bioactive extracellular components, comprising growth factors, chemokines, enzymes, lipoproteins and coagulation factors, and to affect their bioavailability and activity^2^.

The *endo*-β-D-glucuronidase heparanase (EC 3.2.1.166) is responsible for the cleavage of HS. Heparanase is present in the intracellular compartment and in the extracellular space, where hydrolysis of HSPG side chains leads to the release of bioactive components and oligosaccharide cofactors required for cellular signalling (e.g. allowing the formation of fibroblast growth factor FGF:HS:FGF receptor heterotrimers). Heparanase also exerts non-enzymatic activities which contribute to its complex role in both physiological and pathological conditions^3^. During adult life, heparanase is present in few tissues, with high levels only detected in some blood-borne cells. On the other hand, heparanase is overexpressed in several pathological conditions where alteration of HS affects tissue structure and integrity, cell adhesion and migration^4^^,^^5^. In particular, heparanase overexpression has been correlated with tumour survival and progression, angiogenesis, cell dissemination and metastasis, and with poor prognosis. Moreover, treatment with classical cytotoxic agents often induces heparanase expression, which concurs to the development of drug resistance^6^. Heparanase is highly active also in non-malignant pathologies, such as in a variety of inflammatory conditions in which HS degradation and the consequent extracellular matrix remodelling facilitate recruitment and migration of leukocytes and activation of innate immune cells^7^^,^^8^.

**Figure 1.**
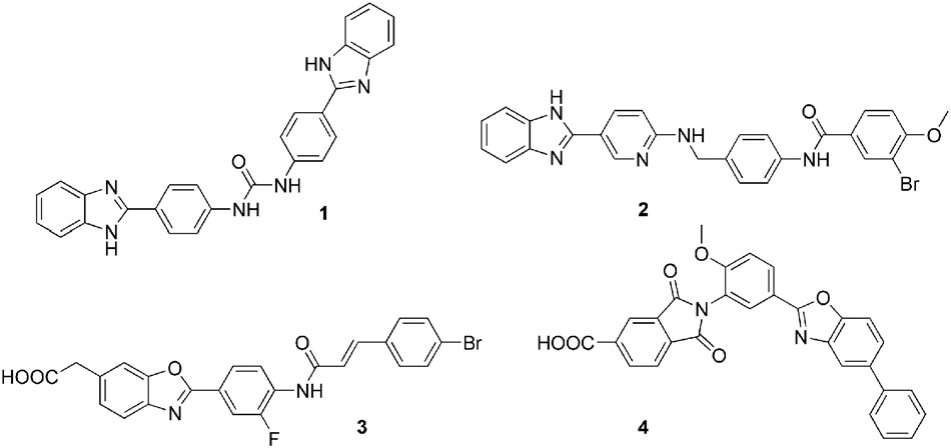
Small-molecule heparanase inhibitors evaluated in preclinical studies.

Pharmacological inhibition of heparanase enzymatic activity has been achieved through several approaches, comprising nucleic acid-based inhibitors, monoclonal antibodies and sulphated polysaccharides and oligosaccharides^4^^,^^9^. However, despite the established negative role of heparanase in several pathologies, development programmes of heparanase inhibitors have brought few inhibitors to clinical trials. They are synthetic or semi-synthetic oligo- and polysaccharides, mainly evaluated in cancer therapy. Muparfostat (PI-88) is a mixture of highly sulphated monophosphorylated mannose oligosaccharides^10^; pixatimod (PG545), a synthetic HS mimetic in which the oligosaccharide portion is fused with a cholestanol moiety^11^; roneparstat (SST0001) is a semisynthetic non-anticoagulant 100% N-acetylated and glycol-split heparin derivative^12^; necuparanib (M-402) is another glycol-split HS mimetic similar to roneparstat^13^. Overall, these compounds were well tolerated, with limited or manageable side effects and with encouraging signals of anticancer activity. However, they suffer from some limitations related to their origin and nature. In fact, muparfostat, and heparin derivatives roneparstat and necuparanib are heterogeneous mixtures which could limit product characterisation and standardisation, and all these clinical candidates need to be administered by a parenteral route. Small-molecule heparanase inhibitors could be, in principle, more manageable and suitable for oral administration, and some of them have been prepared and evaluated at preclinical stages,^4^^,^^9^^,^^14^ as the 1,3-diphenylurea **1**^15^, the 2-aryl-benzimidazole **2**^16^, the benzoxazol-5-yl-acetic acid **3**^17^ and the acidic phthalimide OGT2492 (**4**)^18^ in Figure 1. Despite the promising initial characterisation, no small-molecule inhibitor was further advanced to clinical trials. Recently, a combined academic/industrial research project was pursued aimed at the identification and development of new small-molecule heparanase inhibitors. Functionalization of diphenylurea and benzazol-5-yl acetic acid derivatives with fluorine atoms and select amino acids led to inhibitors able to reduce cancer cell invasiveness^19^^,^^20^. Additionally, naphthyl-sulphonylureas were designed^21^ combining structural elements of the antiangiogenic/antitumor sulphonic distamycin-A derivative FCE27266^22^ with those of the heparanase inhibitor suramin^23^. Also considering these results, the chemical space of heparanase inhibitors is still confined to few chemical classes, which is a limitation for the development of effective and drug-like new compounds to be progressed towards clinical trials.

To look for new small-molecule inhibitors with novel structures that could serve as hit/lead compounds for chemical optimisation and drug development, we performed a virtual screening on libraries of commercially available compounds. Ligand-based similarity searches were initially performed using known structurally diverse inhibitors as reference compounds, to identify a first set of compounds to be purchased and tested. Application of an iterative procedure, in which newly discovered inhibitors became reference structures in the subsequent similarity-search runs, allowed to expand the chemical space of selected compounds, identifying new classes of potent inhibitors. The most potent compounds thus identified were further characterised for their biological activity, evaluating the antiproliferative potential and ability to modulate tumour cell invasiveness and to affect expression of genes associated with angiogenesis and tumour growth.

### Experimental section

#### Virtual screening

Compounds evaluated as heparanase inhibitors were taken from eMolecules^24^ and SciFinder^25^ collections of commercially available compounds. Only eMolecules compounds with molecular weight ≥600 were considered (50,768 compounds), as most heparanase inhibitors are characterised by high molecular weight.

Similarity searches were conducted using different reference compounds, initially selected from literature, and then from newly discovered potent inhibitors. Linear fingerprints^26^^,^^27^ were calculated for reference inhibitors and for screened compounds with Canvas v2.6^28^ and the Tanimoto similarity index^29^^,^^30^ was used to rank the compounds. The first 100 compounds of each search were evaluated. Besides similarity score, additional criteria guided compound selection, comprising the presence of certain functional groups (e.g. carboxylic or sulphonic acids, as in substrate and known inhibitors) and compound availability from reliable vendors. In Supplementary Table S1 the reference inhibitor and the similarity index are reported for each compound that was purchased and tested for anti-heparanase activity.

**Figure 2.**
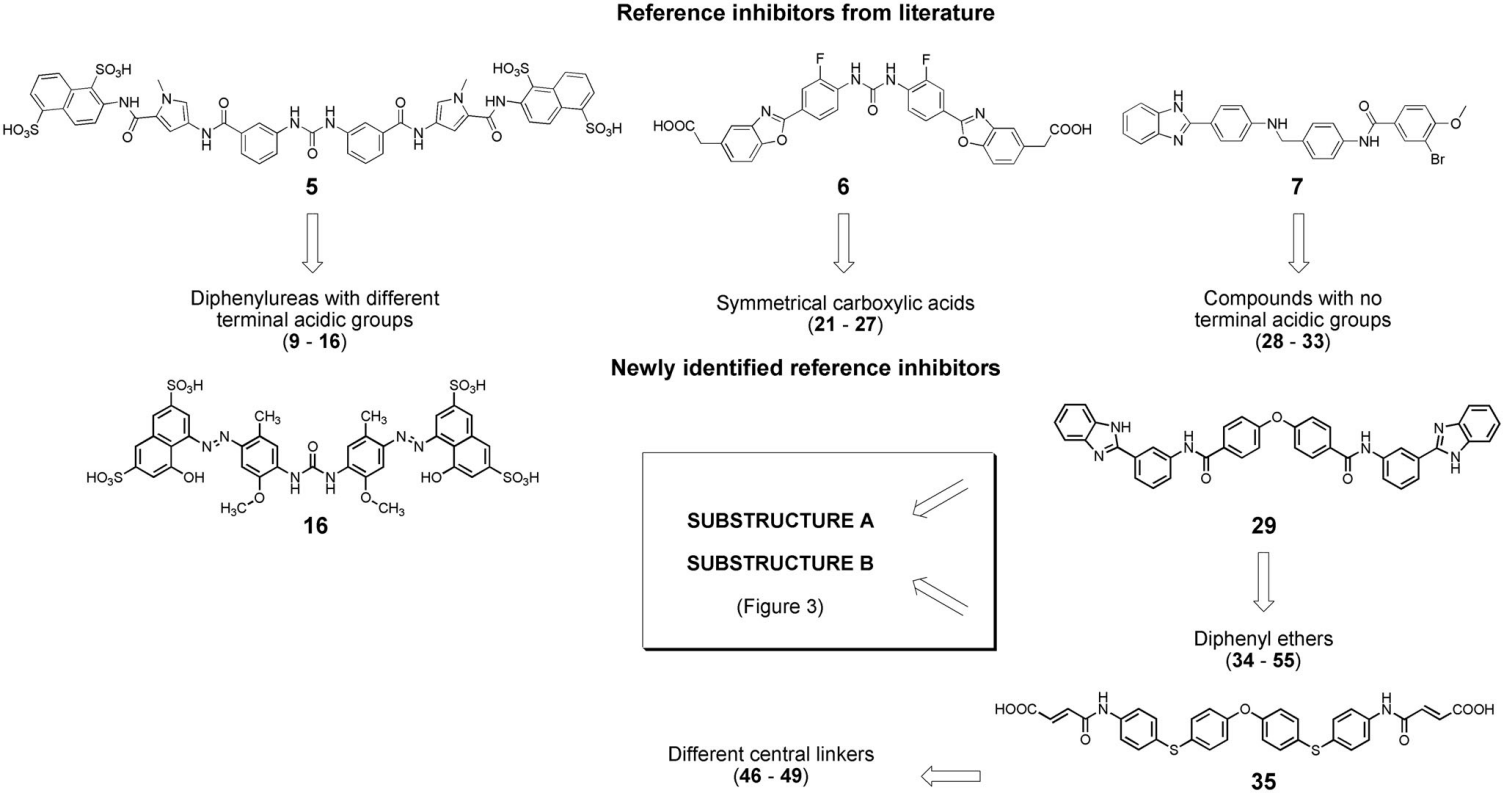
Workflow applied for similarity searches in eMolecules database. Literature inhibitors were first used as reference compounds, then highly potent newly identified inhibitors served as reference structures.

The first virtual screening runs were conducted on eMolecules subset of compounds, using inhibitors **5**, **6**, **7**, **16**, **29** and **35** as reference structures. Compounds **5**^21^ and **6**^20^ are diphenyl urea derivatives and compound **7**^16^ is a (benzimidazol-2-yl)phenylamino derivative with reported IC_50_ values of 0.86, 0.18 and 0.23 µM, respectively. The other reference compounds **16**, **29** and **35** (Figure 2) were identified during the virtual screening procedure. In particular, using compound **5** as reference, we selected symmetrical diphenylurea derivatives carrying different acidic groups (sulphonic acids or bioisosteric phosphonic acids, **9**–**16** in Supplementary Table S1). Inhibitor **6** was used to retrieve symmetrical compounds with at least two carboxylic acids (**21**–**27**) and inhibitor **7** led to compounds **28**–**33**, lacking acidic groups at their terminal portions. Similarity search with reference inhibitor **16** led to the selection of sulphonic acid derivatives **17**–**20**. Similarity with inhibitor **29** led to symmetrical diphenyl-ether derivatives, with (**34**–**37**) or without (**38**–**45**) terminal carboxylic groups. Similarity with compound **35** allowed to retrieve compounds **46**–**49** with different central linkers (i.e. O, SO_2_, isopropyl).

**Figure 3.**
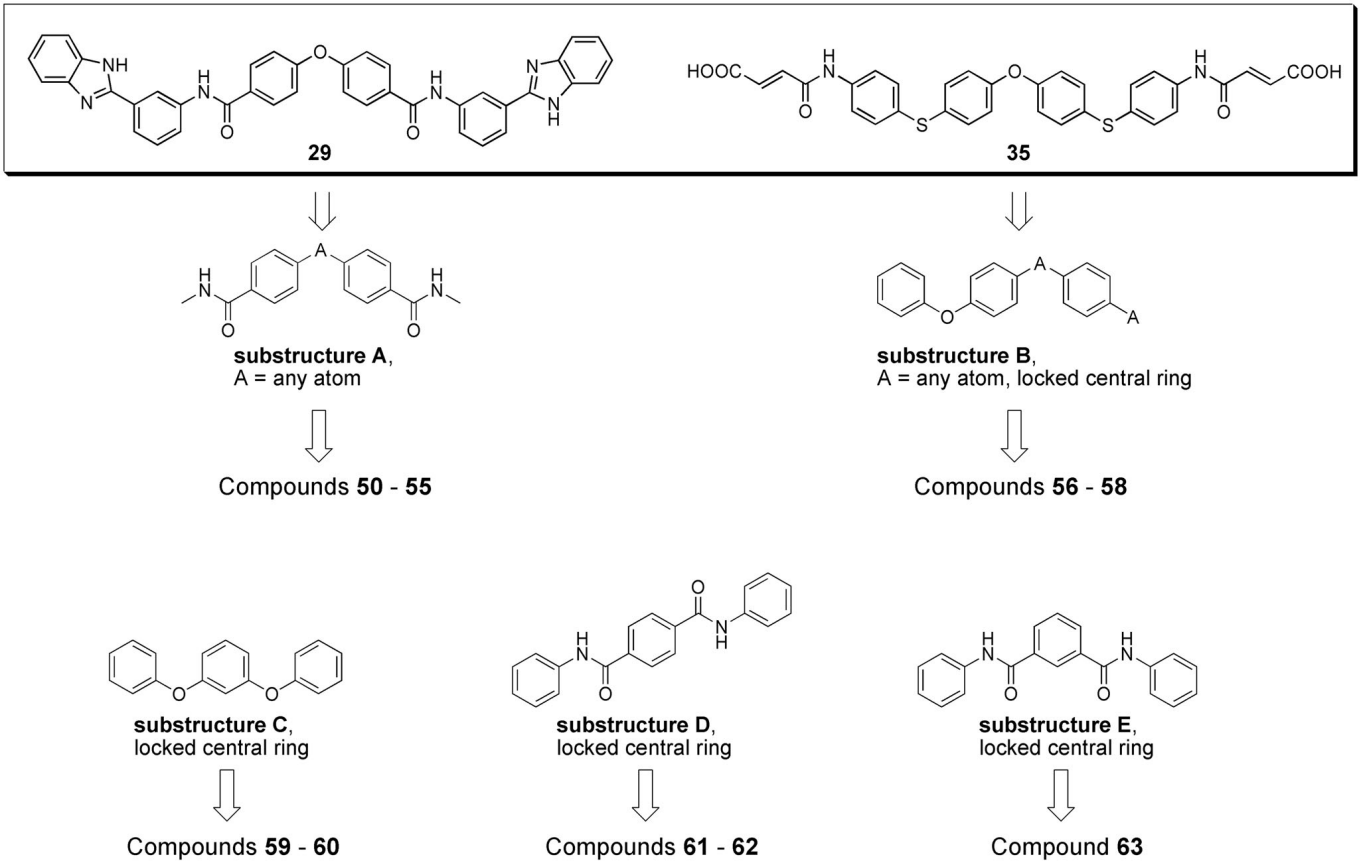
Workflow and query substructures used for virtual screening of heparanase inhibitors performed on SciFinder subset of commercially available compounds.

SciFinder software and database were used to perform substructure searches, looking for commercially available compounds having substructures present in potent inhibitors (substructures **A** and **B** in compounds **29** and **35**, Figure 3) or defined by us as structural variations of substructures **A** and **B** (substructures **C**–**E** in Figure 3). Virtual screening was limited to compounds with molecular weight between 500 and 800, to look for compounds with improved ligand efficiency compared to those selected from eMolecules database. The structures of compounds fulfilling the cited criteria were downloaded as sdf files (117 with substructure **A**, 415 with substructure **B**, 208 with substructure **C**, 210 with substructure **D** and 155 with substructure **E**), linear fingerprints were calculated, and compounds were ranked according to their Tanimoto similarity with the previously employed reference inhibitors **29** and **35**, or, following an iterative approach, with the new inhibitor **57** discovered in this phase (Supplementary Table S1). Compounds with highest similarity index and available from vendors were purchased and tested for anti-heparanase activity. In particular, compounds **50**–**55** were selected through substructure **A** and similarity with reference inhibitor **29**; compounds **56**–**58** were selected through substructure **B** and similarity with reference inhibitor **35**; compounds **59**–**60**, **61**–**62**, and **63** were identified through substructures **C**, **D** and **E**, respectively, and similarity with the new inhibitor **57**.

PAINS potential of the compounds tested as heparanase inhibitors was evaluated applying PAINS filters A, B and C described in ref. 44 implemented in Canvas v.2.6. Filter A captured azo derivatives **13**–**20**, filter C compounds **51**, **53**, and **55**. Application of filter B retrieved no compound.

### Molecular modelling

#### Docking studies

Docking was performed on a previously developed model of human GS3 heparanase^19^ obtained from the crystal structure with PDB code 5E9C in which heparanase is co-crystallised with the tetrasaccharide inhibitor Dp4^31^. The docking grid was built using Glide 6.9^32–33^ and it was centred on Dp4. The dimensions of the bounding and enclosing boxes were set to 20*20*20 Å and 55*55*55 Å, respectively. Ligands were built with Maestro 10.4^34^ and prepared with LigPrep 3.6^35^. For each ligand, a docking run was performed setting MAXKEEP and MAXREF parameters, which control the number of poses to retain after the rough scoring stage and the number of poses to refine, to 50,000 and 4,000, respectively. A full force field post-docking minimisation was performed on the best 500 poses ranked according to their Emodel score, and the final 15 best scoring poses were retained for each ligand.

#### Cluster analysis of docking poses based on structural interaction fingerprints (SIFt)

A cluster analysis was performed on the binding poses obtained for compounds **21, 24, 29, 34, 35, 36, 48, 49, 50, 57, 58** and **59** to look for common binding modes (see text). Structural interaction fingerprints (SIFt)^36^^,^^37^ with the protein were generated for the top 15 Emodel-ranked docking poses of each compound using the Interaction Fingerprint tool provided within the Chemoinformatics scripts of Maestro 10.4. Both polar and hydrophobic contacts between ligand and protein were considered to generate the SIFt, and the criteria to define the interacting region and the interaction distance cut-offs were maintained to their default values. The docking poses of all compounds were then clustered, based on the Tanimoto similarity of their SIFt applying the average linkage method^38^. The dendrogram tree was cut at 20 clusters, to get a compromise between high SIFt similarity and high population of the clusters. Among these, the cluster with the highest number of compounds included (10 out of 12) was selected.

### Chemicals

Compounds were purchased from different vendors. For each compound, the vendor and the declared purity are reported in Supporting Table S2. HPLC-UV determination of purity and ESI mass spectra of the four most potent compounds **16**, **29**, **61** and **63** are reported in Supporting Information material. ^1^H NMR spectra of the most potent heparanase inhibitors (see Table 1 for structures) were collected in Supporting Information.

**Table 1.** Heparanase inhibitory activity (fondaparinux assay on human GS3 heparanase) of the most potent commercially available compounds selected through virtual screening.

Compd.	Molecular formula	Molecular weight (neutral species)	IC_50_ (µM)^a^ ± SD
**suramin**		1297	26.6 ± 0.10
**7**^16^		527	0.37 ± 0.02
**8**^15^		501	0.56 ± 0.03
**15**		1241	0.93 ± 0.07
**16**		961	0.37 ± 0.01
**19**		757	1.66 ± 0.12
**20**		903	1.76 ± 0.23
**21**		654	2.66 ± 0.32
**24**		835	1.10 ± 0.13
**29**		641	0.38 ± 0.04
**34**		733	3.15 ± 0.28
**35**		613	0.52 ± 0.02
**36**		821	0.63 ± 0.01
**48**		759	0.91 ± 0.10
**49**		759	2.35 ± 0.23
**50**		584	2.01 ± 0.13
**57**		641	2.17 ± 0.18
**58**		641	3.78 ± 0.22
**59**		641	2.08 ± 0.16
**61**		549	0.32 ± 0.06
**62**		677	3.13 ± 0.27
**63**		763	0.12 ± 0.01

^a^ Dose causing 50% inhibition of heparanase enzymatic activity as determined from dose-response curves (mean of triplicates; SD always <10%) repeated at least twice in separate experiments.

Suramin, compound **7** and compound **8** are included as reference inhibitors.

### Biological methods

#### In vitro *screening for heparanase activity*

To determine the activity of the heparanase inhibitors, a homogeneous assay based on the cleavage of the synthetic heparin oligosaccharide fondaparinux (Arixtra; Aspen) was used. The assay measures colorimetrically the appearance of the disaccharide product of heparanase-catalysed fondaparinux cleavage using the tetrazolium salt WST-1. The assay was essentially performed as described^39^ with minor modifications. Briefly, Nunc 96-well (Thermo Fisher Scientific) plates were pre-treated with a solution of 4% BSA (bovine serum albumin, Sigma-Aldrich) in phosphate-buffered saline containing 0.05% Tween 20 (PBST), for 2 h at 37 °C, and then washed three times with PBST. The assay was carried out with 100 µL per well of assay solution containing 40 mM sodium acetate buffer (pH 5.0), 100 μM fondaparinux, 2.5 nM recombinant heparanase (GS3), and serial dilutions of test compounds (tested in triplicate). Plates were sealed with adhesive tape and incubated, in the dark, for 3 h at 37 °C, followed by developing with 1.69 mM WST-1 (Santa Cruz Biotechnology), for 1 h at 60 °C. Then, the absorbance at 560 nm was measured through a microplate reader (Victor 3, PerkinElmer). The IC_50_ value for each compound was calculated by GraphPad software. Finally, the measurements were corrected by subtracting both the reagent background and the inner absorbance value of test compound.

#### Cell lines and maintenance

HT1080 (fibrosarcoma), U87MG (glioblastoma), and U2OS (osteosarcoma) human cell lines were purchased from the American Type Culture Collection (ATCC; Manassas, VA) and maintained according to the manufacturer’s recommendations. Briefly, HT1080 and U87MG were grown in modified Eagle medium (Thermo Fisher Scientific), while U2OS cells were grown in McCoy’s 5a medium (Thermo Fisher Scientific), all supplemented with 10% FCS (Thermo Fisher Scientific), 100 U/mL penicillin, and 100 μg/mL streptomycin (Thermo Fisher Scientific) and 2 mM L-glutamine (Thermo Fisher Scientific). Cell lines were maintained at 37 °C with a humidified 5% CO_2_ atmosphere.

#### Proliferation assay

HT1080, U87MG, and U2OS exponentially growing cells were seeded into 96-well plates and then, 24 h later, treated for 72 h with several dilutions of test compounds in the range 10 − 0.2 μM. Inhibition of cell proliferation was measured by means of a classical sulforhodamine B (SRB) assay^40^ performed in triplicate. The drug concentrations able to inhibit cell proliferation by 50% (IC_50_) were ultimately calculated from dose − response curves by using the GraphPad Prism software.

#### Matrigel invasion assay

Cells were pre-treated with the indicated drug concentrations in complete medium for 24 h. Then, cells were harvested, resuspended in serum-free medium, and transferred (2.5 × 10^4^ cells per filter) to the upper chamber of 24-well Transwell plates (Costar, Corning Inc., Corning, NY) previously coated with Growth Factor Reduced Matrigel (BD Biosciences, San Jose, CA). The same drug concentration used for cell pre-treatment was added to both the upper and lower chambers. After 24 h of incubation, cells that invaded the Matrigel were stained with SRB and then counted through a microscope at 40× −100× magnification depending on cell density. Data were expressed as the % inhibition of cell invasion, with respect to cell invasion in the absence of drugs.

#### Real-Time qPCR assay

Total RNA was extracted from HT1080 cells, upon 24 h of treatment with test compounds (1 µM) and then retrotranscribed using the iScript Advanced cDNA Synthesis Kit for RT-qPCR (Bio-Rad Laboratories Inc., Hercules, CA) according to the manufacturer’s instructions. Real time quantitative PCR analysis was performed using SsoAdvanced Universal SYBR Green Supermix (Bio- Rad Laboratories Inc., Hercules, CA) and the following PrimePCR SYBR Green Assays: qHsaCID0015228 (heparanase), qHsaCED0002206 (FGF-1), qHsaCID0015510 (FGF-2), qHsaCID0011597 (MMP-9), and qHsaCED0043454 (VEGF-A) (Bio-Rad Laboratories Inc., Hercules, CA). The 7900HT Sequence Detection System instrument and software (Applied Biosystems) were used to quantify the relative expression of the target genes. Tests were performed in triplicate.

## Results and discussion

### Virtual screening and identification of new classes of heparanase inhibitors

Compounds evaluated for anti-heparanase activity were selected from eMolecules^24^ and SciFinder^25^ databases of commercially available compounds, considering their structural similarity, based on linear fingerprints, with potent inhibitors. In particular, similarity searches were initially conducted on a subset of eMolecules database comprising compounds with molecular weight higher than 600. We chose to start from this subset given the high molecular weight of most potent heparanase inhibitors^4^. In fact, many known non-glycosidic inhibitors are composed of a central scaffold with two long arms that are likely accommodated within the peripheral heparin-binding domains HBD-1 and HBD-2. We focussed the screening process on larger compounds to find new ligands with original structures, yet maintaining these features to allow a rationalisation of their structure-activity relationships by molecular docking. 2D linear fingerprints and Tanimoto similarity with reference inhibitors were calculated for each compound. Selection was driven by similarity score, but visual inspection of the best-scoring compounds led us to apply other qualitative and practical criteria, such as the presence of functional groups typical of heparanase inhibitors (e.g. acidic groups) and the availability of compounds from reliable vendors. Six consecutive runs of similarity search were performed. Initially, known inhibitors reported in the scientific literature (compounds **5**–**7**^21^^,^^20^^,^^16^ in Figure 2) were used as reference structures. Then, newly discovered inhibitors with high potency became the new reference compounds (compounds **16**, **29** and **35** in Figure 2) for subsequent search runs, to widen the diversity of selected compounds.

Additionally, substructure queries and similarity searches were performed on SciFinder subsets of commercially available compounds with the double aim to further increase the structural diversity and to improve ligand efficiency of the selected compounds. In particular, starting from the wide range of molecular weights (MWs) of the most potent inhibitors previously identified from eMolecules database (compounds **15**–**49** in Table 1 with 613 ≤ MW ≤ 1241), we decided in this phase to limit the similarity searches to compounds with MW comprised between 500 and 800. Substructure searches were first performed looking for compounds having structural motifs observed in potent inhibitors retrieved in the previous phase (eMolecules search, substructures **A** and **B** in Figure 3) or defined by us as variants of such motifs (substructures **C**–**E** in Figure 3). Then, linear fingerprints and Tanimoto similarity with reference inhibitors were calculated for compounds thus retrieved. The available compounds with highest similarity index were purchased and tested.

Overall, fifty-five compounds were purchased and evaluated for their anti-heparanase activity, forty-one retrieved from the similarity searches on eMolecules collection and fourteen from the substructure + similarity searches on SciFinder database.

Supporting Table S1 reports the structures of the fifty-five compounds purchased and tested, with information, for each compound, on the database from which it was retrieved, the reference inhibitor used to retrieve it and the similarity value with the reference inhibitor. Compounds were first tested on recombinant GS3 heparanase with a fondaparinux assay (see Experimental) at the fixed concentrations of 25 and 2.5 µM, and IC_50_ values were calculated from dose-response curves for the most promising ones. The most potent inhibitors, with IC_50_ values lower than 5 µM, are reported in Table 1 and their dose-response curves are depicted in Supplementary Figure S1.

At the beginning of the screening procedure, similarity with the suramin derivative **5** (Figure 2, IC_50_ = 0.86 µM)^21^ led to the selection of eight compounds (**9**–**16**), with the most interesting two (**15** and **16**) having diazene linkers. The most potent inhibitor (**16**, IC_50_ = 0.37 µM) was used as the reference compound for the following run which led to the small subset of four compounds **17**–**20**, where the last two had IC_50_ values in the low µM range. All compounds **9–20** were characterised by a central diaryl urea portion variously substituted with sulphonic or phosphonic acids. Inhibitory data for these compounds show that heparanase is rather tolerant to the number and position of anionic groups surrounding the diaryl urea core, as sulphonic groups can be present not only on the terminal portions of the molecule, but also on central rings (e.g. **13** in Table 1). In this class of inhibitors, which was already known, terminal acidic groups are not essential, but they appear to increase potency.

Virtual screening based on similarity with the known benzoxazolyl-phenyl-urea **6** (IC_50_ = 0.18 µM)^20^ led to the selection of seven polycarboxylic acid derivatives (**21**–**27**). These compounds are characterised by a symmetrically substituted central linker different from the urea group, i.e. a diphenyl ether, a diphenyl sulphone or a diaryl ketone. While some diphenyl ethers had already been described as heparanase inhibitors in patent applications^41^^,^^42^ , this is the first time that anti-heparanase activity is reported in a peer-reviewed scientific paper for such compounds. More importantly, the diphenyl sulphone **24** (IC_50_ = 1.10 µM) can be considered the prototype of a new class of heparanase inhibitors where the sulphone group behaves as a bioisostere of the urea fragment and of the central oxygen atom in diphenyl ethers. In fact, a diphenyl ether having the same symmetrical substitution as compound **24** (**23** in Supplementary Table S1) also showed significant inhibition of heparanase at 2.5 µM.

The third reference compound taken from the literature was the neutral asymmetric inhibitor **7** (IC_50_ = 0.23 µM^16^ which led to the selection of compounds **28**–**33**, which did not present acidic groups at their terminal portions. Remarkably, an IC_50_ value of 0.38 µM was observed for the diphenyl ether **29**, one of the few inhibitors devoid of acidic groups with potent activity on heparanase. Compound **29** was used as the reference structure for an additional similarity search in eMolecules database and allowed to select further twelve diphenyl ethers (**34**–**45** in Supplementary Table S1), including three (**34–36**) with IC_50_ values lower than 5 µM. In this group, compounds without a terminal carboxylic fragment had lower inhibitory efficacy compared to acidic derivatives (Supplementary Table S1). The most potent compound **35** differs from the other diphenyl ethers, having one-atom linkers (sulphur atoms) connecting the central diaryl portion to substituents, instead of amide-like fragments. The different geometry and the good inhibitory potency of compound **35** prompted its use as a reference for the search of similar compounds. Out of the four compounds purchased and tested (**46**–**49**), two acidic derivatives (**48** and **49**) had significant inhibitory potencies. Interestingly, these two compounds are characterised by a central isopropyl linker replacing the oxygen atom of the reference diphenyl ether core, and such a motif had never been reported for heparanase inhibitors. While heparanase inhibitors with a similar diphenylmethane structure have been described in a patent application,^42^ these new derivatives with the dimethylated hinge demonstrate the high tolerance of the enzyme for the central portion of symmetric inhibitors.

Finally, substructure searches followed by similarity with the new inhibitors previously identified were performed on SciFinder collection of commercially available compounds. The substructures used to retrieve lists of compounds to be evaluated for purchasing are reported in Figure 3. Substructures **A** and **B** are present in the potent inhibitors **29** and **35**, respectively. The lists of commercially available compounds with MW between 500 and 800 were screened calculating structural similarity with previously discovered inhibitors **29** and **35**, and the available best scoring compounds were purchased and tested. Substructure **A** led to the selection of six compounds (**50**–**55** in Supporting Table S1) which were acidic diphenyl ethers or diphenyl sulphones, some of which (**50** and **53**) endowed with good inhibitory potencies. Substructure **B** led to the identification of three compounds (**56**–**58**, in Supplementary Table S1) with both the para-substituted phenolic diethers **57** and **58** showing IC_50_ values lower than 5 µM. Given the good inhibitory potencies and the structural novelty of these symmetrical para-substituted phenolic diethers, we decided to look for meta-substituted analogues (substructure **C**) and for analogues where the link atoms of substructures **B** and **C** were replaced by amide linkers in para (substructure **D**) or meta (substructure **E**) position. Substructure **C** allowed to retrieve two meta-substituted ethers (**59** and **60** in Supplementary Table S1) including compound **59** which showed good inhibitory potency. The most potent inhibitors identified through virtual screening based on substructures **D** and **E** are characterised by a terephthalamide (**61**) or an isophthalamide (**63**) central fragment, respectively, symmetrically substituted with heparanase-binding motifs. The acidic isophthalamide derivative **63**, with an IC_50_ = 0.12 µM, is one of the most potent small-molecule inhibitors of heparanase reported so far. Overall, virtual screening allowed to identify several new compounds endowed with high heparanase inhibitory potencies, with some of them belonging to new chemical classes, i.e. diphenyl sulphones, 1,1-dimethyl-diphenylmethane derivatives and symmetrical compounds with a central phenyl ring.

Substructure filters for Pan Assay Interference Compounds (PAINS)^43^ applied to the fifty-five compounds tested as heparanase inhibitors suggested that the eight azo derivatives **13**–**20** and compounds **51**, **53** and **57** could be potentially problematic. Nevertheless, the azo derivatives have been included in the screening steps given their high structural similarity with known inhibitors and their potential to increase structural diversity of selected compounds when used as reference inhibitors in similarity searches. In fact, as the main aim of this research was to identify novel chemotypes to be further developed, we did not exclude azo derivatives such as compound **16** that may be converted into suitable derivatives by bioisosteric replacement, e.g. with an amide fragment. Compounds **51** and **53** were captured by PAINS filter given the presence of a 2-amido-5-hydroxybenzoic acid fragment, and compound **57** being a phthalimide 5-carboxylic acid derivative. However, the potential PAINS behaviour of compounds carrying these last two fragments may be overestimated, as each of them was present in a very limited number of the compounds originally used to define the PAINS filter^44^. On the other hand, the hydroxyl substituent is dispensable from the structure of anthranilic acid inhibitors **51** and **53**, as activity is maintained by analogues with a bromine replacing the hydroxyl group (compound **21**) or by anthranilic acids with no substituents on the phenyl ring (compound **23**). As for the phthalimide fragment of compound **57**, there are several examples of 2,3-dihydro-1,3-dioxo-1*H*-isoindole-5-carboxylic acid derivatives described as potent heparanase inhibitors (e.g. compound **4** in Figure 1 and ref.^18^) which suggests that this fragment gives specific interactions with heparanase and not a generic pan-inhibitory effect.

**Figure 4.**
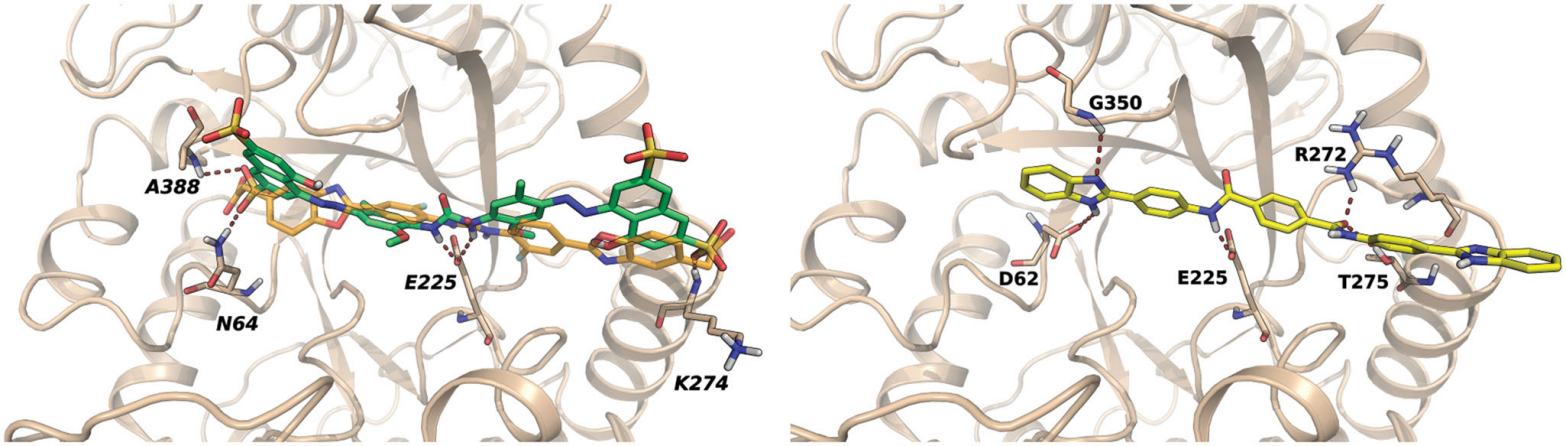
Left: best docking pose obtained for sulphonyl-diphenyl urea **16** (green carbons). The docking pose of the reference inhibitor **6** (orange carbons) is shown for comparison. Right: docking pose obtained for terephthalamide derivative **61**.

To investigate the putative binding mode of the most potent heparanase inhibitors, compounds in Table 1 were docked into the substrate binding site of human heparanase. Mature heparanase is a heterodimer composed of an N-terminal 8 kDa chain and a C-terminal 50 kDa chain, produced by proteolytic cleavage of the precursor single-chain proheparanase. Crystal structures of human heparanase show a (β/α)_8_-TIM barrel fold, in which the two chains are non-covalently assembled. The substrate binding region is a narrow channel comprising the catalytic site in which glutamates E225 and E343 reside. Close to the catalytic site is the glycine loop (G349 and G350) which favours the proper arrangement of the substrate by interacting with the carboxylate of glucuronic acid. The catalytic site is flanked by two regions, known as heparin-binding domains (HBD-1: residues 158 − 171, HBD-2: residues 270 − 280), rich in basic residues and identified as protein motifs involved in the interaction with the substrate HS^44^. As the inhibitory activity of the selected compounds was evaluated on human GS3 heparanase, i.e. a recombinant heparanase in which the N- and C-terminal chains are connected through a short peptide^45^, docking studies were performed with Glide software on a model of GS3 heparanase previously prepared from the X-ray structure of crystallised heparanase^19^. In their best scoring poses, diaryl ureas **13**, **15**, **16**, **19** and **20** adopted a similar binding mode, placing the urea group in proximity of residue E225. The side chains of these compounds extend towards HBD-1 and HBD-2 and form several interactions with residues of the substrate binding site. Figure 4 (left) shows the most potent urea derivative **16** docked into heparanase substrate-binding site. The urea group occupies the catalytic site, with the nitrogen atoms interacting with E225 and the sulphonic acids forming polar interactions with N64 and A388, which also serve as binding partners for the sulphate groups of co-crystallised substrates and inhibitors^31^, and with the backbone of K274 from HBD-2. The binding mode of compound **16** is consistent with that obtained for the reference diphenylurea inhibitor **6** (Figure 2)^20^ having terminal benzoxazolyl-acetic groups instead of naphthylsulfonates. Compounds **61**, **62** and **63**, having a planar central portion of iso- or terephthalamide, accommodate into the substrate binding site adopting an overall arrangement similar to that of diaryl ureas, as can be seen for compound **61** in Figure 4 (right) in which an amide group interacts with E255 and the terminal benzimidazole is captured under the glycine loop by hydrogen bonds with G350 and D62. The other benzimidazole ring interacts with residues from HBD-2.

**Figure 5.**
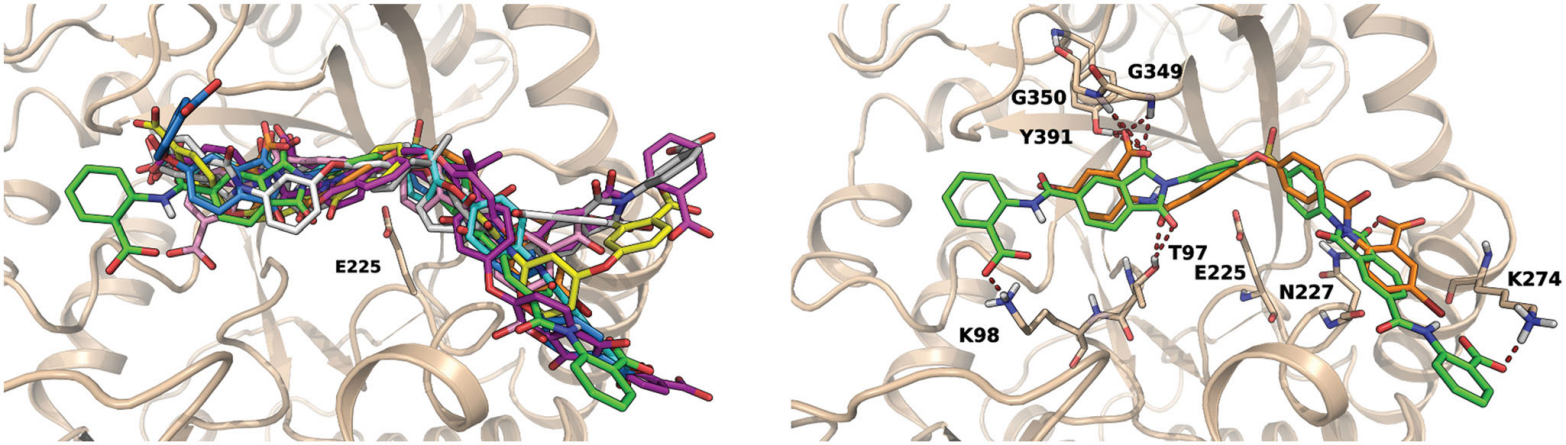
Left: overall superposition of docking poses obtained for compounds **21** (orange), **24** (green), **34** (light blue), **36** (yellow), **48** (gray), **49** (purple), **50** (pink), **57** (cyan), **58** (magenta) and **59** (white) selected through cluster analysis performed on structural interaction fingerprints. Right: superposition of best docking poses obtained for compounds **21** (orange carbons) and **24** (green carbons).

Several inhibitors in Table 1 are characterised by a bent, V-shaped structure of the central region, given by the presence of single-atom junctions between aromatic portions (e.g. O, S) or of functional groups (e.g. SO_2_, isopropyl) favouring a non-linear arrangement of the molecule. Docking experiments provided multiple solutions for these compounds, spanning the substrate binding site. To look for common binding modes and ligand-protein interactions, we performed a cluster analysis on multiple docking solutions obtained for compounds **21**, **24**, **29**, **34–36**, **48–50** and **57–59**, looking for poses that could give similar interactions with the protein. In particular, structural interaction fingerprints (SIFt)^37^, consisting of strings indicating the presence of ligand-protein interaction features, were calculated for the top fifteen poses of each inhibitor, ranked by the Emodel docking score. Cluster analysis performed on SIFt identified a cluster of poses with ligand-protein interactions conserved for ten compounds, out of the twelve submitted to the analysis. Figure 5 depicts the overall superposition of inhibitors (left image) and the docking arrangement obtained for compounds **21** and **24** only (right image), to appreciate the interactions undertaken by the ligands. The bent portion of the inhibitors points towards the catalytic site where the oxygen and the sulphonyl linkers of **21** and **24** are positioned. One phthalimide ring of **24** interacts with the glycine loop and Y391, reproducing the interactions undertaken by the carboxylate of substrate glucuronic acid, and with T97 on the opposite side. The glycine loop is one of the main interaction elements for these compounds, forming hydrogen bonds with carbonyl groups either inserted in phthalimide rings (e.g. **24**, **34**, **48**, **57**) or belonging to amide linkers (e.g. **50**). Alternatively, the glycine loop serves as an anchoring point for negatively charged carboxylates (e.g. **21**, **58**). The portion of compound **24** on the right of Figure 5 is bound to N227 and K274. N227 is the preferred partner for the carbonyl groups of phthalimide rings occupying this region, as is also targeted by compounds **36**, **57** and **59**. Another relevant residue belonging to HBD-2 is K274, positioned at the end of an α-helix, which interacts with compounds **24**, **36**, **48**, **58** and **59** with either its side chain or backbone NH. The isopropyl group of compounds **48** and **49** is not accommodated inside the catalytic site, where an oxygen atom is present; it occupies a more external position where the two methyl substituents are tolerated.

Among the twelve compounds submitted to docking calculations and cluster analysis, only compounds **29** and **35** did not share this common binding arrangement. Compound **29** differs from the other V-shaped inhibitors in that it is neutral at physiological pH and this may affect its accommodation and interactions. Compound **35** has a unique structure compared to inhibitors reported in Table 1, which favours interactions with other residues of heparanase. In particular, without interacting directly with the central glycines 349 and 350, it assumes a bent shape which allowed peripheral polar interactions between its carboxylate groups and the side chains of Q270 and R272 in HBD-2. The other moiety of compound **35** protrudes beyond HBD-1, taking a polar interaction with S163. However, both compounds **29** and **35** occupy the catalytic site interacting with E225. The best poses obtained for compounds **29** and **35** are represented in Supporting Figure S2.

These docking models suggest that the new inhibitors here reported can inhibit heparanase by different interaction mechanisms: diaryl ureas and iso- or terephthalamides are arranged in a planar conformation extending from HBD-1 to HBD-2, while diaryl ether, sulphone and isopropyl derivatives can occupy, in a bent conformation, HBD-1 similarly to planar inhibitors and HBD-2 with a slightly different orientation. These binding hypotheses can be useful to optimise the structure of novel hit/lead compounds here reported.

### Evaluation of biological activity

#### Proliferation assay

Four compounds characterised by IC_50_ values < 0.5 µM in the fondaparinux assay (**16**, **29**, **61** and **63**) were further evaluated for their antiproliferative activity on three human cancer cell lines, HT1080 (fibrosarcoma), U2OS (osteosarcoma), and U87MG (glioma) which express different levels of heparanase^46^^,^^47^^,^^48^, and their behaviour was compared with that of two reference compounds, suramin and compound **7**. Cells were treated for 72 h with serial dilutions of each test compound (in the range 10–0.2 µM) covering the active concentrations in the heparanase enzyme assay and the antiproliferative activity was evaluated with the sulforhodamine B assay. None of the compounds showed antiproliferative activity up to the maximum concentration assessed (10 µM). The same applies to suramin, while the other reference inhibitor **7** moderately inhibited proliferation of the three cell lines (IC_50_ values: 3.1 ± 0.3 µM on HT1080, 2.7 ± 0.1 µM on U87MG and 2.1 ± 0.2 µM on U2OS cell lines, respectively). Even if we have not tested inhibitor potency on heparanase from cell lysates, these data show that the newly identified compounds **16**, **29**, **61** and **63** are able to inhibit recombinant heparanase activity at concentrations at which they do not have any interference with cell proliferation, making their use potentially advantageous for therapeutic applications in non-oncology fields, such as inflammatory and autoimmune diseases.

#### Invasion assay

Given the relevance of heparanase in driving cancer cell invasion and metastasis^49–50^, compounds **16**, **29**, **61** and **63** were further evaluated for their ability to inhibit the invasive potential of HT1080, U87MG and U2OS cell lines. Compounds were tested in the Matrigel cell invasion assay at a fixed concentration that allowed inhibition of heparanase enzymatic activity without significantly affecting cell proliferation. As can be seen in Table 2, compounds showed a different ability to inhibit cancer cell invasiveness depending on the cell type. In particular, the diphenyl urea **16** was more effective on HT1080 cells than on U87MG and U2OS at the high concentration tested (10 µM). Importantly, the two compounds belonging to the novel class of tere- and isophthalamides (**61** and **63**, respectively) showed a significant activity on U2OS cell line, comparable to that of reference heparanase inhibitor **7**.

**Table 2.** Inhibition of invasive activity of HT1080, U87MG, and U2OS human cancer cell lines by newly discovered heparanase inhibitors.^a^

Compd.	Concentration tested (µM)	Invasion assay inhibition (%)		
		HT1080	U87MG	U2OS
suramin	10	22 ± 5	–	–
7	1.0	88 ± 2	78 ± 3	82 ± 2
16	10	93 ± 1	10 ± 4	–
29	2.5	29 ± 4	15 ± 3	18 ± 3
61	2.5	41 ± 3	43 ± 5	80 ± 2
63	2.5	39 ± 6	45 ± 4	75 ± 3

^a^ Matrigel cell invasion assay on HT1080, U87MG, and U2OS tumour cells upon 24 h of treatment. Each drug was tested in triplicate. “–” no inhibition.

#### Effects on gene expression

**Figure 6.**
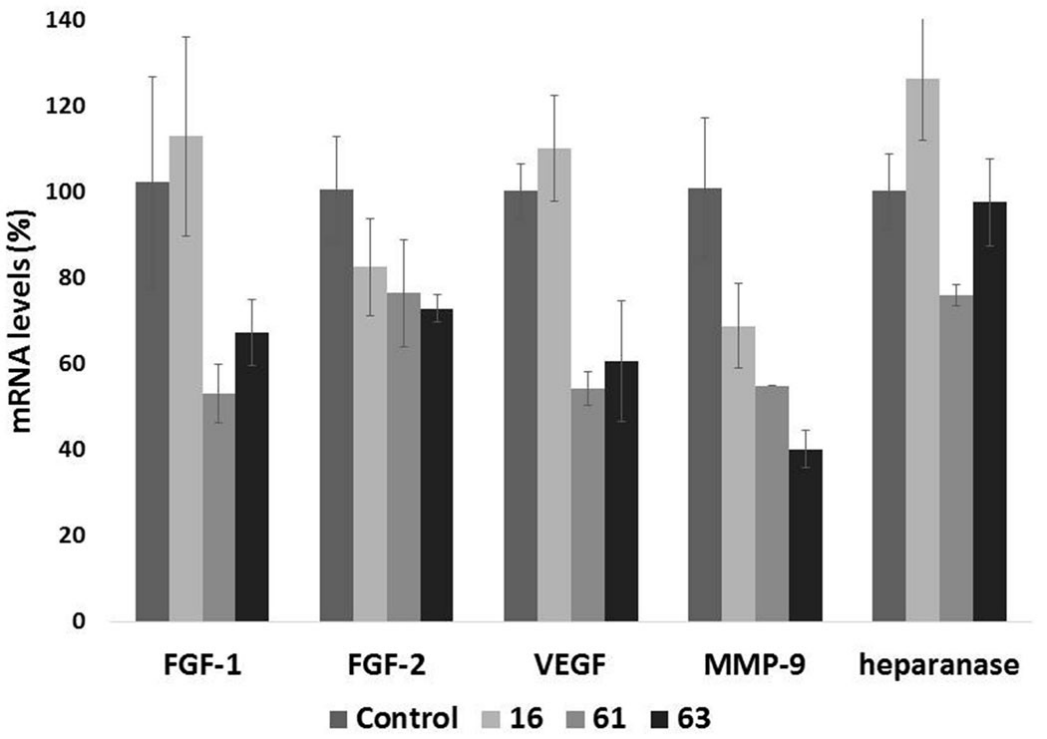
Effect of compounds **16**, **61** and **63** (1 µM) on the expression of select angiogenesis-related genes. The expression levels of FGF-1, FGF-2, VEGF, MMP-9, and heparanase mRNA in HT1080 cells, upon 24 h of treatment, were measured through real-time qPCR analysis. Results are expressed as % with respect to untreated cells.

Besides its well-recognized extracellular functions, heparanase has been found actively involved in nuclear activities^51^. In *in vitro* experiments, heparanase reduced nuclear levels of syndecan-1^52^ and modified histone H3 methylation patterns ^53^, promoting expression of a large cohort of genes, such as IL-2, CD69, tissue factor, MMP-9, and VEGF. Activity of such proteins may contribute to heparanase-mediated cell signalling, invasion processes and formation of metastasis. We therefore evaluated the ability of heparanase inhibitors which had shown inhibition of invasiveness higher than 50% in at least a cell line (**16**, **61** and **63**) to affect the transcription of genes encoding for the proangiogenic factors FGF-1, FGF-2, VEGF, MMP-9 and for heparanase. Human fibrosarcoma HT1080 cells were treated for 24 h with 1 µM of the test compounds, a concentration allowing inhibition of heparanase activity but not of tumour cell proliferation and which should avoid off-target effects. mRNA levels of genes encoding for FGF-1, FGF-2, VEGF, MMP-9 and heparanase were measured by a quantitative real-time polymerase chain reaction (q-PCR) assay. Compounds **61** and **63** showed a significant inhibitory effect on transcription of most of the proangiogenic genes, while transcription of heparanase was not or very weakly affected (Figure 6).

## Conclusions

In the last years, a growing body of experimental evidence has indicated heparanase as a valuable target in several pathologies correlated with cell migration and tissue remodelling. In anticancer therapy heparanase is already a validated target, given the extensive studies confirming its involvement in cancer cell growth and spreading, angiogenesis and metastasis. Oligo- and polysaccharide-derived heparanase inhibitors progressed to clinical studies have shown promising results which support the search for new compounds with potential advantages. The main limitation for oligo- and polysaccharides is that their chemical nature prevents oral administration. Moreover, muparfostat and heparin derivatives roneparstat and necuparanib, but not pixatimod, are mixtures of molecules of different molecular weight which can raise problems regarding their characterisation and standardisation. Finally, muparfostat maintains a significant anticoagulant activity^54^. On the other hand, these compounds are highly soluble, and show a potent inhibitory activities (e.g. roneparstat has IC_50_ = 5 nM on human recombinant heparanase^20^). However, given the large difference in molecular weight between small molecules and natural heparin derivatives, their potency expressed in µg/mL are of the same order (e.g. **63**: IC_50_ = 0.12 µM corresponds to 0.09 µg/mL; roneparstat: IC_50_ = 0.005 µM corresponds to 0.10 µg/mL^20^). Moreover, small molecules can be modulated by structure-activity relationship studies to improve their metabolic and distribution properties, and many small-molecule drugs are actually administered by oral route.

After a period in the nineties in which pharma companies and university groups actively worked on new classes of small-molecule inhibitors, the development of such compounds was abandoned. The search for new small-molecule inhibitors has seen a new impulse in the very last years, with the report of new compounds and of improved derivatives of former inhibitors^19–21^^,^^55^^,^^56^. In this context, a virtual screening based on structural similarity with known heparanase inhibitors allowed us to identify several potent compounds belonging to new chemical classes. They comprise diphenyl sulphones, 1,1-dimethyl-diphenylmethane derivatives and symmetrical inhibitors with a central phenyl ring, including isopthalamide and terephthalamide derivatives.

As for the knowledge on the activity of heparanase and its inhibition, the newly discovered compounds confirm an efficient inhibition performed by high molecular weight compounds (Table 1). This is likely due to the ability of elongated compounds to occupy the substrate binding site of the enzyme, preventing the binding of HS chains, rich in functional groups able to interact with the protein surface. Heparanase can be inhibited with high potency by both acidic and neutral compounds (e.g. compound **29**; SST0656AA1). Inhibition by acidic derivatives is consistent with the possibility to mimic the interactions of the substrate with the positively charged residues in the proximity of the active site. On the other hand, efficient inhibition by neutral compounds heavily depends on short-range interactions, like H-bonding and hydrophobic ones, which might be of significant value for the design of new compounds with optimised physicochemical properties.

The screening strategy was effective, as nineteen compounds out of the fifty-five tested showed IC_50_ values lower than 5 µM and four compounds lower than 0.5 µM, including the novel iso- and terephthalamide derivatives **61** (SST0856AA1) and **63** (SST0859AA1). This is a remarkable result in the field of heparanase inhibitors considering the limited number of compounds with submicromolar inhibitory potencies currently reported. Compounds belonging to the new chemical classes represent valuable starting points for the development of optimised derivatives, that can be designed and tailored also on the basis of the information coming from the three-dimensional structure of the enzyme which has recently become available ^31^. These compounds showed not only good enzyme inhibitory potencies, but also promising biological activities, being able to block cancer cell invasiveness and to affect gene transcription as expected from effective heparanase inhibitors.

## Supplementary Material

Supplemental Material
