# Supplementary material for: New classes of potent heparanase inhibitors from ligand-based virtual screening
Source: J Enzyme Inhib Med Chem. 2020 Sep 9;35(1):1685–96. doi: 10.1080/14756366.2020.1811701 (PMC7534336; doi:10.1080/14756366.2020.1811701)

Supporting Information

New classes of potent heparanase inhibitors from ligand-based virtual screening

Daniele Pala,[1#] Laura Scalvini,[1#] Gian Marco Elisi,[1] Alessio Lodola,[1] Marco Mor,[1] Gilberto Spadoni,[2] Fabiana Fosca Ferrara,[3] Emiliano Pavoni,[3] Giuseppe Roscilli,[3] Ferdinando M. Milazzo,[4] Gianfranco Battistuzzi,[4] Silvia Rivara,[1,*] Giuseppe Giannini[4,*]

[1]Dipartimento di Scienze degli Alimenti e del Farmaco, Università degli Studi di Parma, Parco Area delle Scienze 27/A, I-43124 Parma, Italy
[2]Dipartimento di Scienze Biomolecolari, Università degli Studi di Urbino "Carlo Bo", Piazza Rinascimento 6, I-61029 Urbino, Italy
[3]Takis s.r.l., Via Castel Romano 100, I-00128 Roma, Italy
[4]R&D Alfasigma S.p.A., Via Pontina Km 30,400, Pomezia, I-00071 Roma, Italy

- Table S1. Commercially available compounds selected by virtual screening and tested as heparanase inhibitors. Pages 2-9
- Figure S1. Dose-response curves for compounds listed in Table 1. Page 10
- Figure S2. Docking of compounds **29** and **35**. Page 11
- Table S2. List of vendors and purity of tested compounds Pages 12-13
- HPLC-UV assessment of purity and ESI mass spectra of target compounds **16**, **29**, **61** and **63** Pages 14-21
- 1H NMR spectra of compounds listed in Table 1 (**15**, **16**, **19**, **20**, **21**, **24**, **29**, **34**, **35**, **36**, **48**, **49**, **50**, **57**, **58**, **59**, **61**, **62** and **63)**. Pages 22-48

Table S1. Commercially available compounds tested as heparanase inhibitors. For each compound the reference inhibitor in the similarity search, the Tanimoto similarity index, the % of heparanase activity inhibition at the fixed concentrations of 25 and 2.5 μM or the IC_{50} values are reported.

Compd.	Molecular formula	Database[a]	Ref. compd.[b]	Similarity Index[c]	% inhib @25 μM ± SD	% inhib @2.5 μM ± SD	IC_{50} (μM) ± SD
**9**	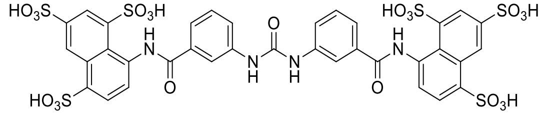	eMolecules	**5**	0.300	Not active		
**10**	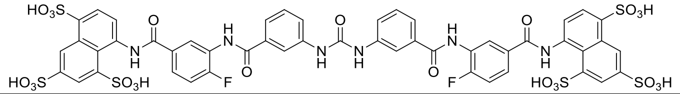	eMolecules	**5**	0.250	100 ± 1	39 ± 3	
**11**	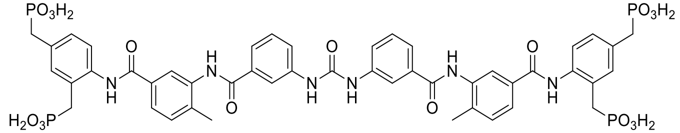	eMolecules	**5**	0.194	99 ± 2	Not active	
**12**	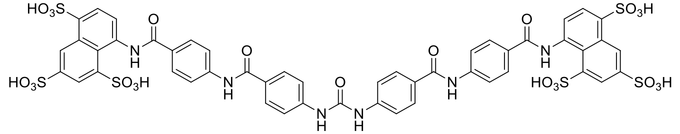	eMolecules	**5**	0.166	Not active		
**13**	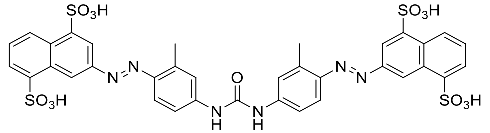	eMolecules	**5**	0.157	Not active		
**14**	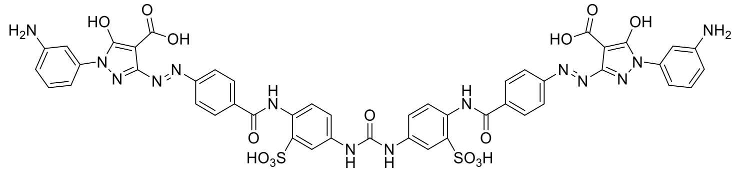	eMolecules	**5**	0.111	100 ± 1	36 ± 6	
**15**	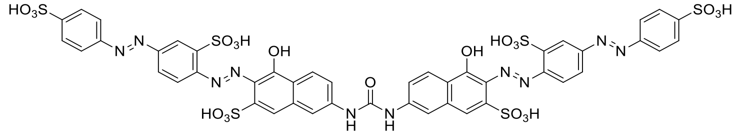	eMolecules	**5**	0.101			0.93 ± 0.07

Compd.	Molecular formula	Database[a]	Ref. compd.[b]	Similarity Index[c]	% inhib @25 μM ± SD	% inhib @2.5 μM ± SD	IC_{50} (μM) ± SD
**16**	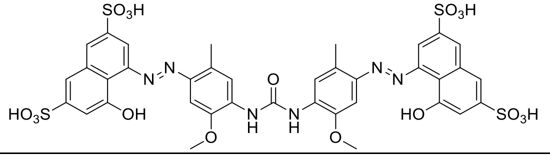	eMolecules	**5**	0.047			0.37 ± 0.01
**17**	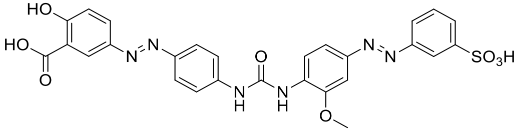	eMolecules	**16**	0.171	95 ± 1	14 ± 2	
**18**	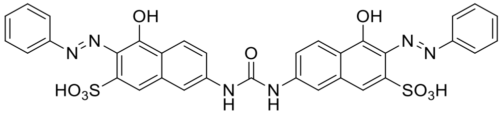	eMolecules	**16**	0.145	11 ± 3	12 ± 3	
**19**	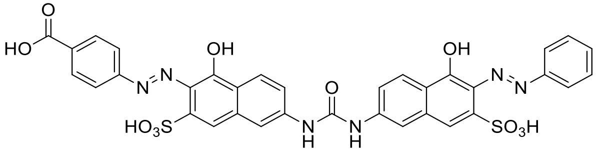	eMolecules	**16**	0.134			1.66 ± 0.12
**20**	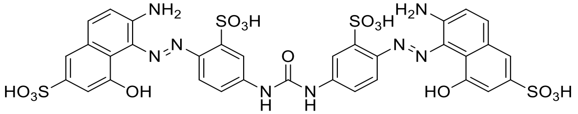	eMolecules	**16**	0.201			1.76 ± 0.23
**21**	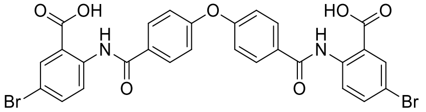	eMolecules	**6**	0.096			2.66 ± 032
**22**	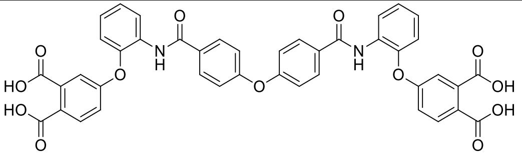	eMolecules	**6**	0.058	60 ±1	21 ± 1	
**23**	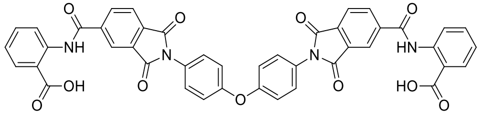	eMolecules	**6**	0.055	96 ± 3	88 ± 6	
**24**	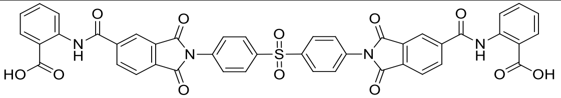	eMolecules	**6**	0.054			1.10 ± 0.13

Compd.	Molecular formula	Database[a]	Ref. compd.[b]	Similarity Index[c]	% inhib @25 μM ± SD	% inhib @2.5 μM ± SD	IC_{50} (μM) ± SD
**25**	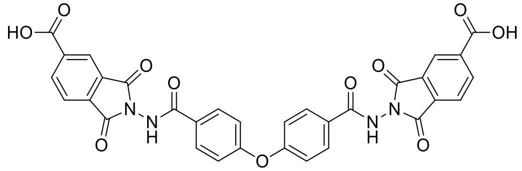	eMolecules	**6**	0.048	Not active		
**26**	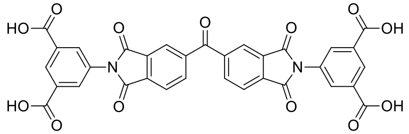	eMolecules	**6**	0.042	99 ± 6	35 ± 1	
**27**	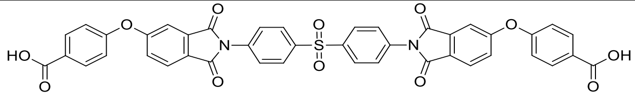	eMolecules	**6**	0.036	70 ± 6	Not active	
**28**	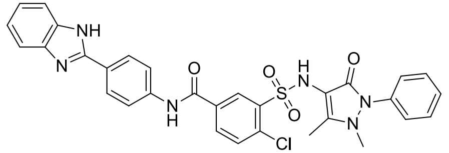	eMolecules	**7**	0.381	59 ± 5	14 ± 6	
**29**	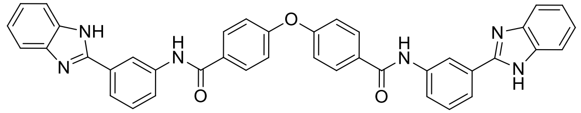	eMolecules	**7**	0.321			0.38 ± 0.04
**30**	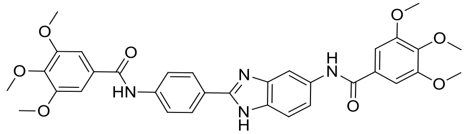	eMolecules	**7**	0.300	1 ± 1	11 ± 4	
**31**	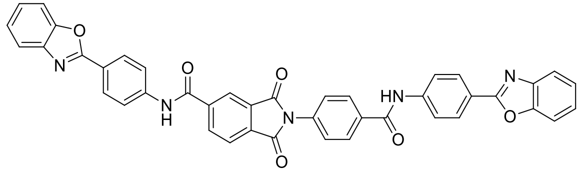	eMolecules	**7**	0.219	66 ± 13	43 ± 17	

Compd.	Molecular formula	Database[a]	Ref. compd.[b]	Similarity Index[c]	% inhib @25 μM ± SD	% inhib @2.5 μM ± SD	IC$_{50}$ (μM) ± SD
**32**	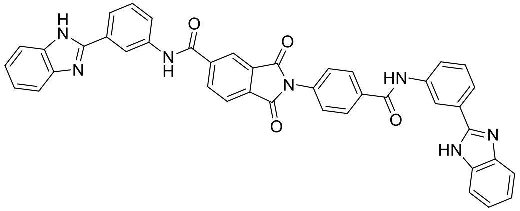	eMolecules	**7**	0.218	66 ± 1	20 ± 1	
**33**	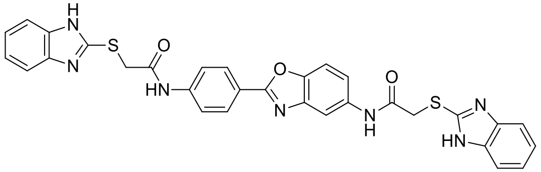	eMolecules	**7**	0.214	58 ± 1	36 ± 5	
**34**	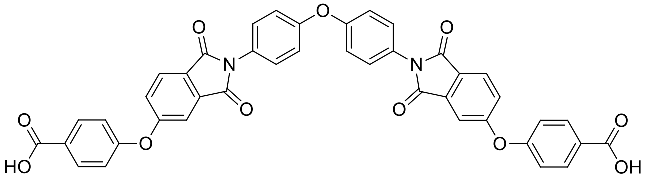	eMolecules	**29**	0.088			3.15 ± 0.28
**35**	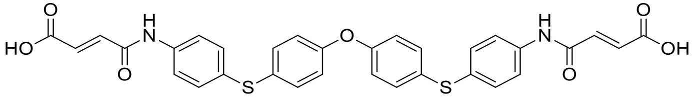	eMolecules	**29**	0.089			0.52 ± 0.02
**36**	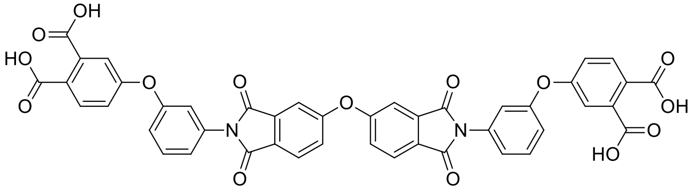	eMolecules	**29**	0.060			0.63 ± 0.01
**37**	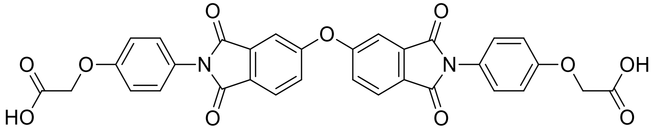	eMolecules	**29**	0.066			> 5
**38**	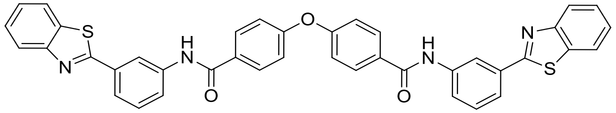	eMolecules	**29**	0.430			5.75 ± 0.05

Compd.	Molecular formula	Database[a]	Ref. compd.[b]	Similarity Index[c]	% inhib @25 μM ± SD	% inhib @2.5 μM ± SD	IC$_{50}$ (μM) ± SD
**39**	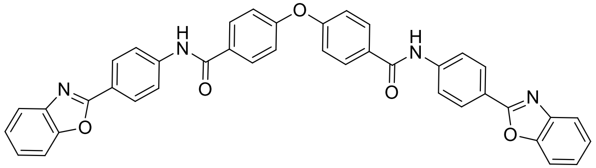	eMolecules	**29**	0.239	63 ± 16	60 ± 21	
**40**	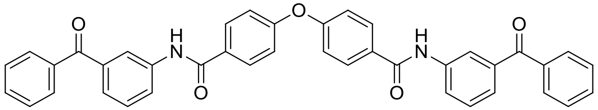	eMolecules	**29**	0.309			> 5
**41**	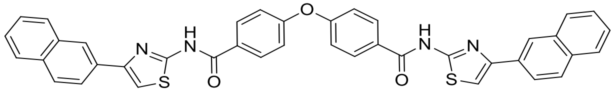	eMolecules	**29**	0.159	76 ± 2	54 ± 6	
**42**	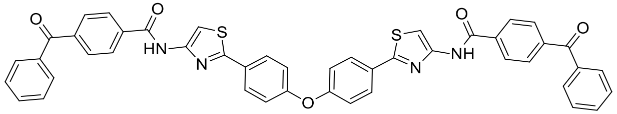	eMolecules	**29**	0.148	55 ± 6	49 ± 8	
**43**	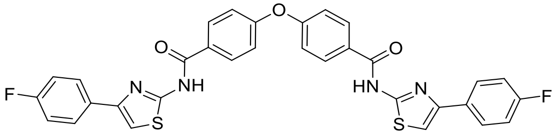	eMolecules	**29**	0.146	67 ± 4	44 ± 1	
**44**	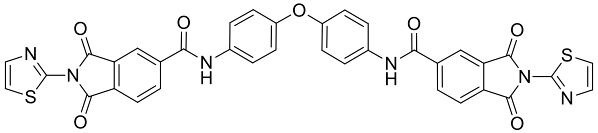	eMolecules	**29**	0.098	60 ± 5	62 ± 2	
**45**	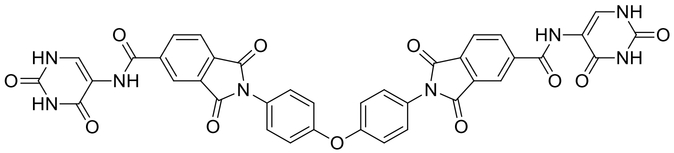	eMolecules	**29**	0.085	67 ± 4	41 ± 3	
**46**	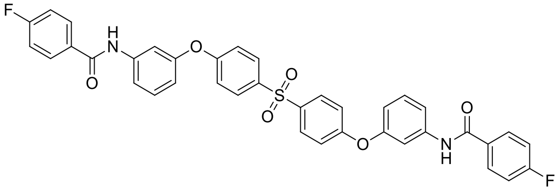	eMolecules	**35**	0.131			> 5

Compd.	Molecular formula	Database[a]	Ref. compd.[b]	Similarity Index[c]	% inhib @25 μM ± SD	% inhib @2.5 μM ± SD	IC_{50} (μM) ± SD
**47**	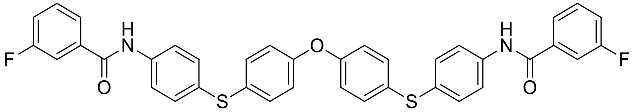	eMolecules	**35**	0.426	66 ± 5	30 ± 3	
**48**	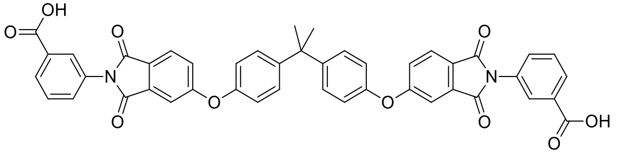	eMolecules	**35**	0.065			0.91 ± 0.10
**49**	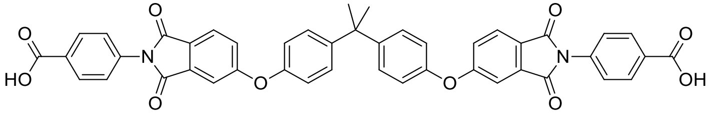	eMolecules	**35**	0.073			2.35 ± 0.23
**50**	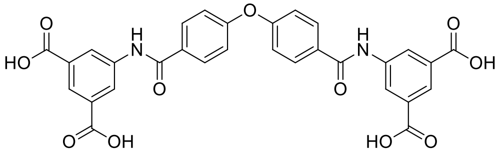	SciFinder	**29**	0212			2.01 ± 0.13
**51**	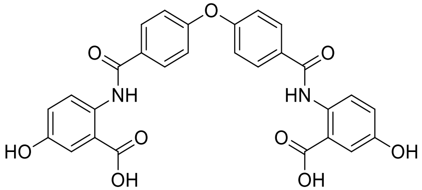	SciFinder	**29**	0.176	77 ± 4	42 ± 6	
**52**	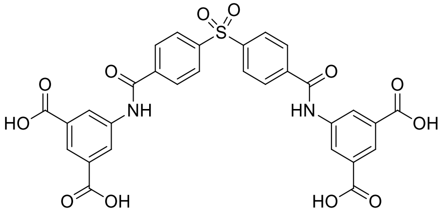	SciFinder	**29**	0.130	54 ± 8	45 ± 7	
**53**	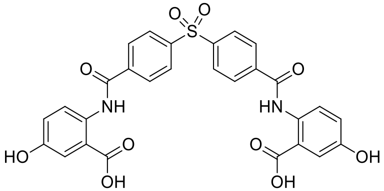	SciFinder	**29**	0.106			5.29 ± 0.84

Compd.	Molecular formula	Database[a]	Ref. compd.[b]	Similarity Index[c]	% inhib @25 μM ± SD	% inhib @2.5 μM ± SD	IC_{50} (μM) ± SD
**54**	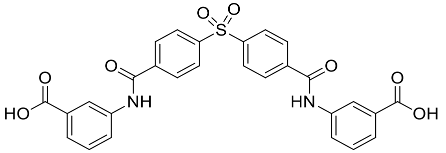	SciFinder	**29**	0.233	72 ± 1	22 ± 7	
**55**	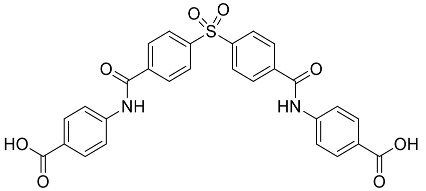	SciFinder	**29**	0.105	43 ± 7	30 ± 4	
**56**	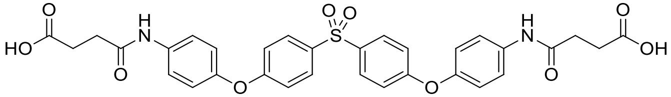	SciFinder	**35**	0.262	51 ± 5	20 ± 5	
**57**	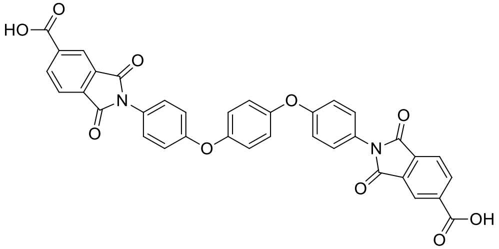	SciFinder	**35**	0.092			2.17 ± 0.18
**58**	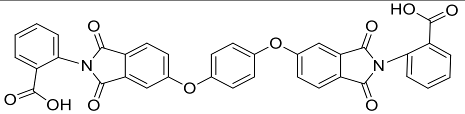	SciFinder	**35**	0.066			3.78 ± 0.22
**59**	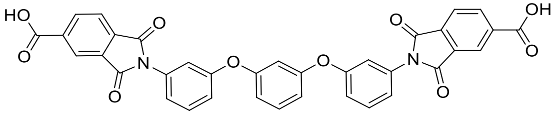	SciFinder	**57**	0.055			2.08 ± 0.16
**60**	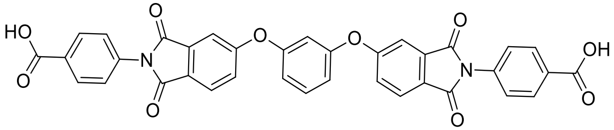	SciFinder	**57**	0.076	65 ± 4	53 ± 12	

Compd.	Molecular formula	Database[a]	Ref. compd.[b]	Similarity Index[c]	% inhib @25 μM ± SD	% inhib @2.5 μM ± SD	IC_{50} (μM) ± SD
**61**	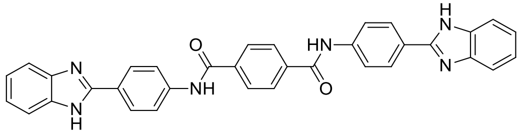	SciFinder	**57**	0.398			0.32 ± 0.06
**62**	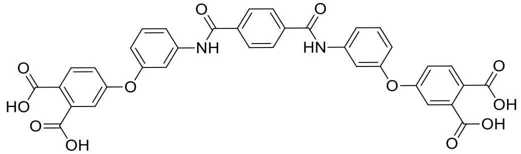	SciFinder	**57**	0.208			3.13 ± 0.27
**63**	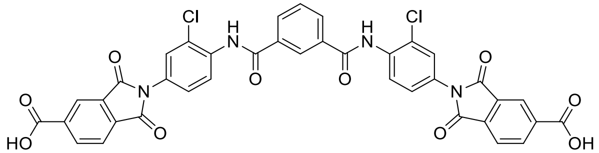	SciFinder	**57**	0.069			0.12 ± 0.01

[a]Database from which the compound was selected. [b]Reference heparanase inhibitor (see formulas in Figure 2 and Table 1 of the main text) used in similarity search which led to the selection of the compound. [c]Tanimoto similarity with the reference inhibitor (indicated in the preceding column). Similarity index was calculated for the most abundant species at physiological pH, determined with EpiK software.[1]

[1]Shelley J.C., Cholleti A., Frye L.L. *et al*. Epik: a software program for pK(a) prediction and protonation state generation for drug-like molecules. *J. Comput.-Aided Mol. Des*. **2007**, *21*, 681-691.

Figure S1. Dose-response curves obtained for compounds reported in Table 1.

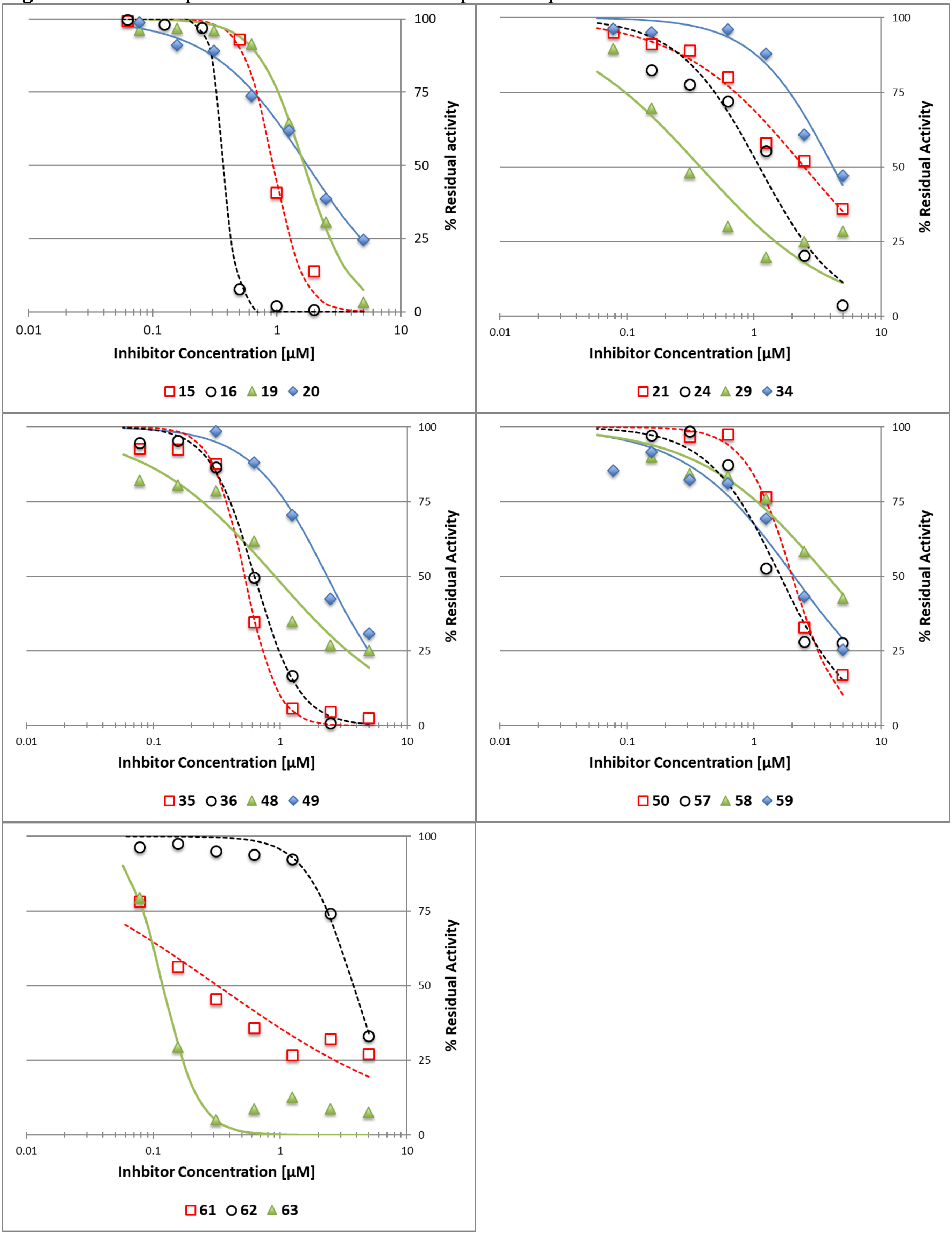

Figure S2. Best docking poses obtained for compounds **29** (yellow carbons) and **35** (light blue carbons) into human heparanase.

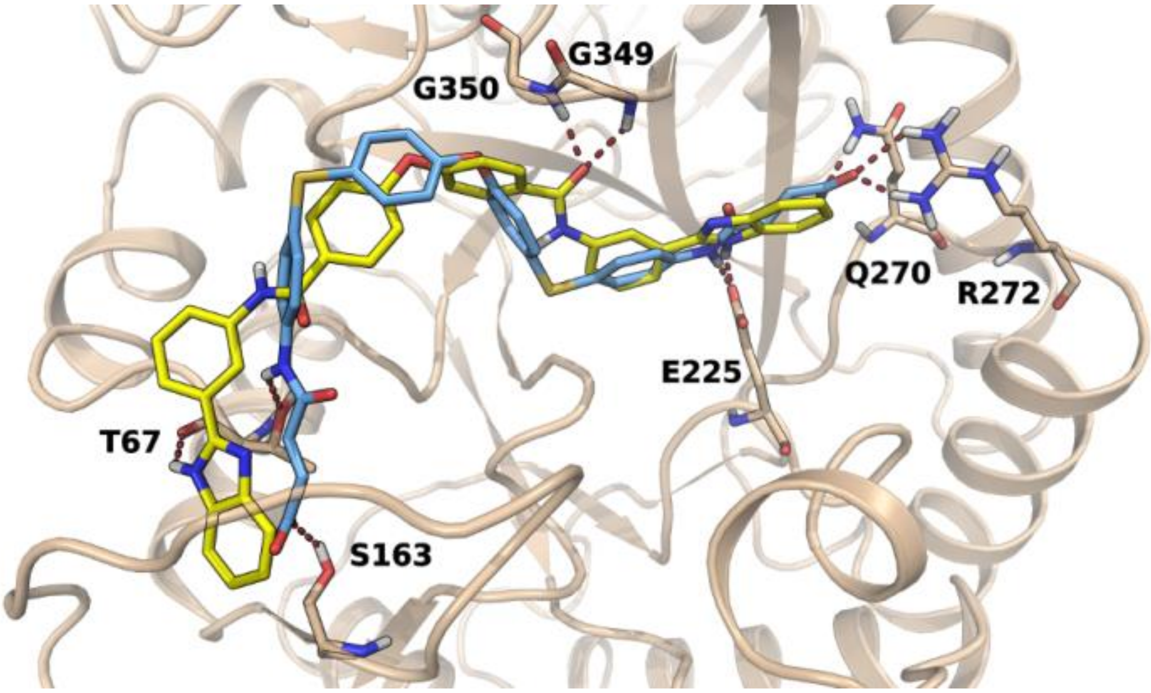

Table S2. CAS number, molecular weight, vendor and purity of tested compounds.

Compd.	CAS NUMBER	Molecular weight	Vendor	Purity (%)[a]
**9**	104869-31-0 (sodium salt)	1030.99 (free base) 1162.88 (6 Na^+)	Santa Cruz	≥ 98
**10**	104910-21-6 (sodium salt)	1305.21 (free base) 1671.35 (6 Na^+, 13 H_2O)	TOCRIS	> 95
**11**	1006028-37-0 (sodium salt)	1092.81 (free base) 1396.95 (4 Na^+, 12 H_2O)	TOCRIS	96
**12**	202983-32-2 (sodium salt)	1269.23 (free base) 1401.18 (6 Na^+)	Santa Cruz	> 95
**13**	3214-47-9 (sodium salt)	868.89 (free base) 956.86 (4 Na^+)	Aldrich	NA
**14**	1265627-53-9 (sodium salt)	1101.00 (free base) 1188.98 (4 Na^+)	Aldrich	NA
**15**	2610-10-8 (sodium salt)	1241.18 (free base) 1373.13 (6 Na^+)	Aldrich	≥ 95[c]
**16**	1937-34-4 (sodium salt)	960.94 (free base) 1048.87 (4 Na^+)	Santa Cruz	95.1[b]
**17**	7248-45-5 (sodium salt)	590.56 (free base) 634.55 (2 Na^+)	AK Scientific	> 98
**18**	3626-36-6 (sodium salt)	712.71 (free base) 756.70 (2 Na^+)	MP-Biomedicals LLC	> 98
**19**	6598-63-6 (sodium salt)	756.72 (free base) 822.70 (3 Na^+)	AK Scientific	> 98
**20**	2829-43-8 (sodium salt)	902.86 (free base) 990.79 (4 Na^+)	Santa Cruz	98
**21**	330634-98-5	654.26	Vitas-M Laboratory	> 98
**22**	328011-93-4	768.68	Vitas-M Laboratory	> 90
**23**	294666-29-8	786.70	Vitas-M Laboratory	> 90
**24**	352644-81-6	834.76	Ambinter	> 90
**25**	306320-21-8	634.51	Vitas-M Laboratory	> 95
**26**	294667-55-3	648.49	Vitas-M Laboratory	> 90
**27**	148878-08-4	780.71	Vitas-M Laboratory	> 90
**28**	378191-68-5	613.09	Uorsy	> 98
**29**	477493-81-5	640.69	Life Chemicals	95.6[b]
**30**	251341-72-7	612.63	Vitas-M Laboratory	> 98
**31**	311797-65-6	695.69	Vitas-M Laboratory	> 98
**32**	303138-91-2	693.71	Vitas-M Laboratory	> 98
**33**	312281-77-9	605.69	Vitas-M Laboratory	> 98
**34**	148878-10-8	732.65	Ambinter	> 90
**35**	351188-09-5	612.67	Vitas-M Laboratory	> 95
**36**	351521-52-3	820.67	Ambinter	> 90
**37**	370871-91-3	608.51	Ambinter	> 90
**38**	476634-38-5	674.79	Aurora	> 95
**39**	351893-10-2	642.66	Vitas-M Laboratory	> 95
**40**	351188-37-9	616.66	Aurora	> 95
**41**	476296-13-6	674.79	Aurora	> 95
**42**	397288-95-8	782.78	Aurora	> 95
**43**	476296-10-3	610.65	Ambinter	> 95

**Compd.**	**CAS NUMBER**	**Molecular weight**	**Vendor**	**Purity (%)**[a]
**44**	295348-44-6	712.71	Ambinter	> 95
**45**	350709-22-7	766.63	Ambinter	> 90
**46**	330635-99-9	676.68	Vitas-M Laboratory	> 95
**47**	294891-04-6	660.75	Ambinter	< 90
**48**	226415-59-4	758.73	Ambinter	> 90
**49**	134118-32-4	758.73	Ambinter	> 90
**50**	304885-58-3	584.49	Vitas-M Laboratory	> 95
**51**	327996-87-2	528.47	Vitas-M Laboratory	> 95
**52**	332403-67-5	632.55	Vitas-M Laboratory	> 95
**53**	609821-77-4	576.53	Ambinter	> 95
**54**	609821-66-1	544.53	Ambinter	> 95
**55**	609821-65-0	544.53	Ambinter	> 95
**56**	331972-86-2	632.64	Ambinter	> 90
**57**	130651-50-2	640.55	Vitas-M Laboratory	> 95
**58**	302926-71-2	640.55	Ambinter	> 90
**59**	401603-58-5	640.55	Ambinter	> 90
**60**	330816-92-7	640.55	Ambinter	> 90
**61**	191660-01-2	548.59	Ambinter	95.6[b]
**62**	330971-85-2	676.58	Vitas-M Laboratory	> 95
**63**	340183-01-9	763.49	Ambinter	95.0[b]

[a]Purity percentage declared by vendor unless otherwise specified. [b]Experimentally determined (see below for experimental procedures and results). [c]Estimated from ^{1}H NMR spectrum. NA: not available.

HPLC-UV traces and ESI mass spectra of target compounds 16, 29, 61 and 63.

Compound 16

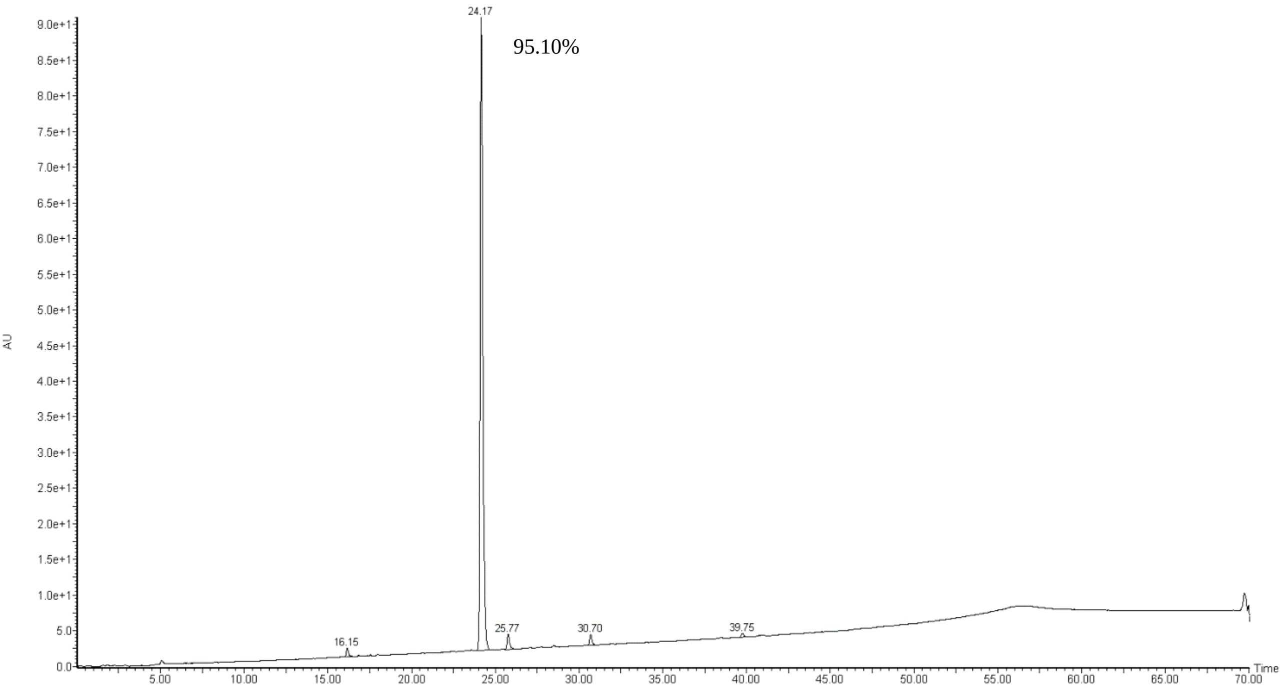

Column: Phenomenex Gemini C18 150*4.6 mm, 5 μm
Detector: UV PDA (λ range 210 - 800 nm)
Mobile Phase A: Water/MeOH=95/5 10 mM Ammonium Acetate
Mobile Phase B: Acetonitrile/Methanol=50/50 25 mM Ammonium Acetate

Time (min)	A%	B%	Flow (mL/min)	Curve
0.00	100.0	0.0	1.000	1
1.00	100.0	0.0	1.000	6
53.00	0.0	100.0	1.000	6
67.00	0.0	100.0	1.000	6
68.00	100.0	0.0	1.000	6
70.00	100.0	0.0	1.000	6

Compound 16

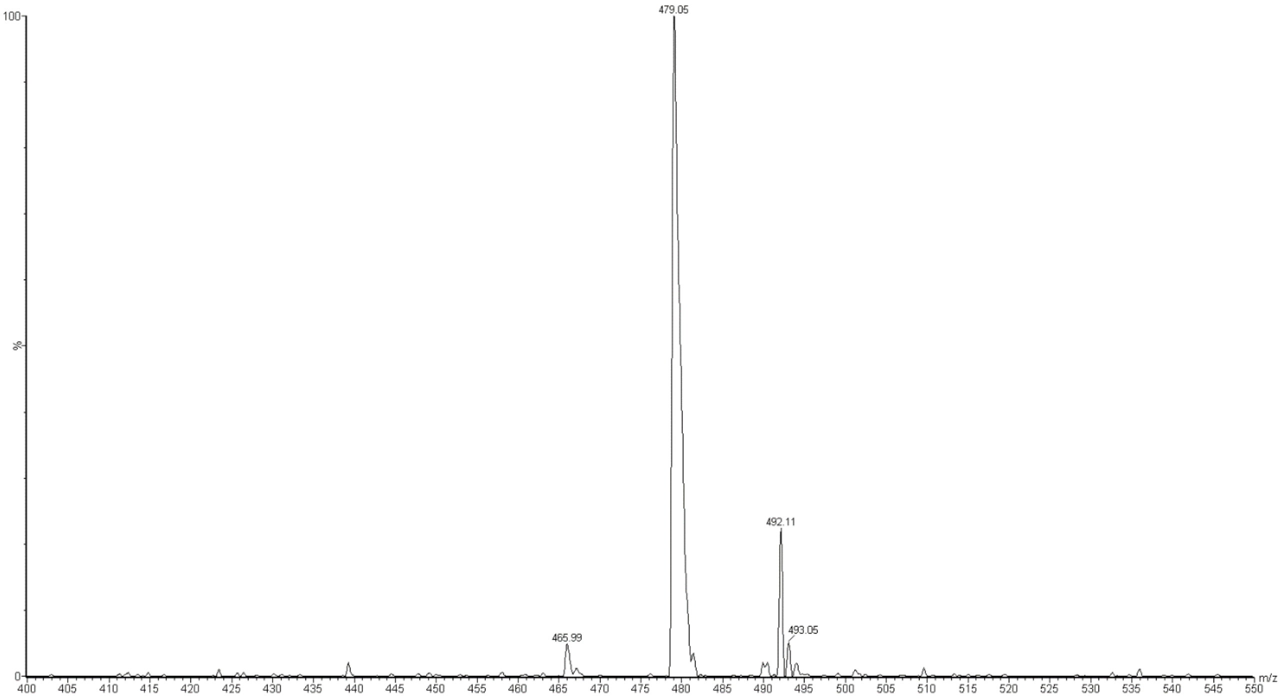

ESI$^-$ Mass spectrum at RT = 24.17 min 479.05 = $[M-2H]^{2-}/2$

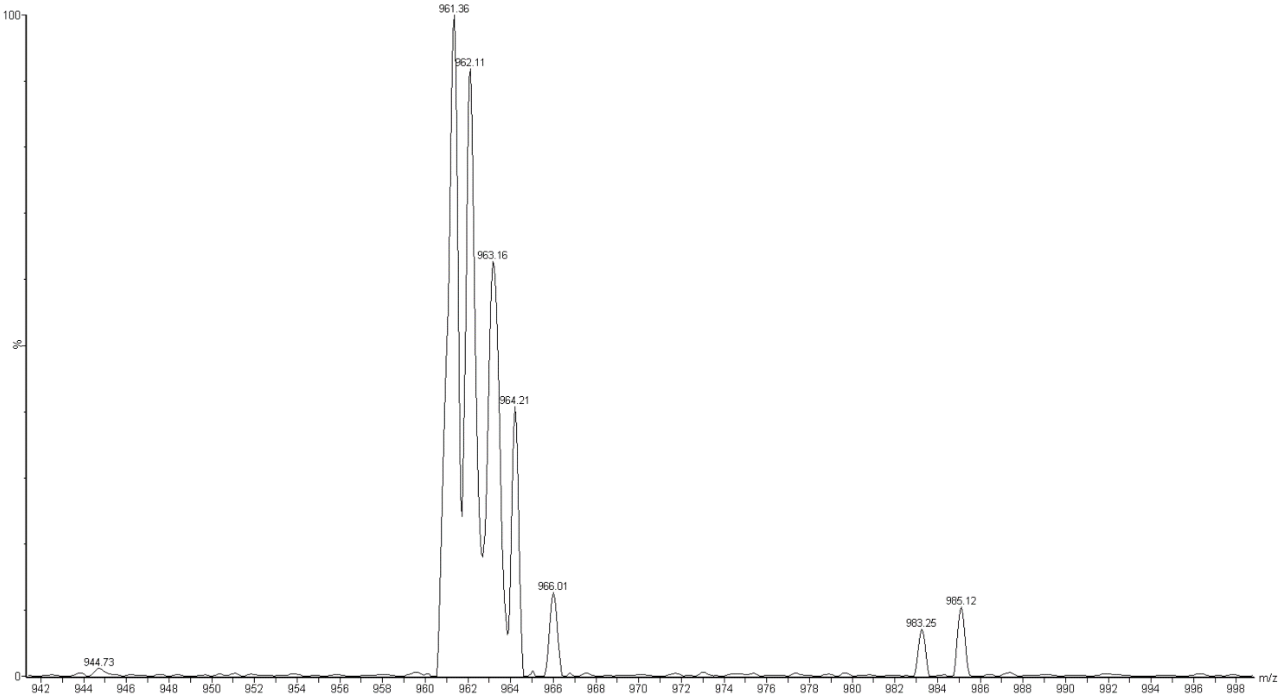

ESI$^+$ Mass spectrum at RT = 24.17 min 961.36 = $[M+H]^+$

Compound 29

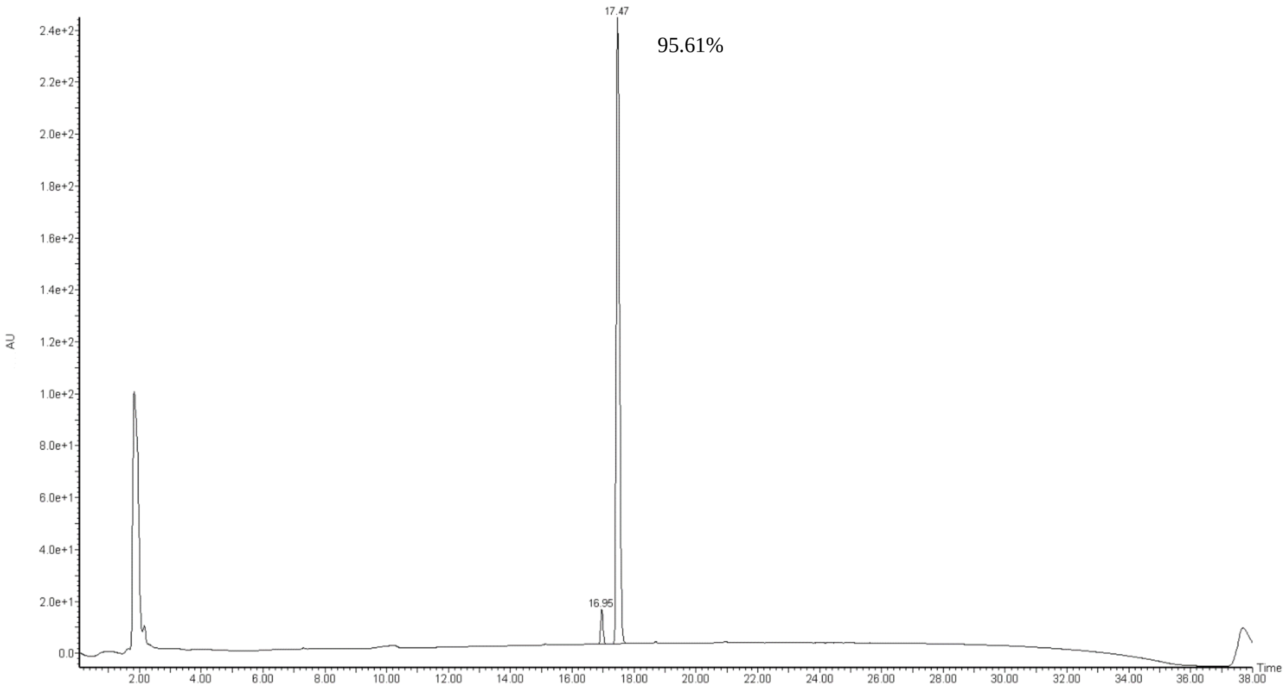

Column: Waters XBridge Phenyl 150*4.6 mm, 3.5 μm
Detector: UV PDA (λ range 190 – 600 nm)
Mobile Phase A: Water 0.1% trifluoroacetic acid
Mobile Phase B: Acetonitrile 0.1% trifluoroacetic acid

Time (min)	A%	B%	Flow (mL/min)	Curve
0.00	90.0	10.0	1.000	1
3.00	90.0	10.0	1.000	6
33.00	10.0	90.0	1.000	6
35.00	10.0	90.0	1.000	6
36.00	90.0	10.0	1.000	6
38.00	90.0	10.0	1.000	6

Compound 29

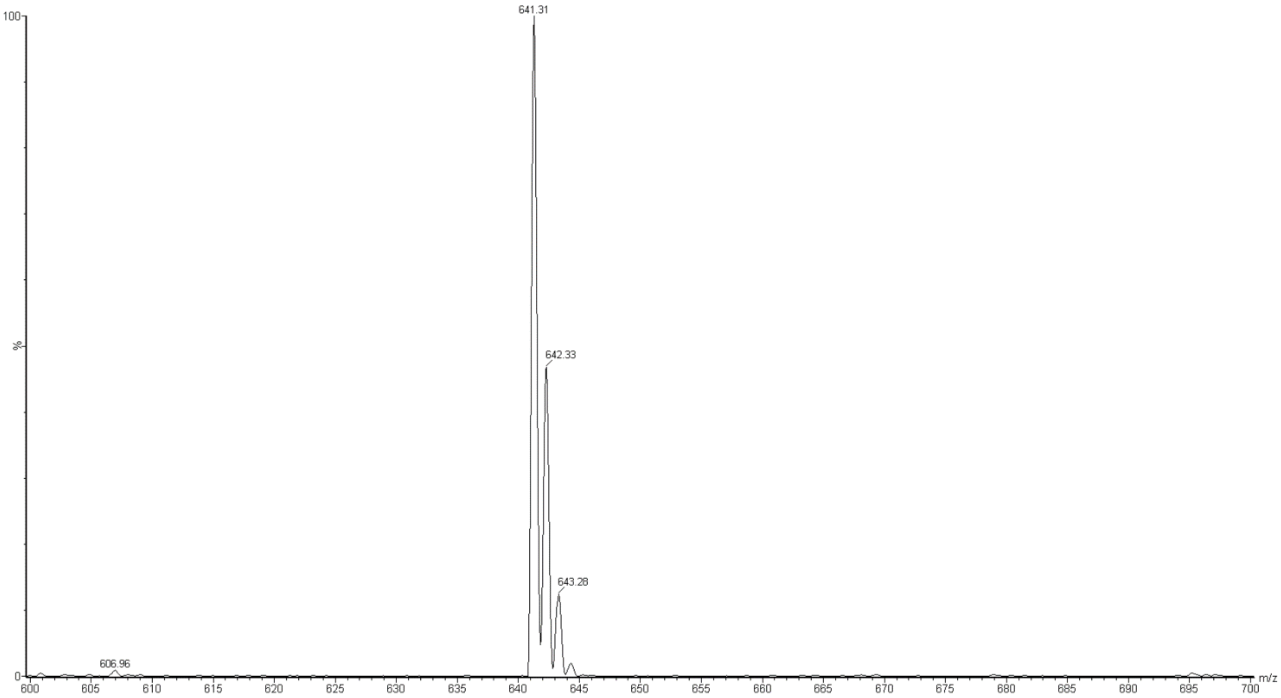

ESI$^+$ Mass spectrum at RT = 17.47 min 641.31 = $[M+H]^+$

Compound 61

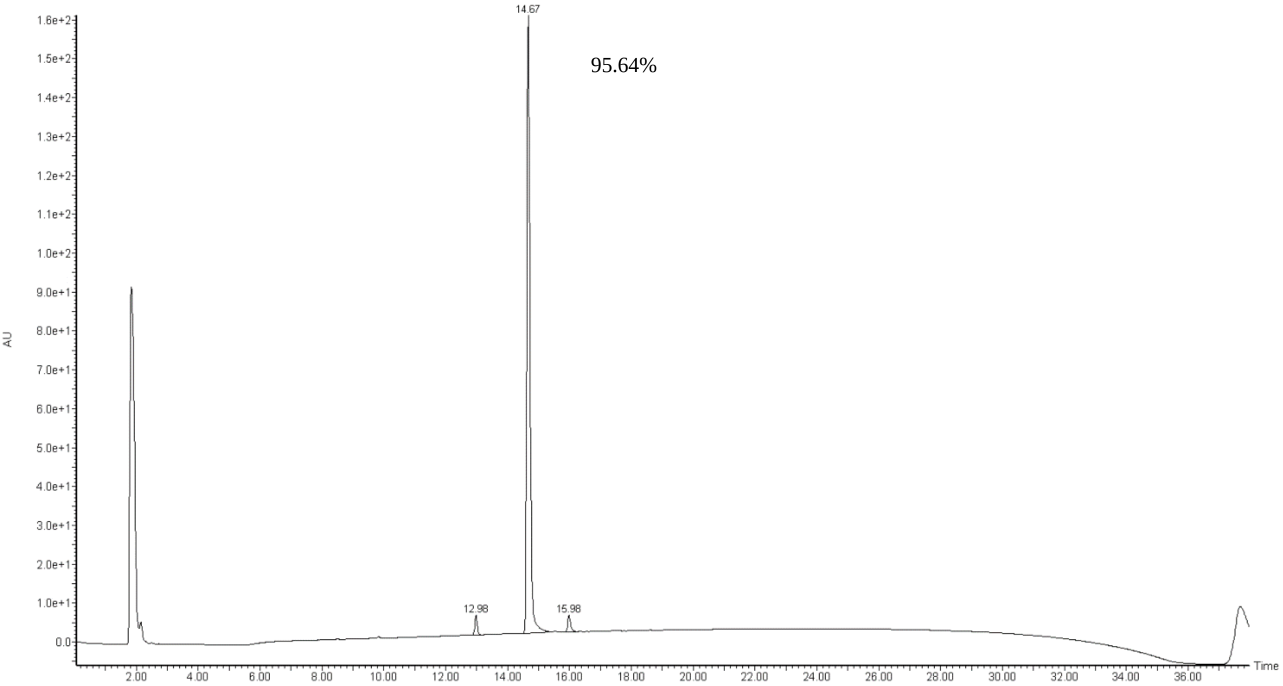

Column: Waters XBridge Phenyl 150*4.6 mm, 3.5 μm
Detector: UV PDA (λ range 190 – 600 nm)
Mobile Phase A: Water 0.1% trifluoroacetic acid
Mobile Phase B: Acetonitrile 0.1% trifluoroacetic acid

Time (min)	A%	B%	Flow (mL/min)	Curve
0.00	90.0	10.0	1.000	1
3.00	90.0	10.0	1.000	6
33.00	10.0	90.0	1.000	6
35.00	10.0	90.0	1.000	6
36.00	90.0	10.0	1.000	6
38.00	90.0	10.0	1.000	6

Compound 61

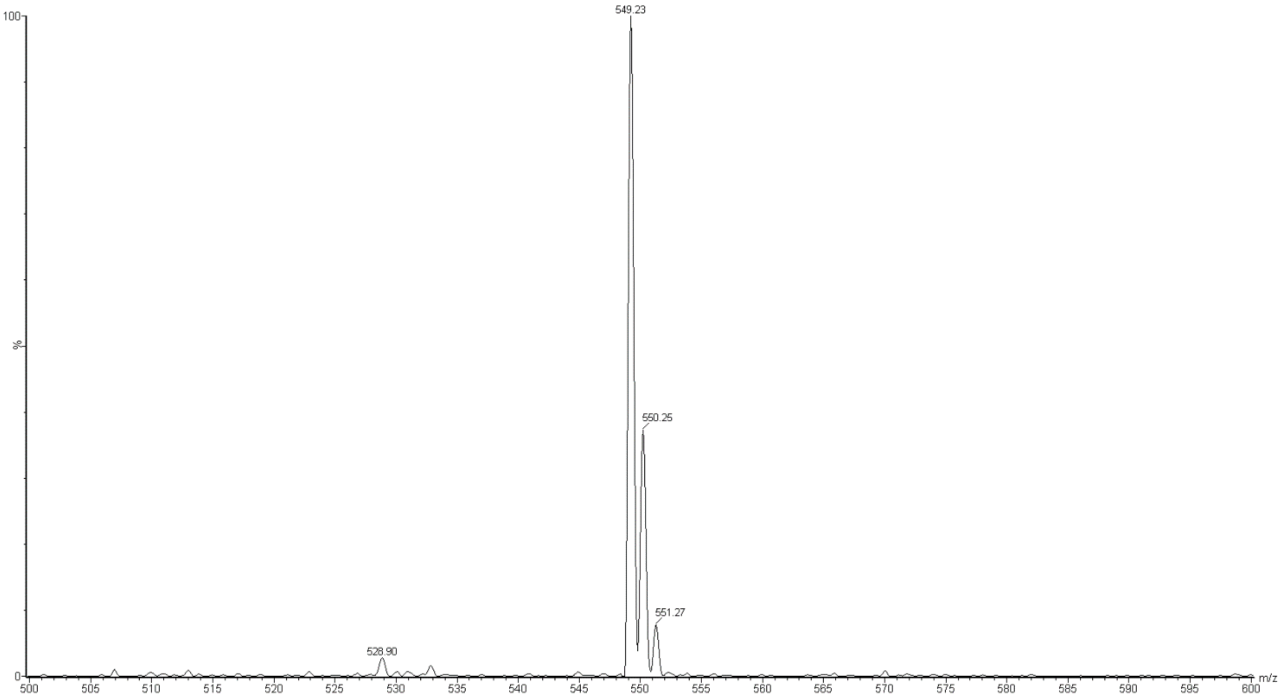

ESI$^+$ Mass spectrum at RT = 14.67 min 549.23 = $[M+H]^+$

Compound 63

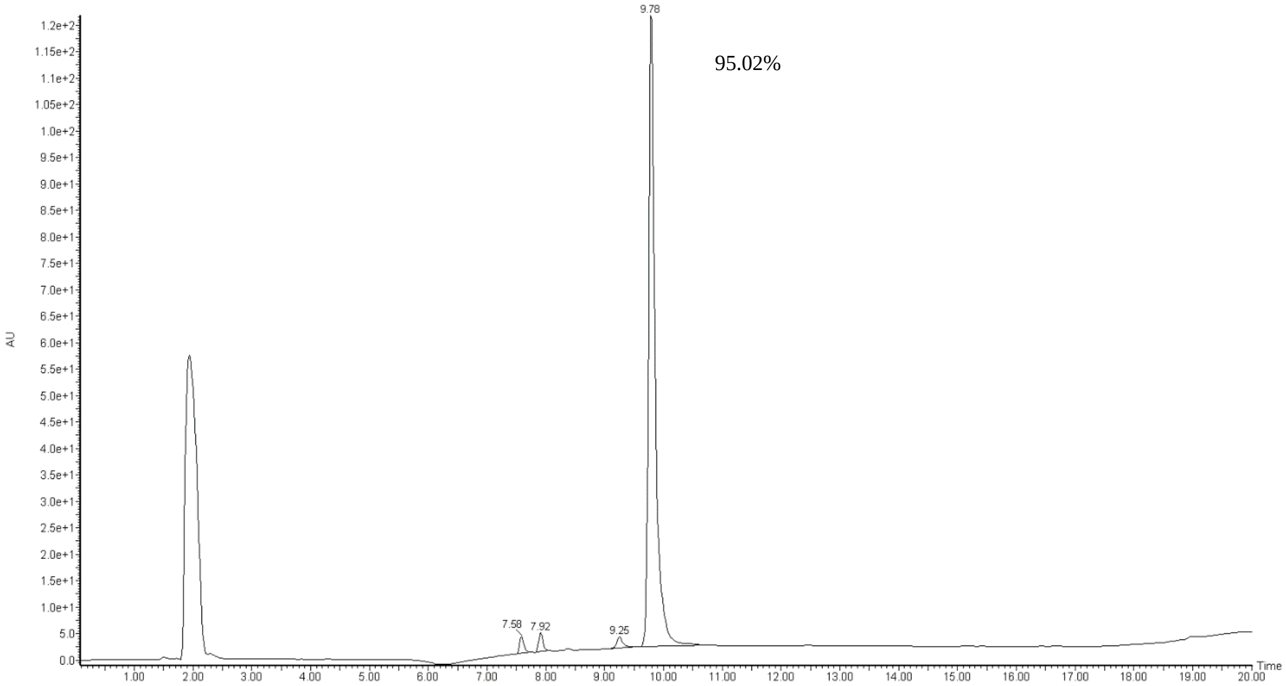

Column: Waters XBridge Phenyl-hexyl 150*4.6 mm, 3 μm
Detector: UV PDA (λ range 210 - 800 nm)
Mobile Phase A: Water/MeOH=95/5 10 mM Ammonium Acetate
Mobile Phase B: Acetonitrile/Methanol=50/50 25 mM Ammonium Acetate

Time (min)	A%	B%	Flow (mL/min)	Curve
0.00	90.0	10.0	1.000	1
3.00	90.0	10.0	1.000	1
15.00	45.0	55.0	1.000	3
17.00	90.0	10.0	1.000	6
20.00	90.0	10.0	1.000	6

Compound 63

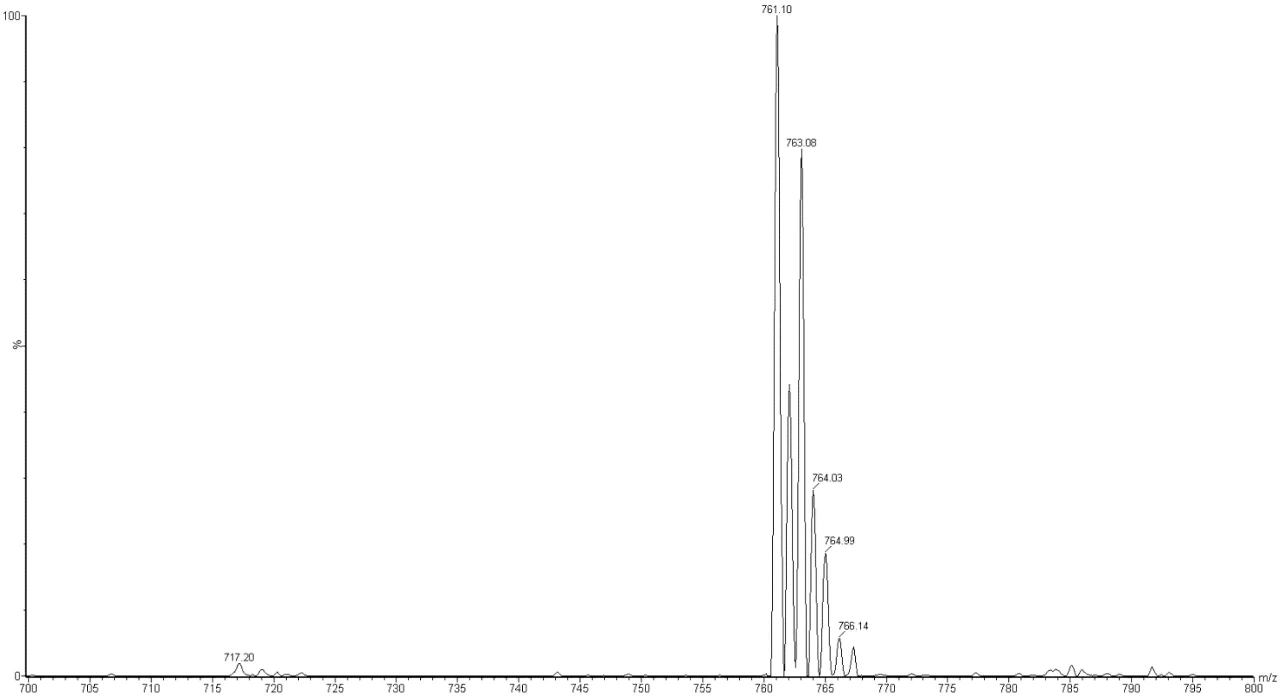

ESI⁻ Mass spectrum at RT = 9.78 min 761.10 = $[M-H]^-$

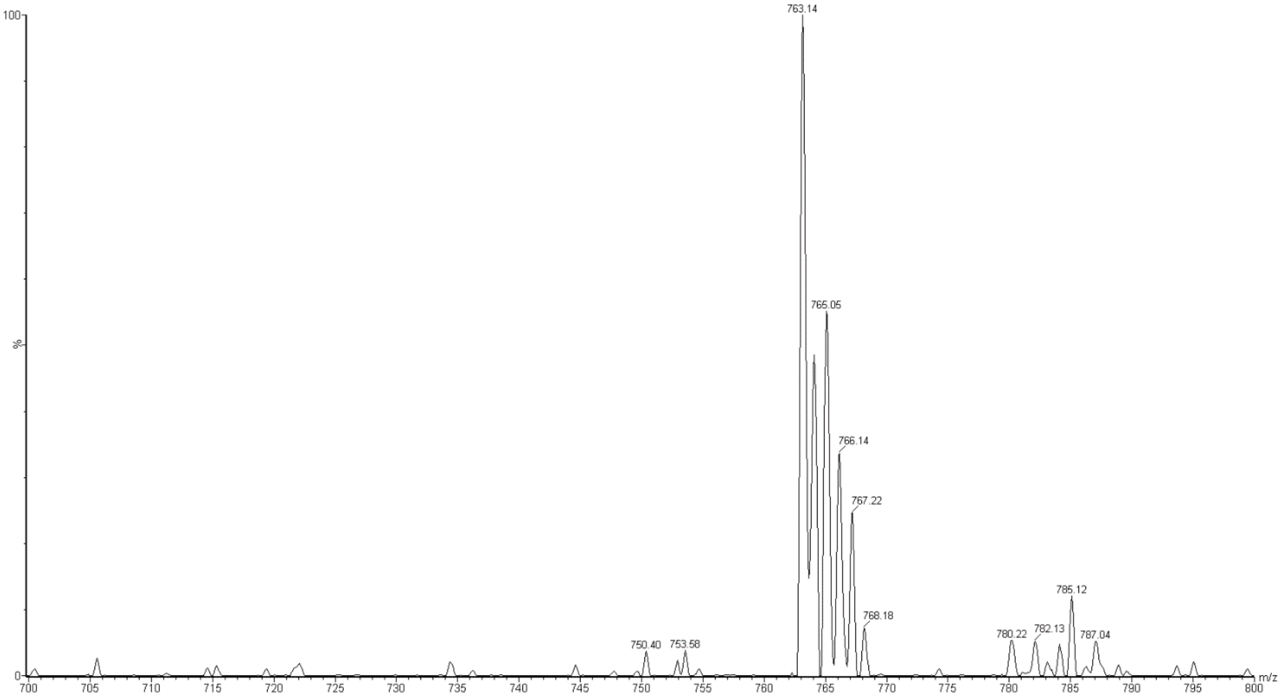

ESI$^+$ Mass spectrum at RT = 9.78 min 763.14 = $[M+H]^+$

Compound **15**

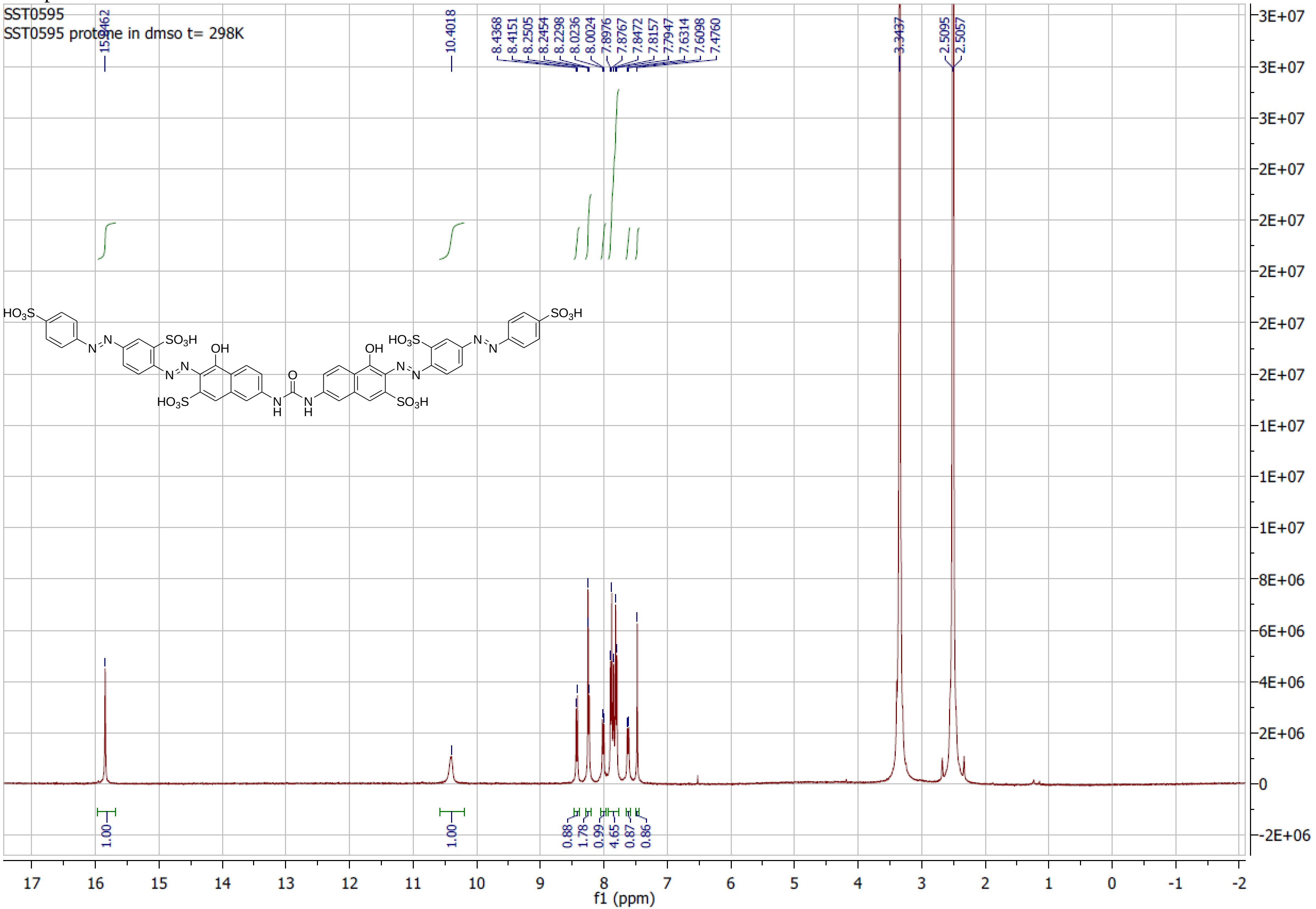
SST0595
SST0595 protone in dmso t= 298K
15.9462
10.4018
8.4368
8.4151
8.2505
8.2454
8.2298
8.0236
8.0024
7.8976
7.8767
7.8472
7.8157
7.7947
7.6314
7.6098
7.4760
3.3437
2.5095
2.5057
1.00
1.00
0.88
1.78
0.99
4.65
0.87
0.86
f1 (ppm)
HO3S
SO3H
OH
O
N
H

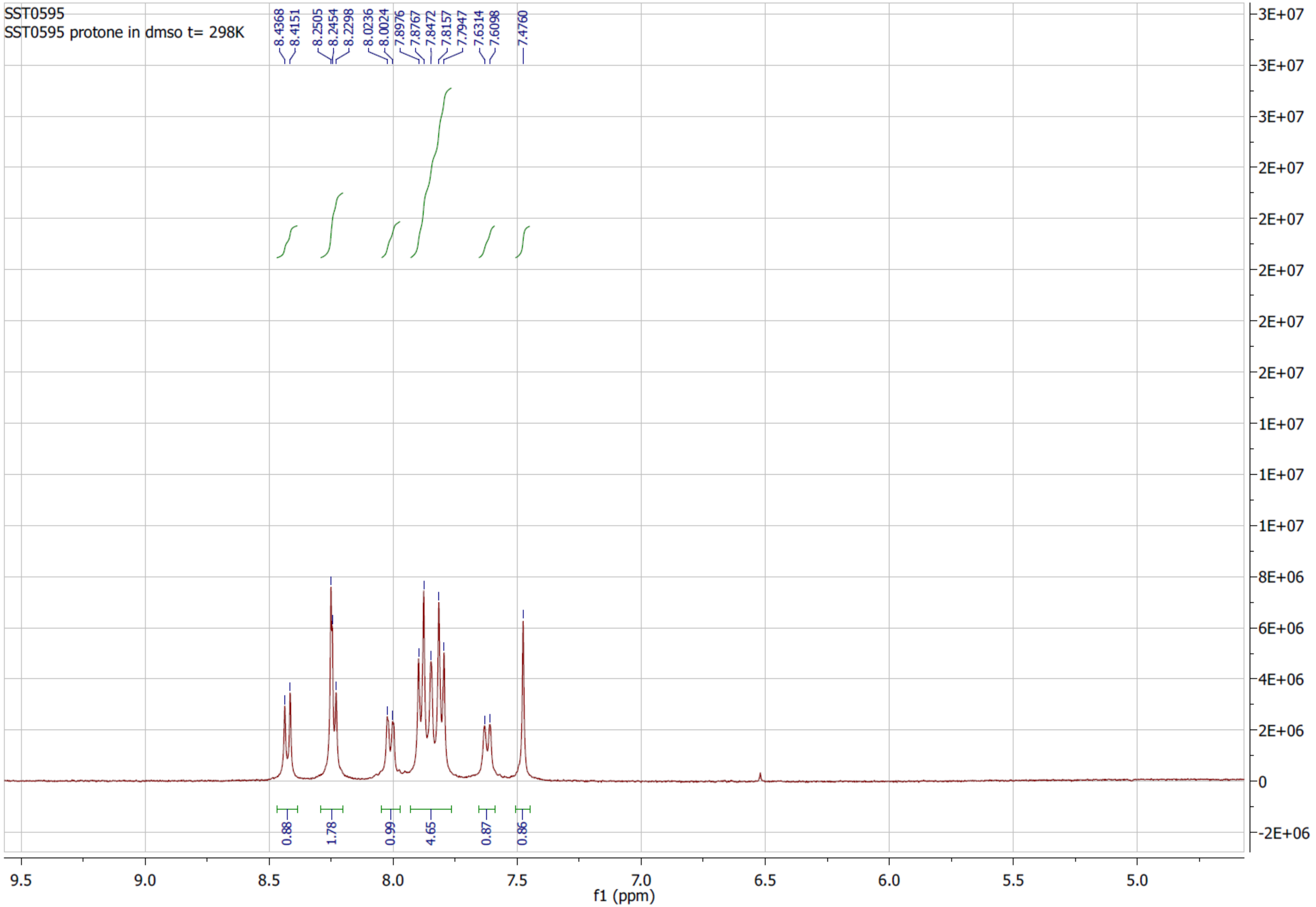
SST0595
SST0595 protone in dmso t= 298K
8.4368
8.4151
8.2505
8.2454
8.2298
8.0236
8.0024
7.8976
7.8767
7.8472
7.8157
7.7947
7.6314
7.6098
7.4760
0.88
1.78
0.99
4.65
0.87
0.86
f1 (ppm)

Compound **16**

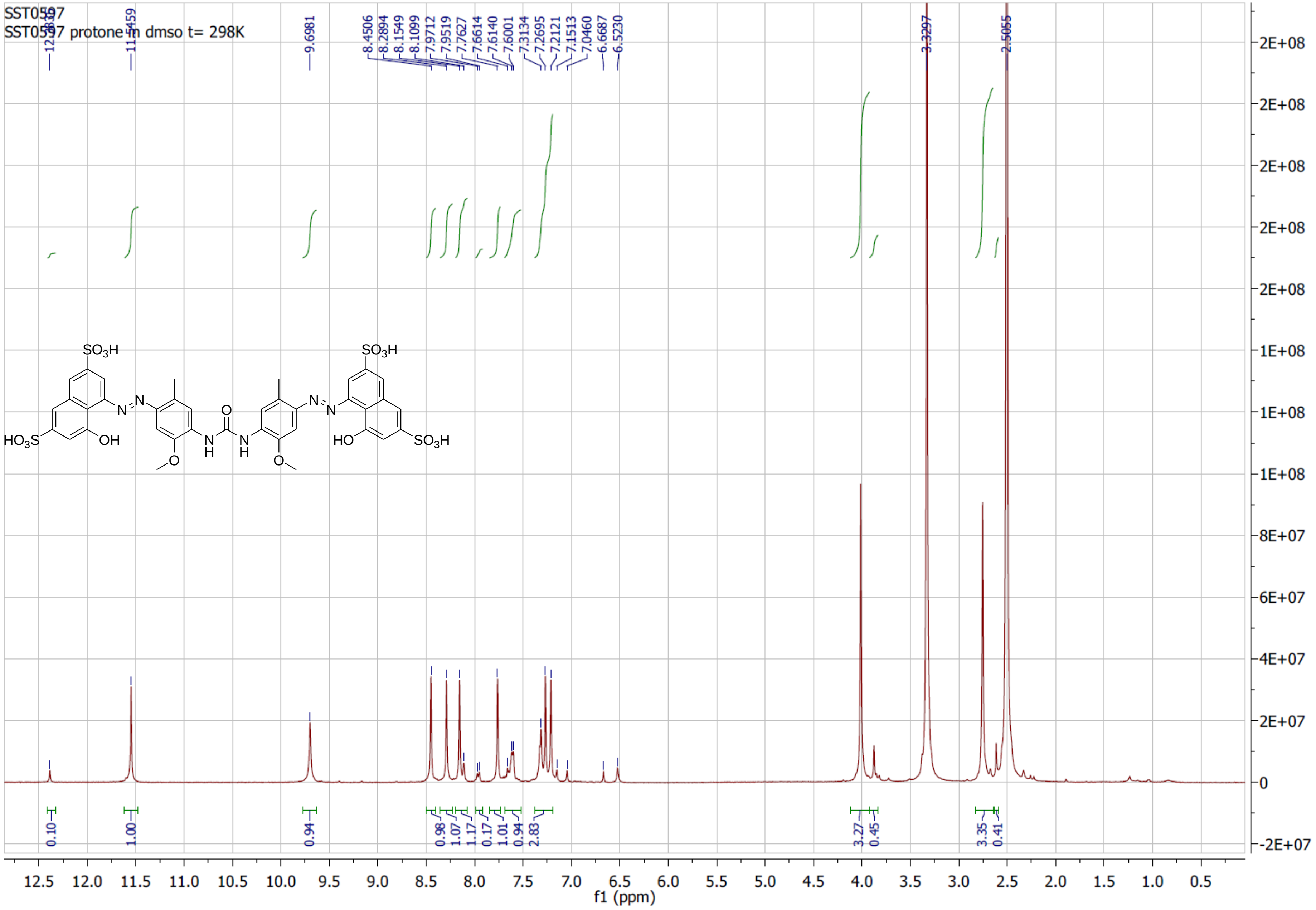

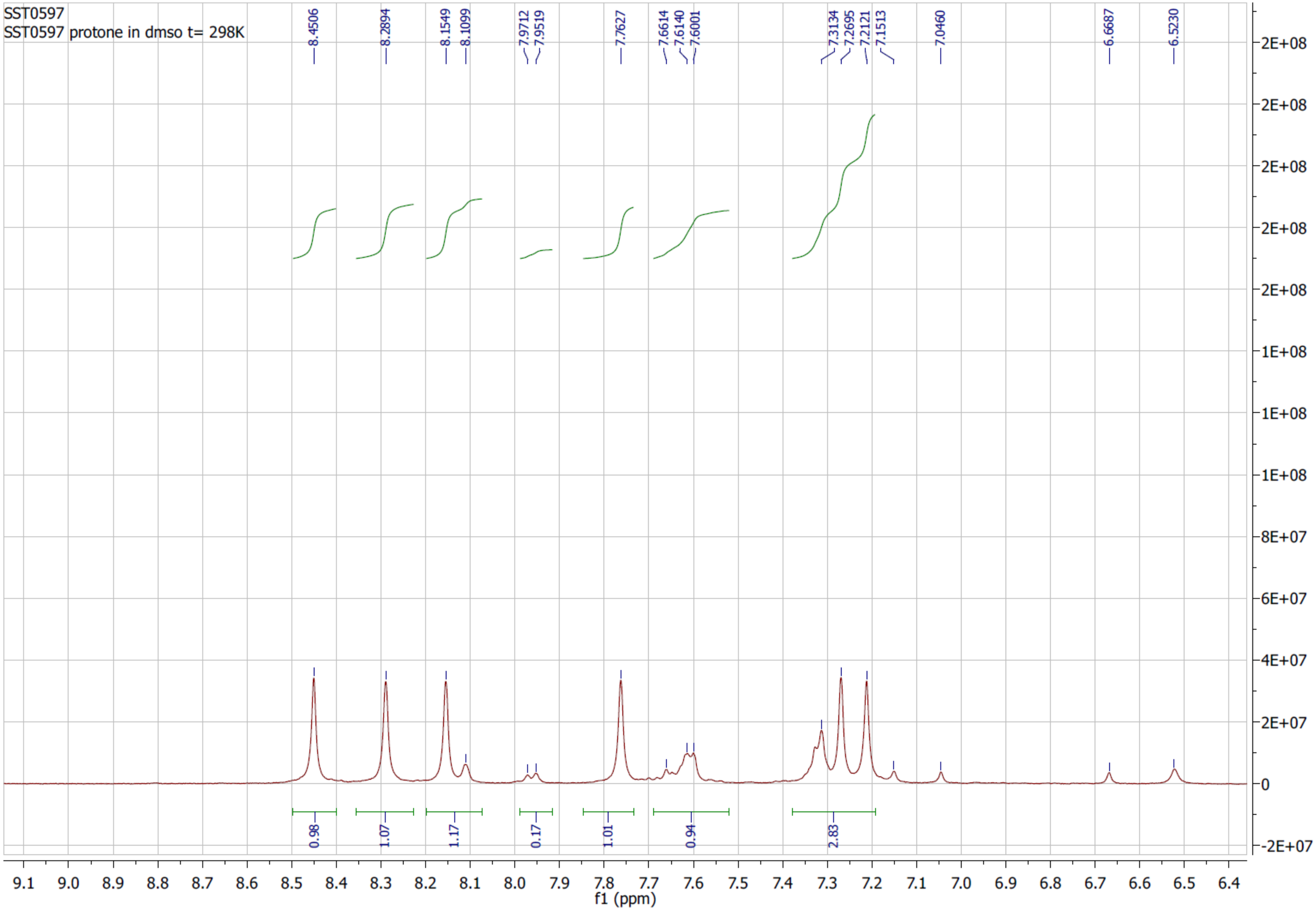
SST0597
SST0597 protone in dmso t= 298K
8.4506
8.2894
8.1549
8.1099
7.9712
7.9519
7.7627
7.6614
7.6140
7.6001
7.3134
7.2695
7.2121
7.1513
7.0460
6.6687
6.5230
0.98
1.07
1.17
0.17
1.01
0.94
2.83
f1 (ppm)

Compound **19**

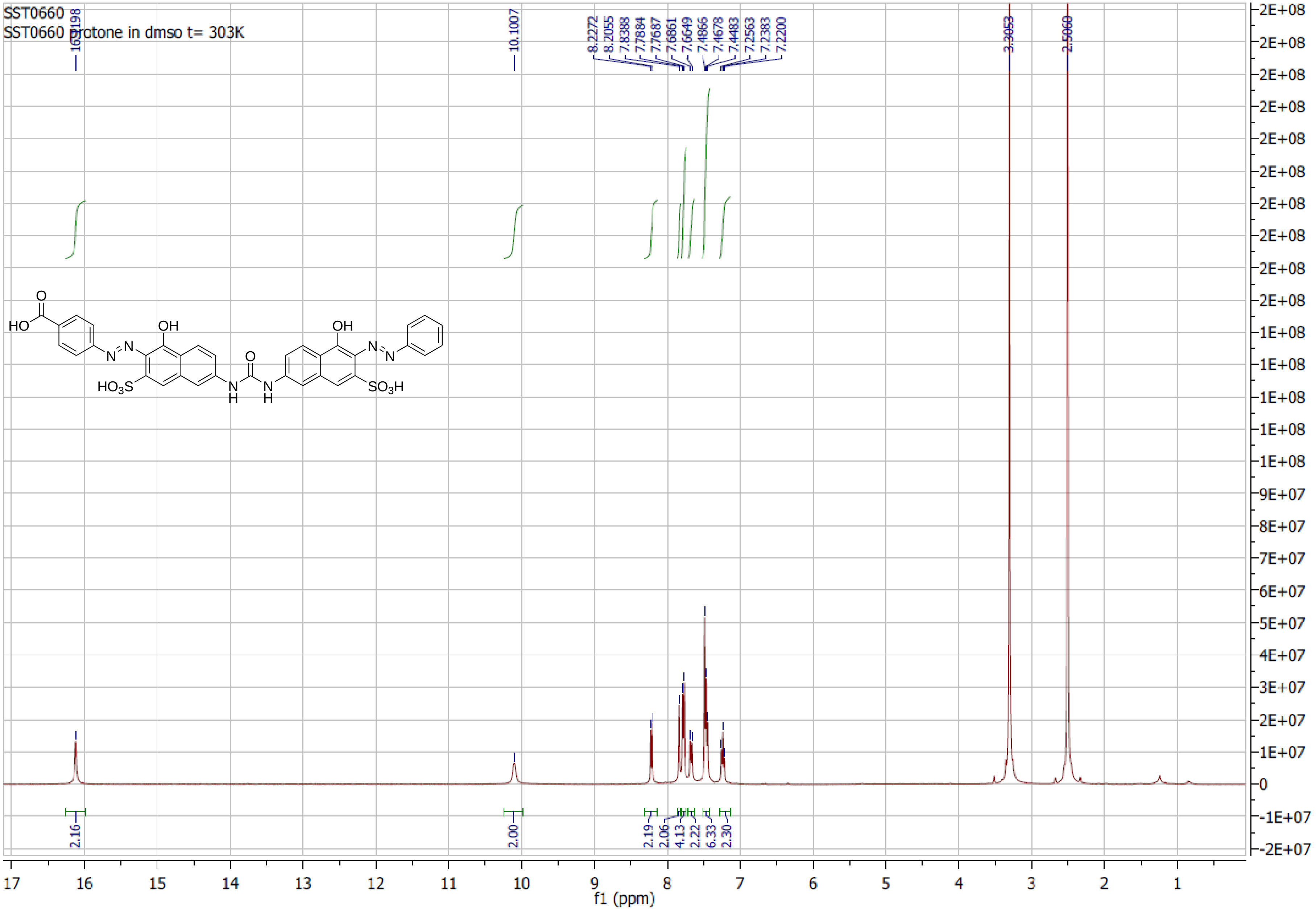
SST0660
SST0660 proton in dmso t= 303K
10.1007
8.2272
8.2055
7.8388
7.7884
7.7687
7.6861
7.6649
7.4866
7.4678
7.4483
7.2563
7.2383
7.2200
3.3053
2.5060
2.16
2.00
2.19
2.06
4.13
2.22
6.33
2.30
f1 (ppm)

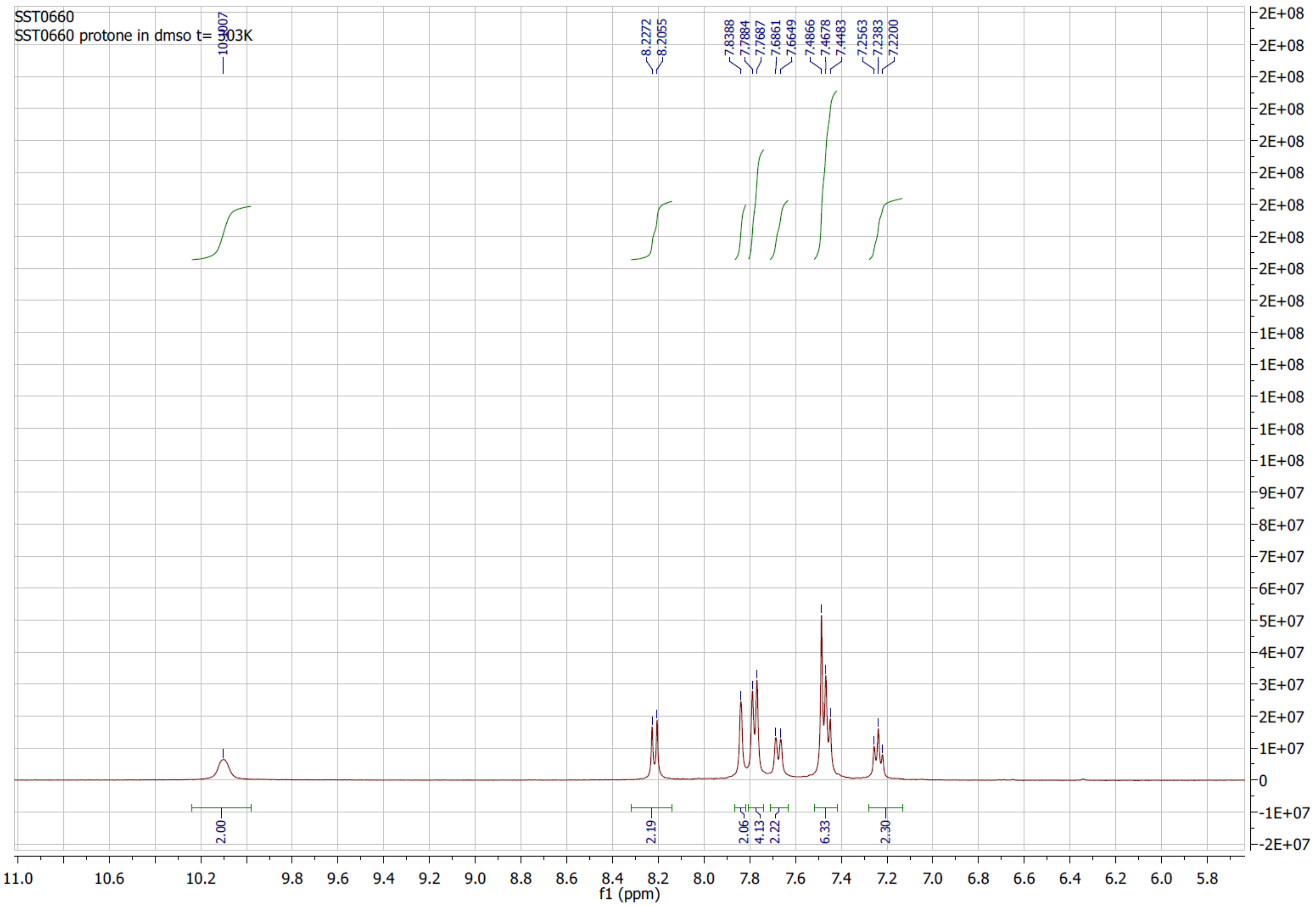
SST0660
SST0660 protone in dmso t= 303K
10.0907
8.2272
8.2055
7.8388
7.7884
7.7687
7.6861
7.6649
7.4866
7.4678
7.4483
7.2563
7.2383
7.2200
2.00
2.19
2.06
4.13
2.22
6.33
2.30
f1 (ppm)

Compound **20**

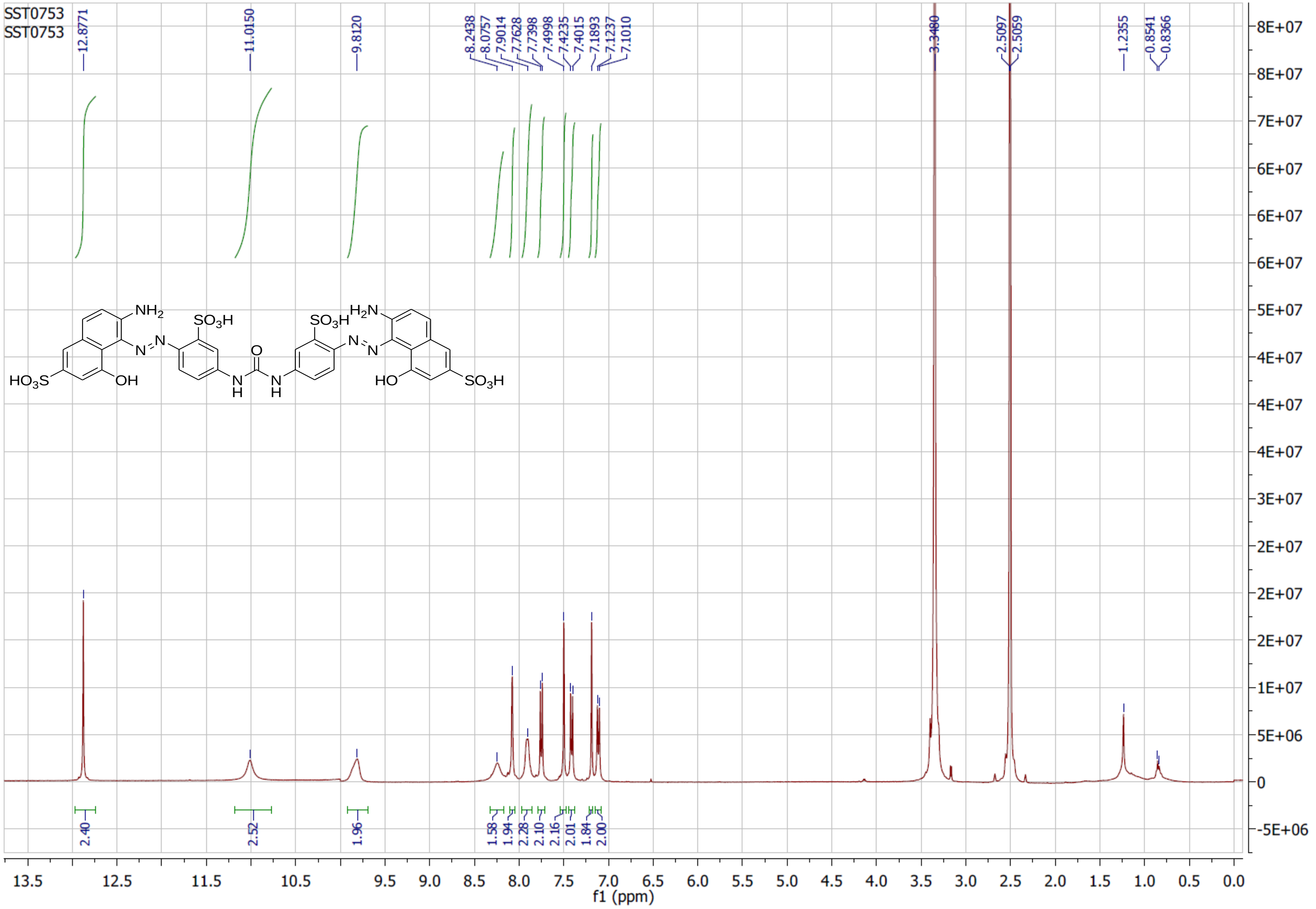
SST0753
SST0753
12.8771
11.0150
9.8120
8.2438
8.0757
7.9014
7.7628
7.7398
7.4998
7.4235
7.4015
7.1893
7.1237
7.1010
3.3480
2.5097
2.5059
1.2355
0.8541
0.8366
2.40
2.52
1.96
1.58
1.94
2.28
2.10
2.16
2.01
1.84
2.00
f1 (ppm)

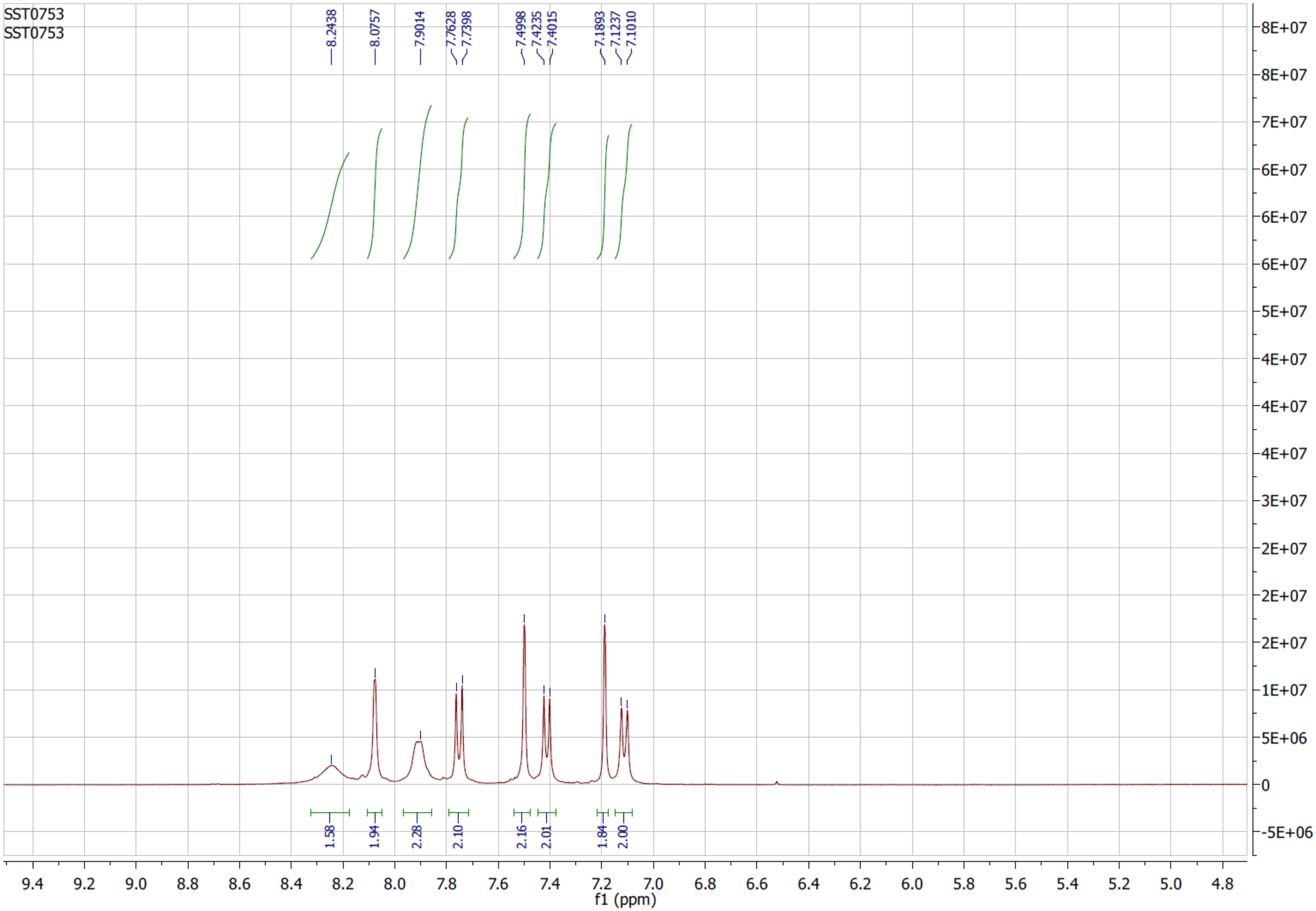
SST0753
SST0753
8.2438
8.0757
7.9014
7.7628
7.7398
7.4998
7.4235
7.4015
7.1893
7.1237
7.1010
1.58
1.94
2.28
2.10
2.16
2.01
1.84
2.00
f1 (ppm)

Compound **21**

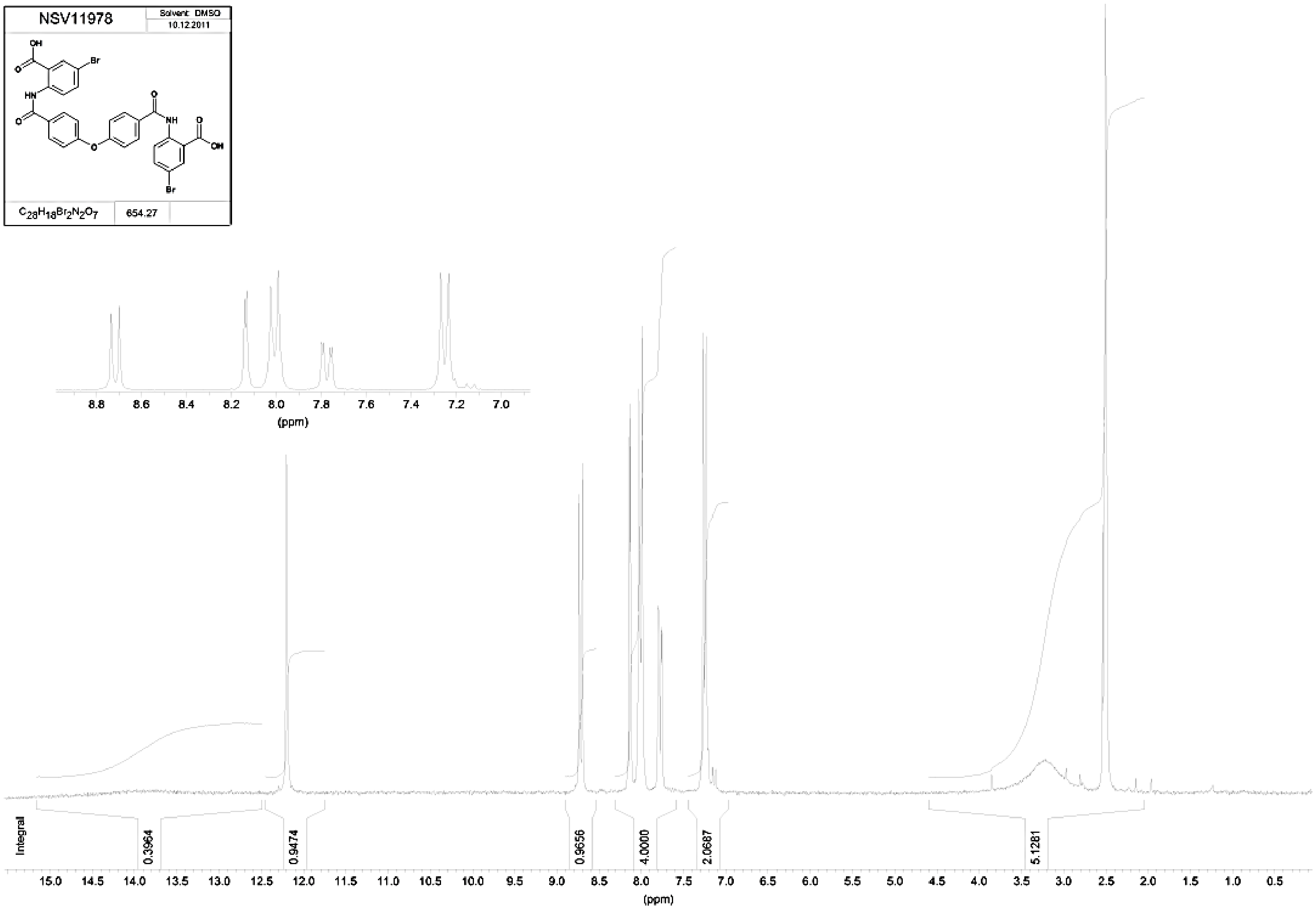
NSV11978
Solvent: DMSO
10.12.2011
$C_{28}H_{18}Br_2N_2O_7$
654.27
Integral
0.3964
0.9474
0.9656
4.0000
2.0687
5.1281
(ppm)

Compound **24**

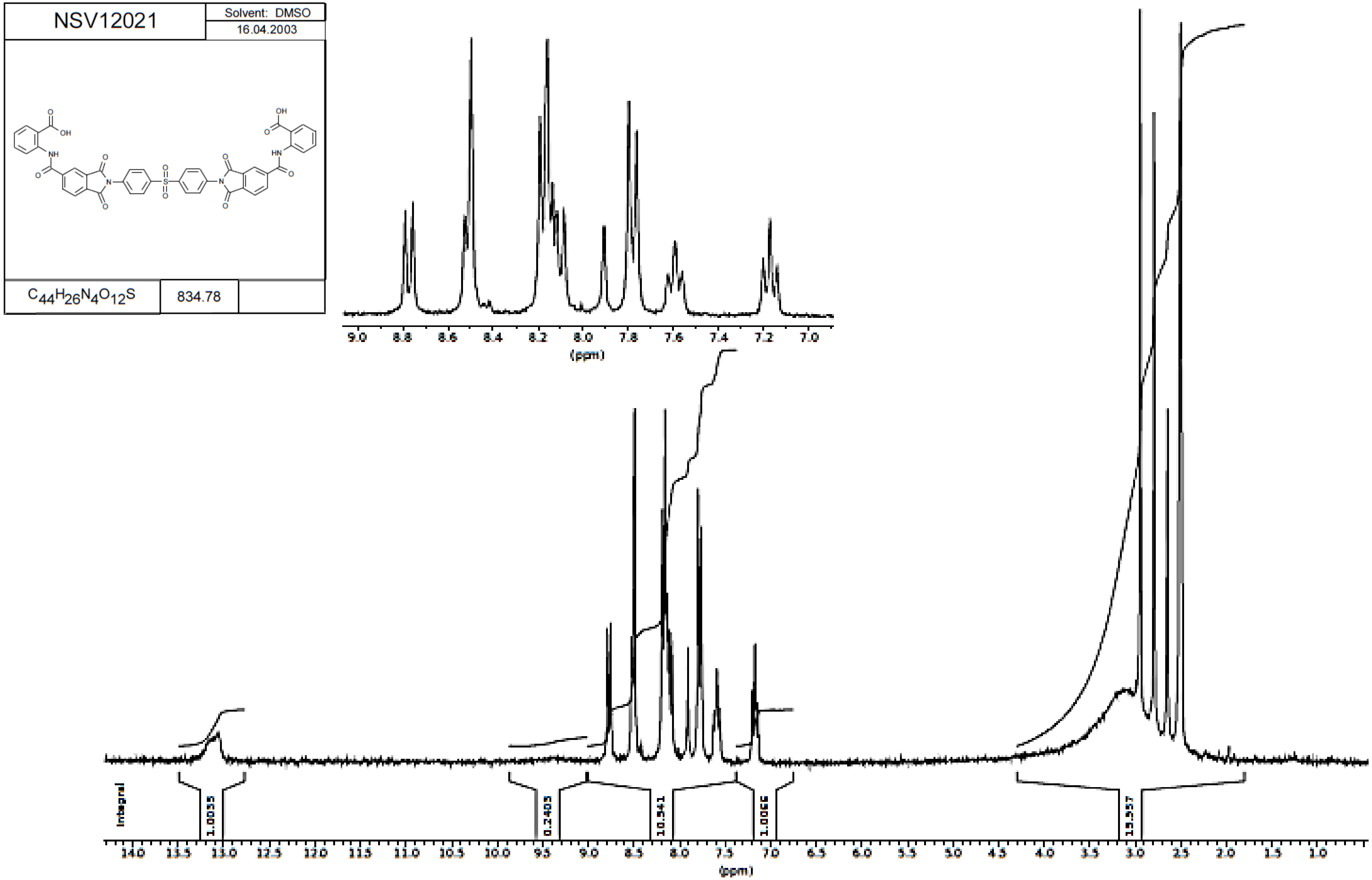

NSV12021
Solvent: DMSO
16.04.2003
C44H26N4O12S
834.78
(ppm)
Integral
1.0035
0.2405
10.541
1.0066
15.557
(ppm)

Compound **29**

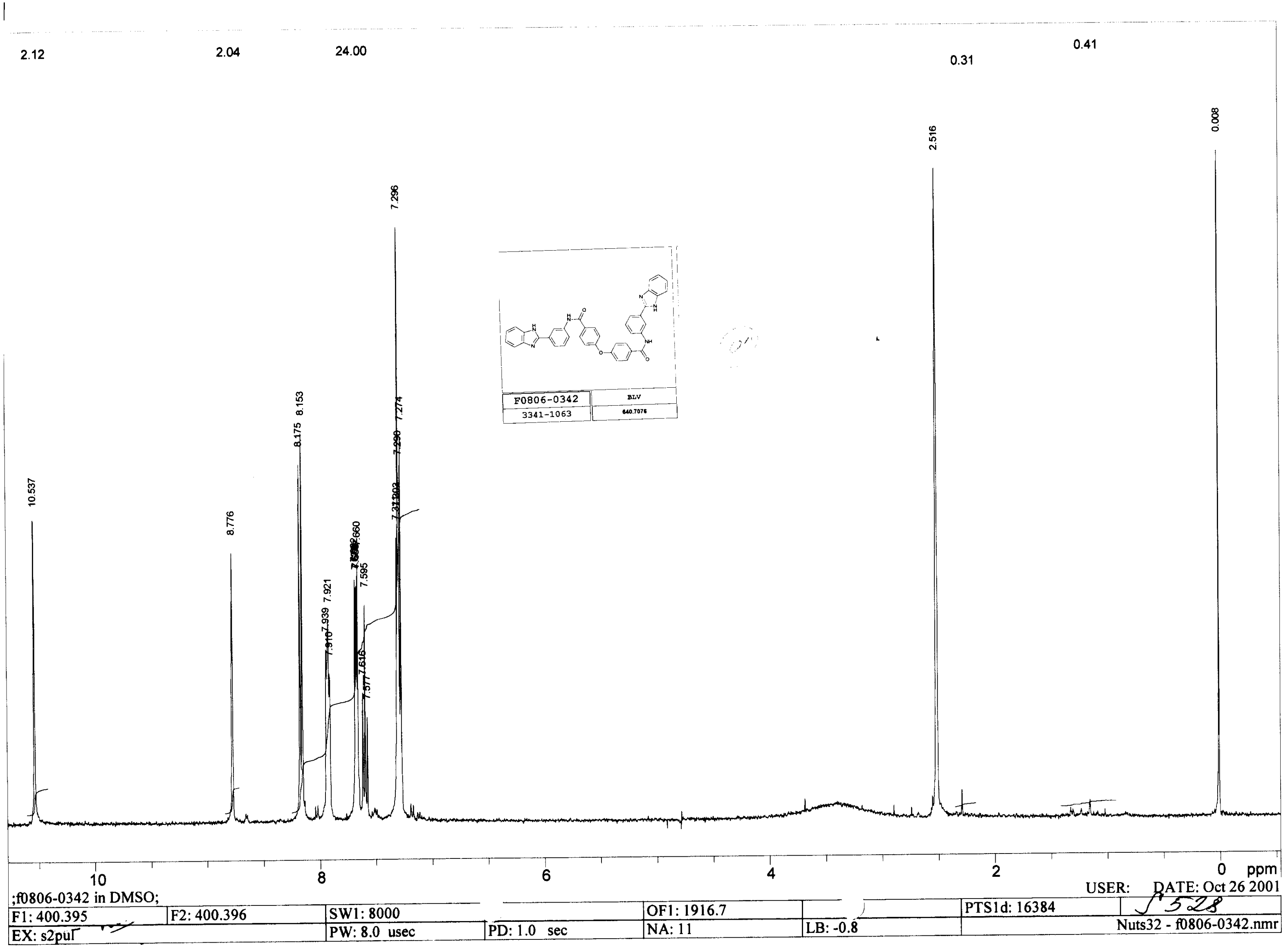

2.12
2.04
24.00
0.31
0.41
10.537
8.776
8.175 8.153
7.939 7.921
7.910
7.660
7.595
7.616
7.577
7.296
7.298 7.274
2.516
0.008
F0806-0342
BLV
3341-1063
640.7076
10
8
6
4
2
0 ppm
;f0806-0342 in DMSO;
USER:
DATE: Oct 26 2001
F1: 400.395
F2: 400.396
SW1: 8000
OF1: 1916.7
PTS1d: 16384
EX: s2pul
PW: 8.0 usec
PD: 1.0 sec
NA: 11
LB: -0.8
Nuts32 - f0806-0342.nmr

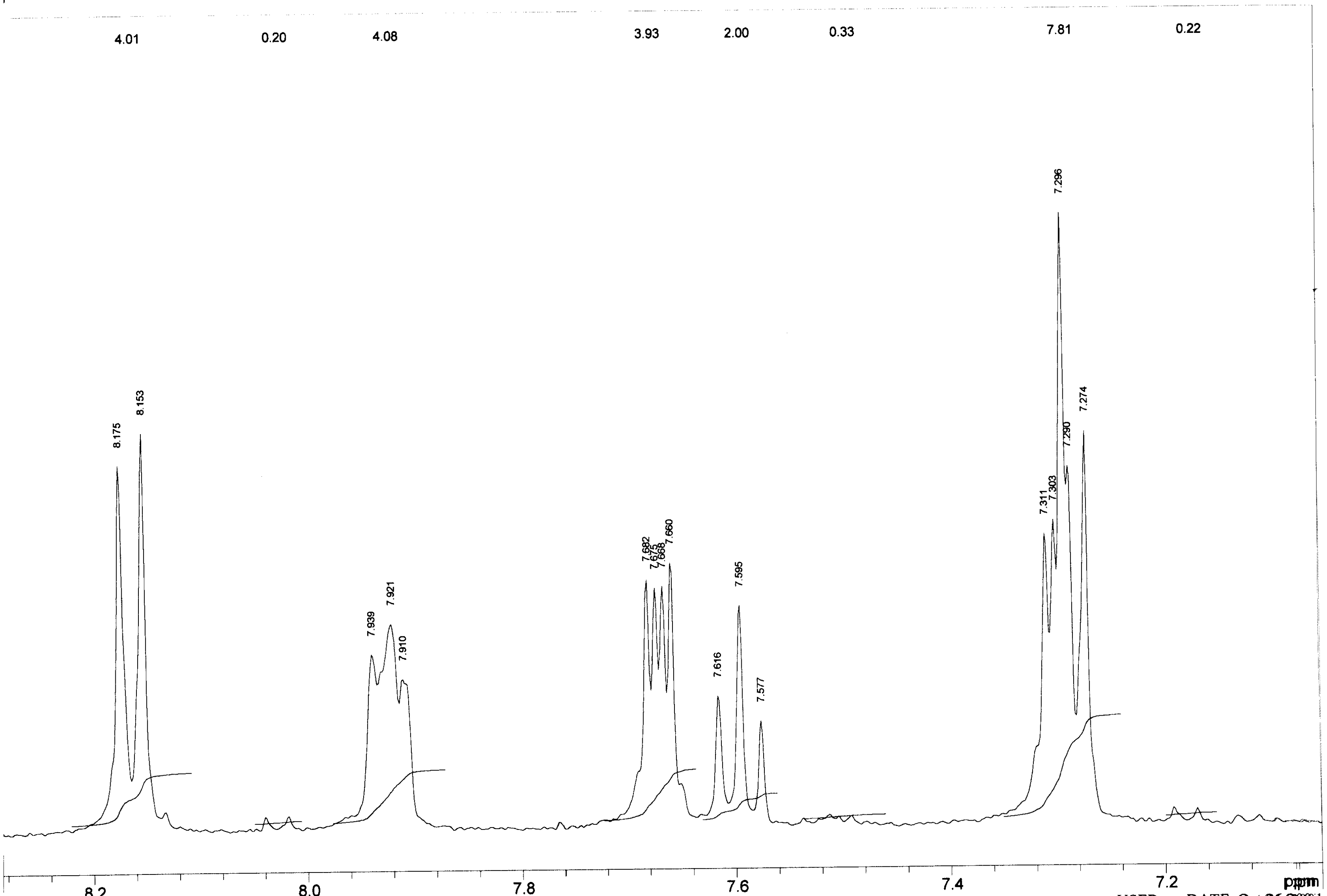

USER: DATE: Oct 26 2001

;f0806-0342 in DMSO;

F1: 400.395	F2: 400.396	SW1: 8000		OF1: 1916.7		PTS1d: 16384
EX: s2pul		PW: 8.0 usec	PD: 1.0 sec	NA: 11	LB: -0.8	Nuts32 - f0806-0342.mmr

Compound **34**

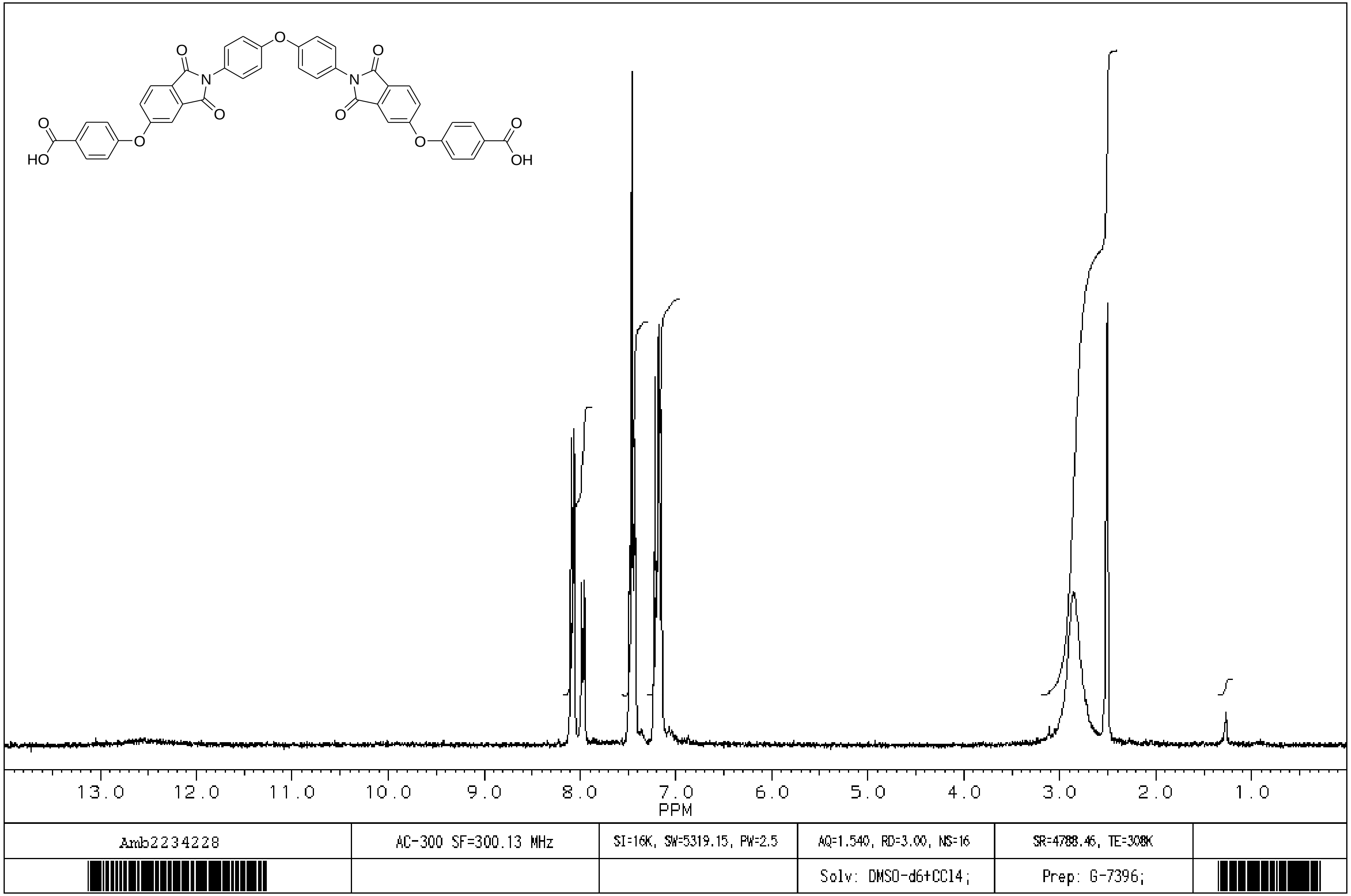
13.0
12.0
11.0
10.0
9.0
8.0
7.0
PPM
6.0
5.0
4.0
3.0
2.0
1.0
Amb2234228
AC-300 SF=300.13 MHz
SI=16K, SW=5319.15, PW=2.5
AQ=1.540, RD=3.00, NS=16
SR=4788.46, TE=308K
Solv: DMSO-d6+CCl4;
Prep: G-7396;

Compound **35**

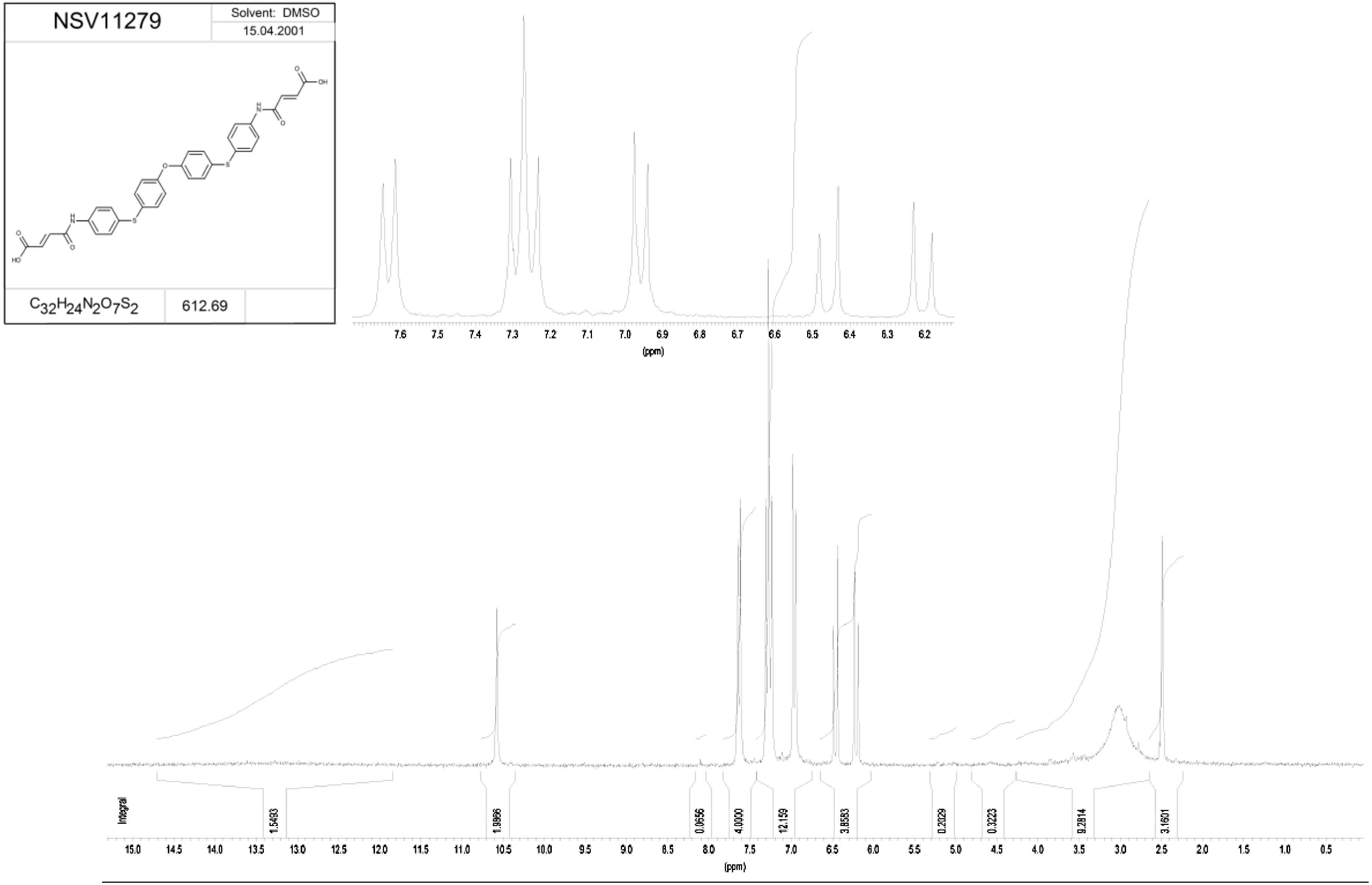
NSV11279
Solvent: DMSO
15.04.2001
C32H24N2O7S2
612.69
(ppm)
Integral
1.5493
1.9866
0.0656
4.0000
12.159
3.8583
0.2029
0.3223
9.2814
3.1601
(ppm)

Compound **36**

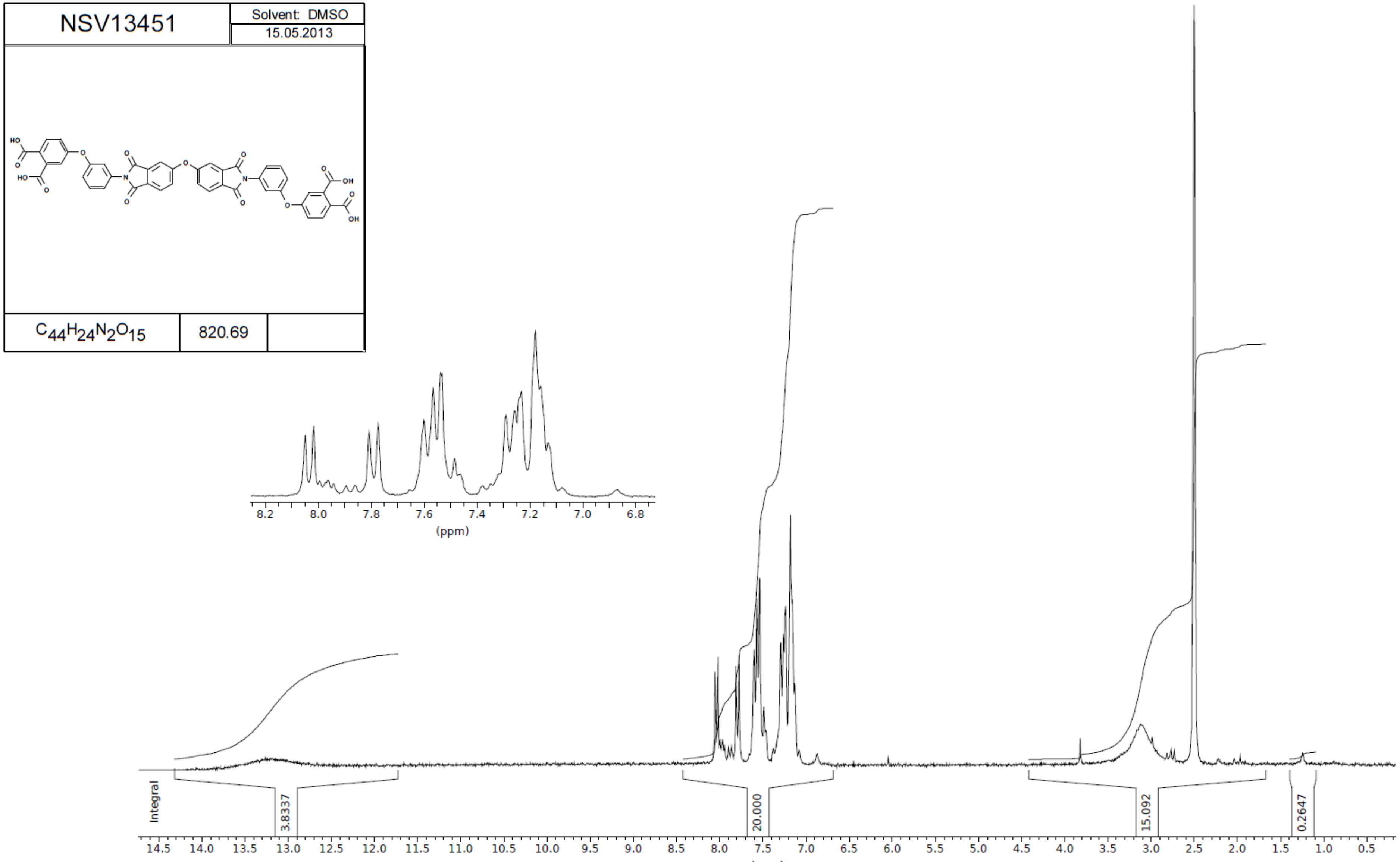

NSV13451
Solvent: DMSO
15.05.2013
C44H24N2O15
820.69
(ppm)
Integral
3.8337
20.000
15.092
0.2647

Compound **48**

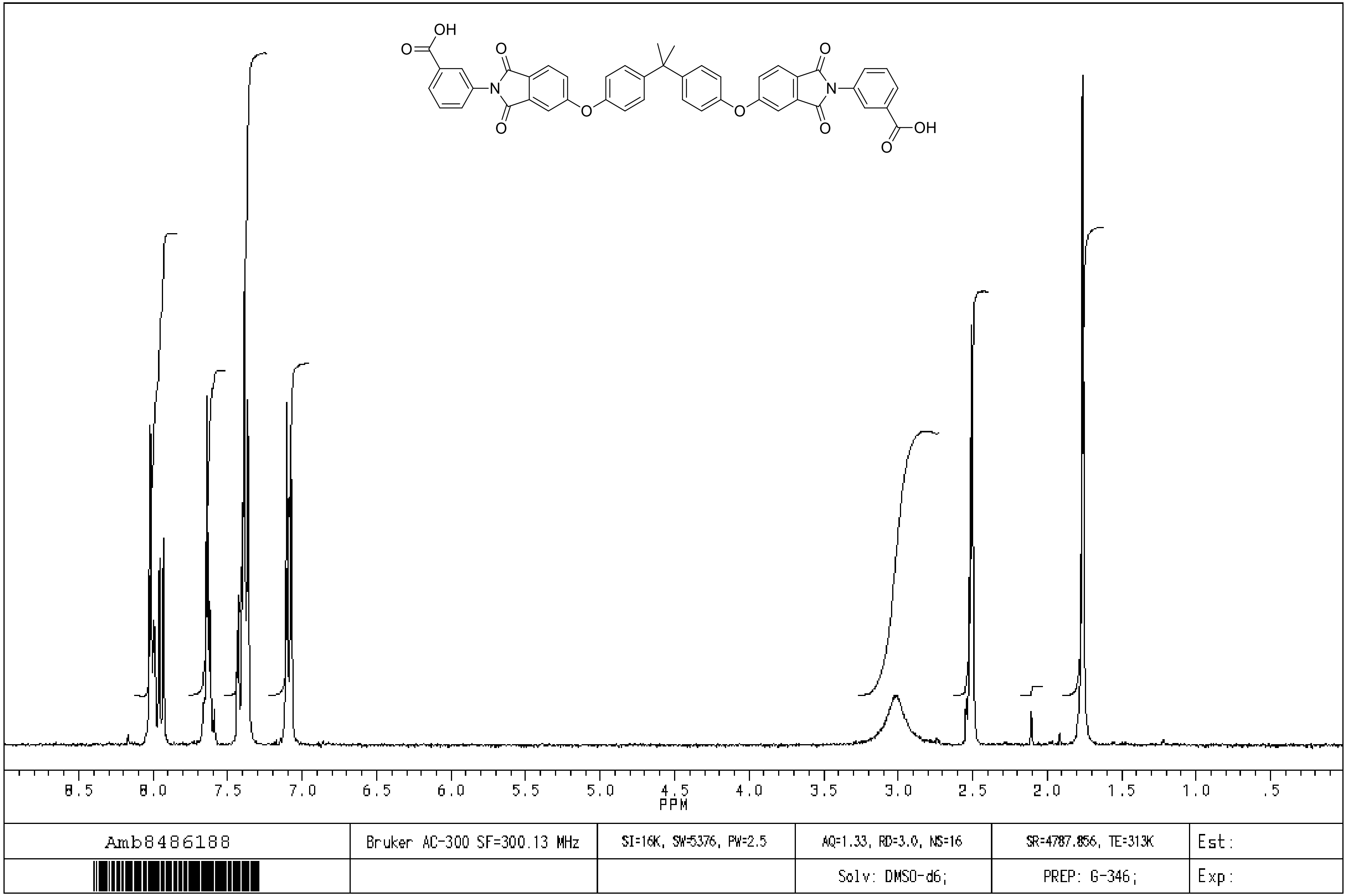
OH
O
N
O
O
O
N
O
OH
8.5 8.0 7.5 7.0 6.5 6.0 5.5 5.0 4.5 4.0 3.5 3.0 2.5 2.0 1.5 1.0 .5
PPM
Amb8486188
Bruker AC-300 SF=300.13 MHz
SI=16K, SW=5376, PW=2.5
AQ=1.33, RD=3.0, NS=16
SR=4787.856, TE=313K
Est:
Solv: DMSO-d6;
PREP: G-346;
Exp:

Compound **49**

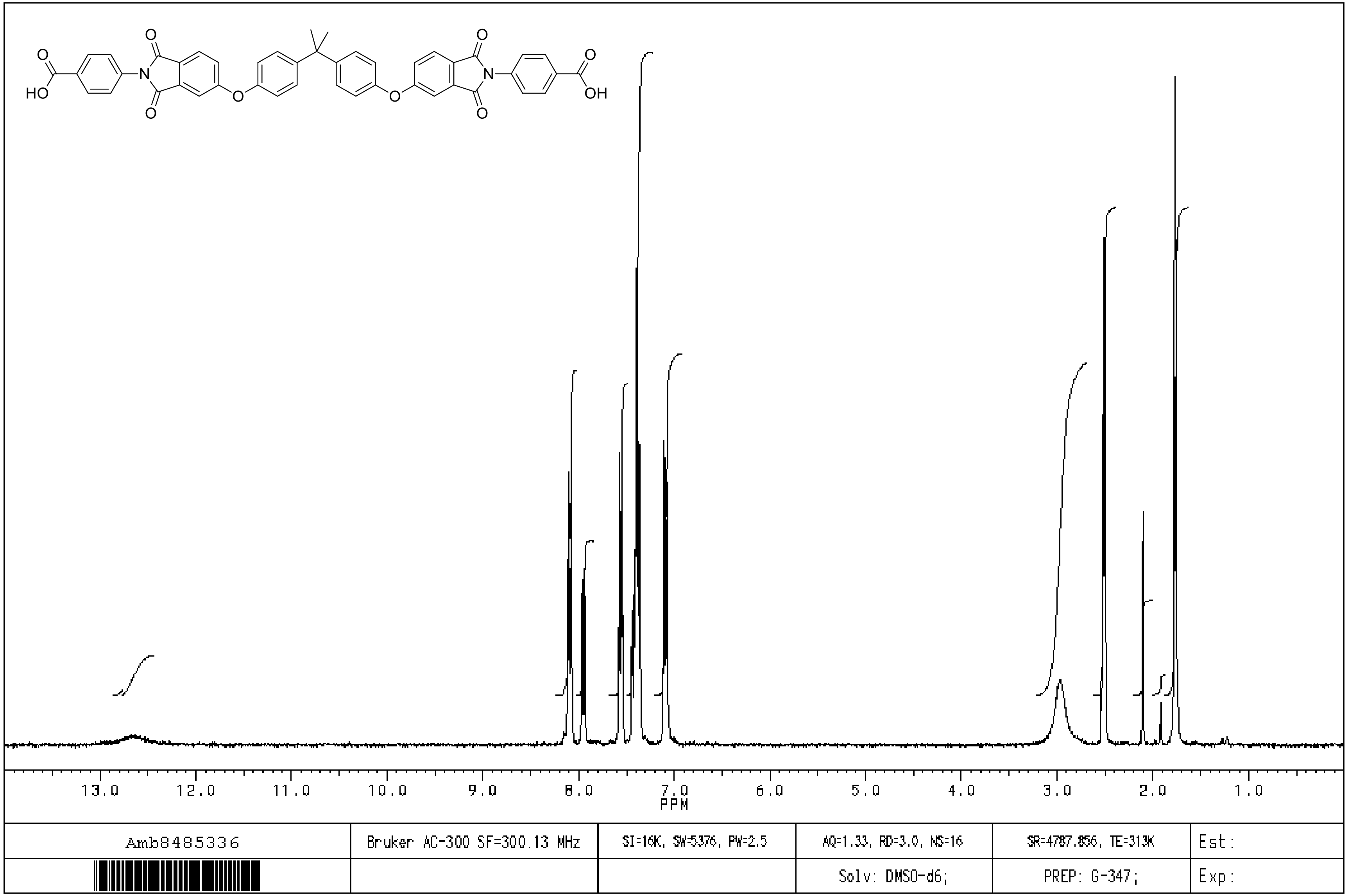
HO
O
N
O
O
O
O
N
O
O
OH
13.0
12.0
11.0
10.0
9.0
8.0
7.0
PPM
6.0
5.0
4.0
3.0
2.0
1.0
Amb8485336
Bruker AC-300 SF=300.13 MHz
SI=16K, SW=5376, PW=2.5
AQ=1.33, RD=3.0, NS=16
SR=4787.856, TE=313K
Est:
Solv: DMSO-d6;
PREP: G-347;
Exp:

Compound **50**

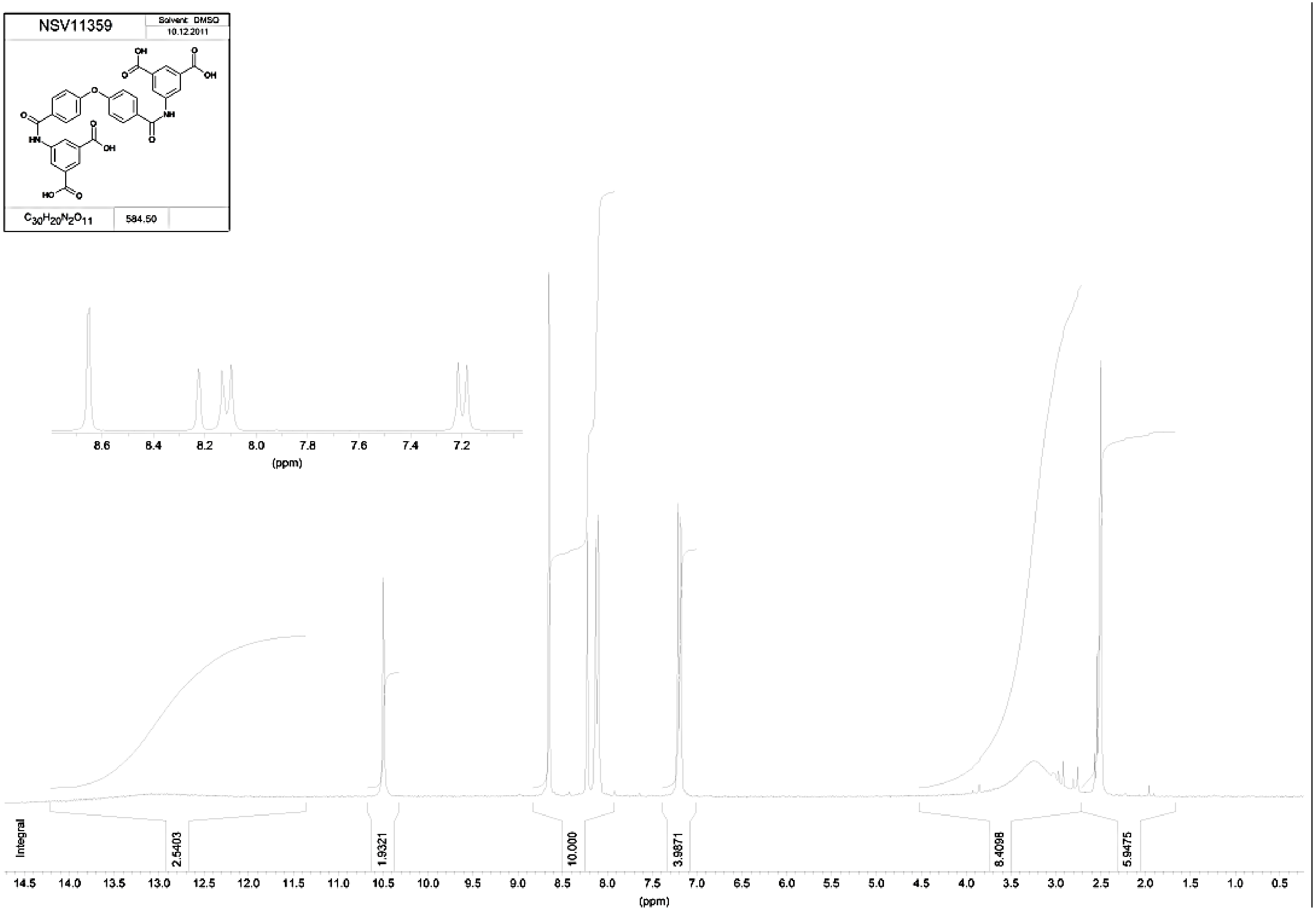

g

Compound **57**

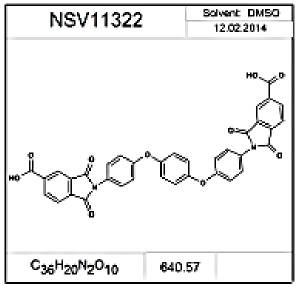

NSV11322
Solvent: DMSO
12.02.2014
$C_{36}H_{20}N_2O_{10}$
640.57

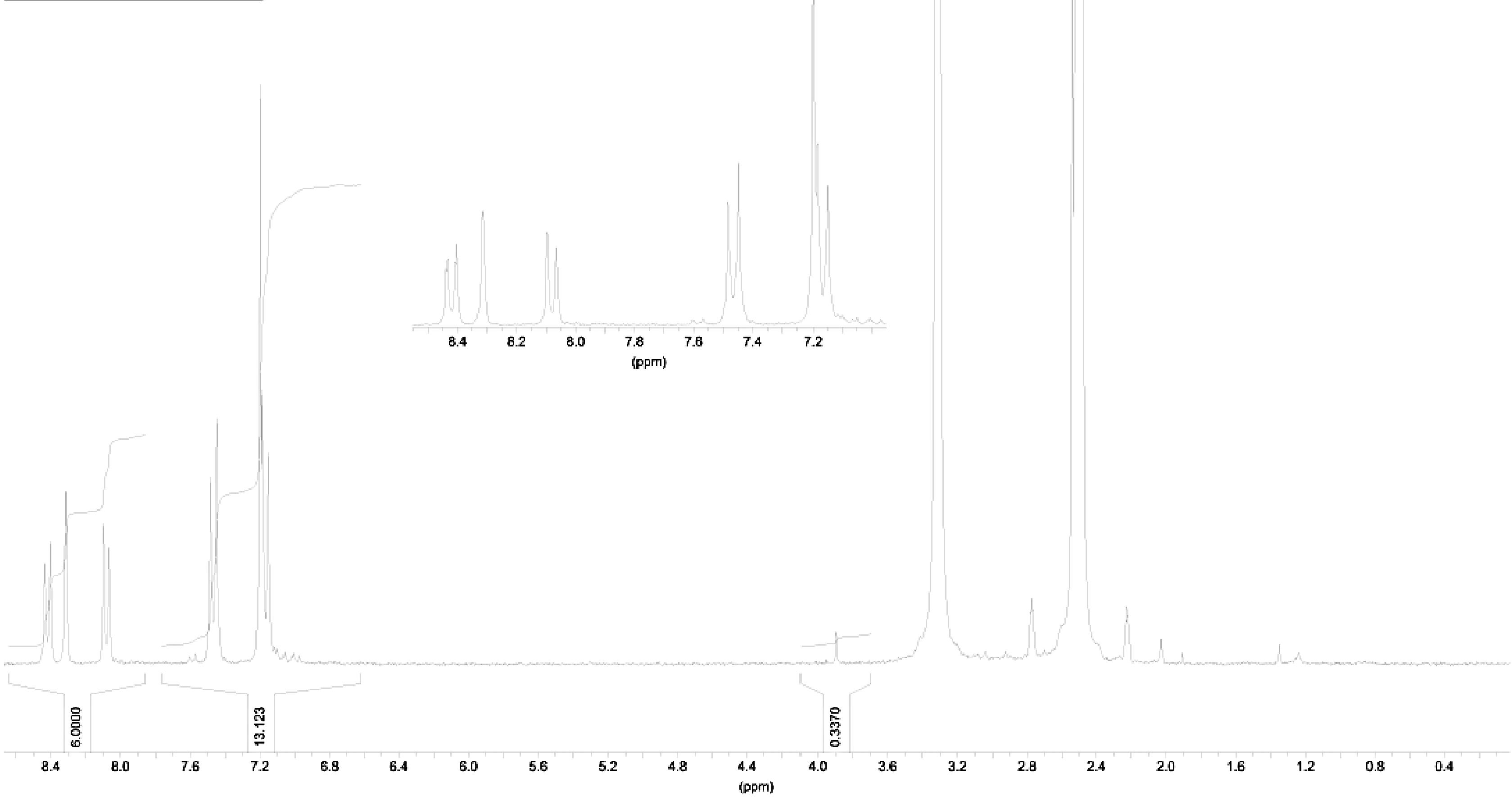

8.4
8.2
8.0
7.8
7.6
7.4
7.2
(ppm)
6.0000
13.123
0.3370
8.4
8.0
7.6
7.2
6.8
6.4
6.0
5.6
5.2
4.8
4.4
4.0
3.6
3.2
2.8
2.4
2.0
1.6
1.2
0.8
0.4
(ppm)

Compound **58**

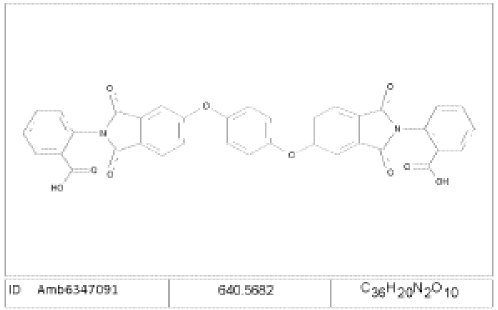

ID Amb6347091
640.5682
$C_{36}H_{20}N_2O_{10}$

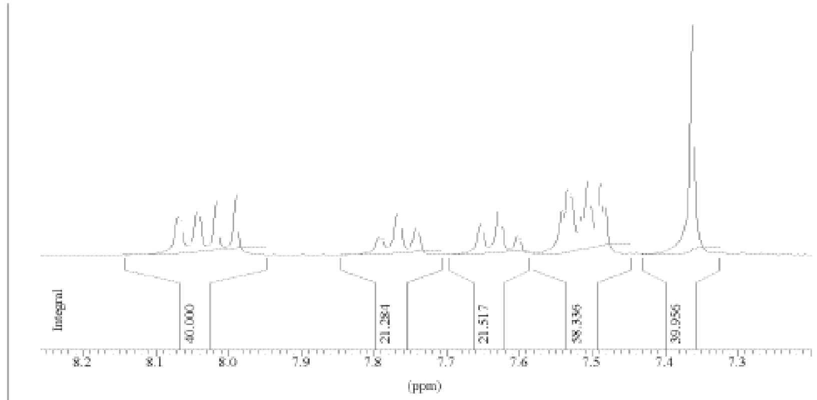

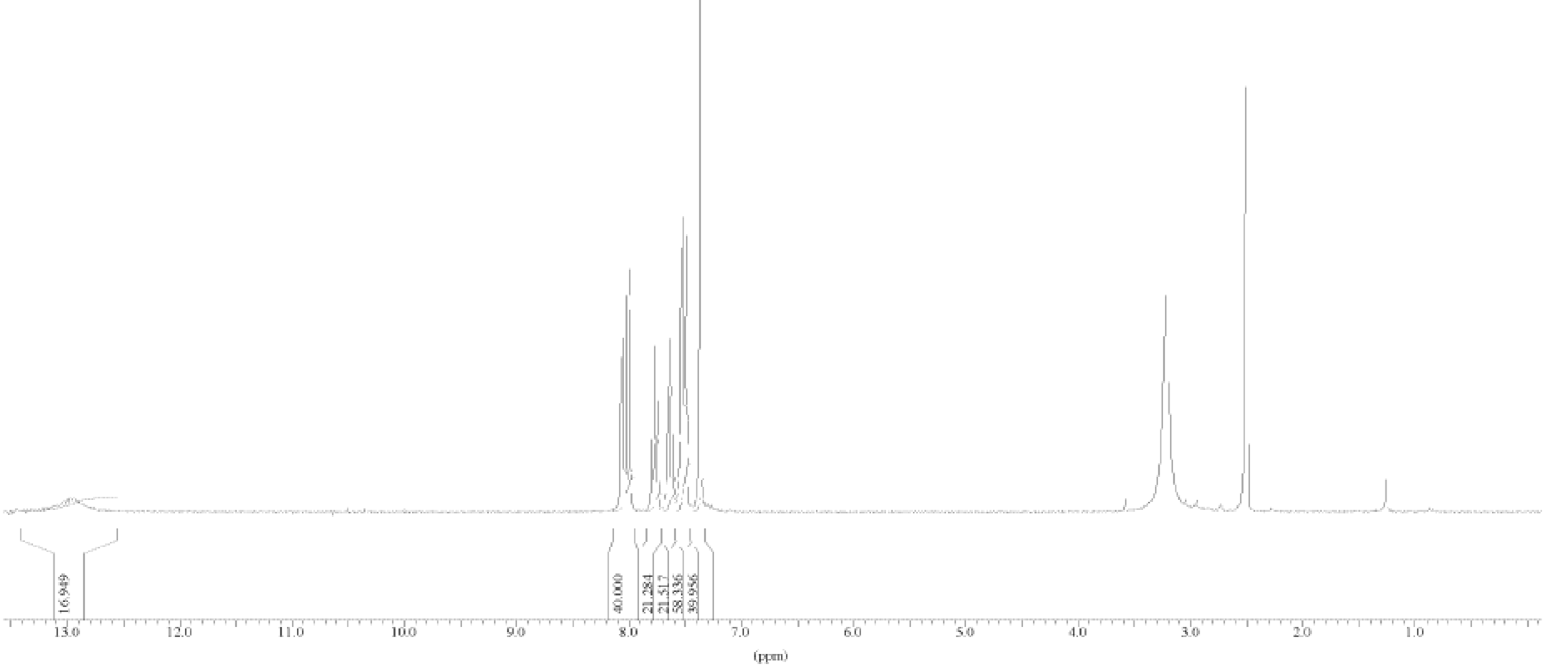

Integral
40.000
21.284
21.517
58.336
39.956
(ppm)
16.949

Compound **59**

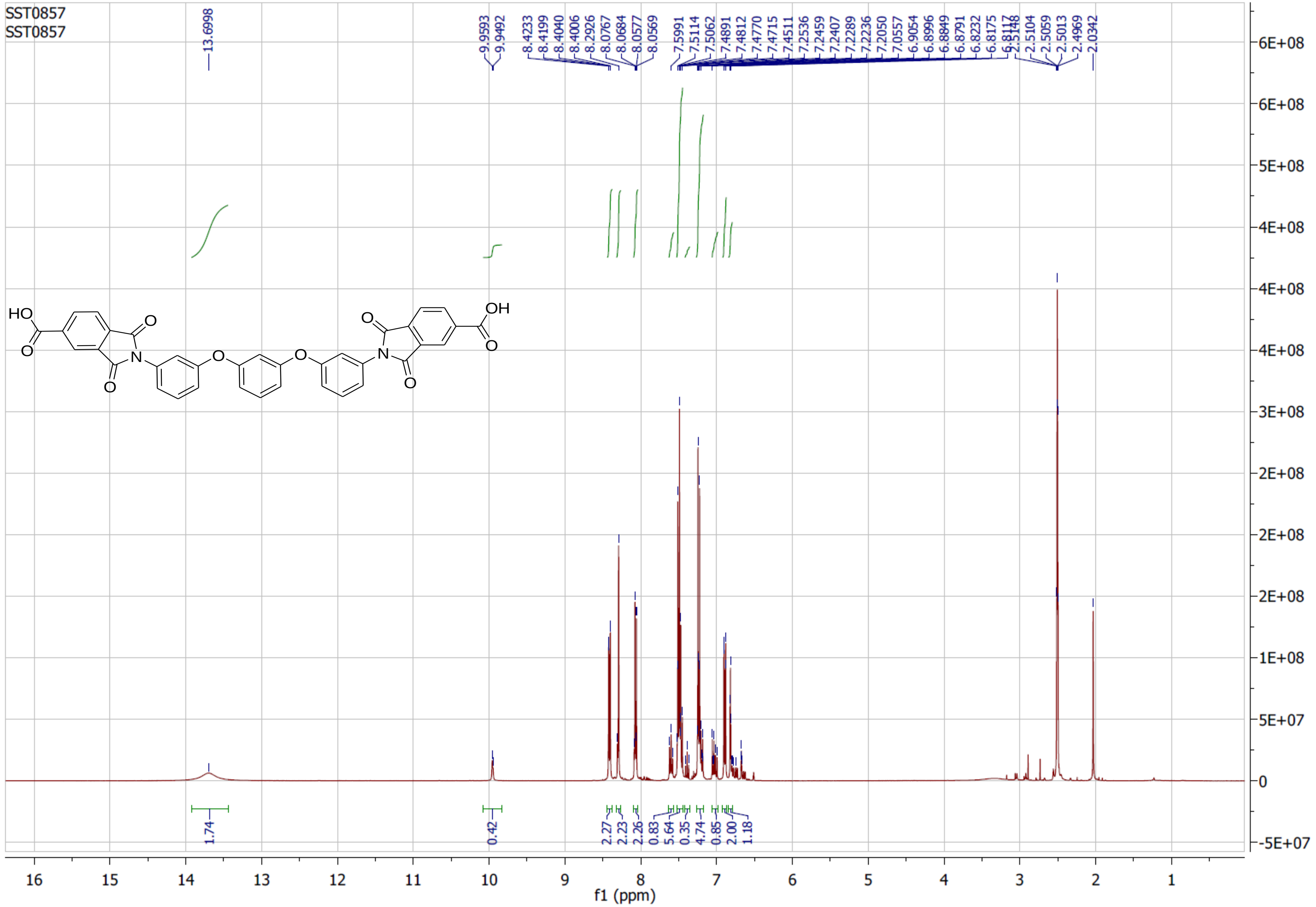

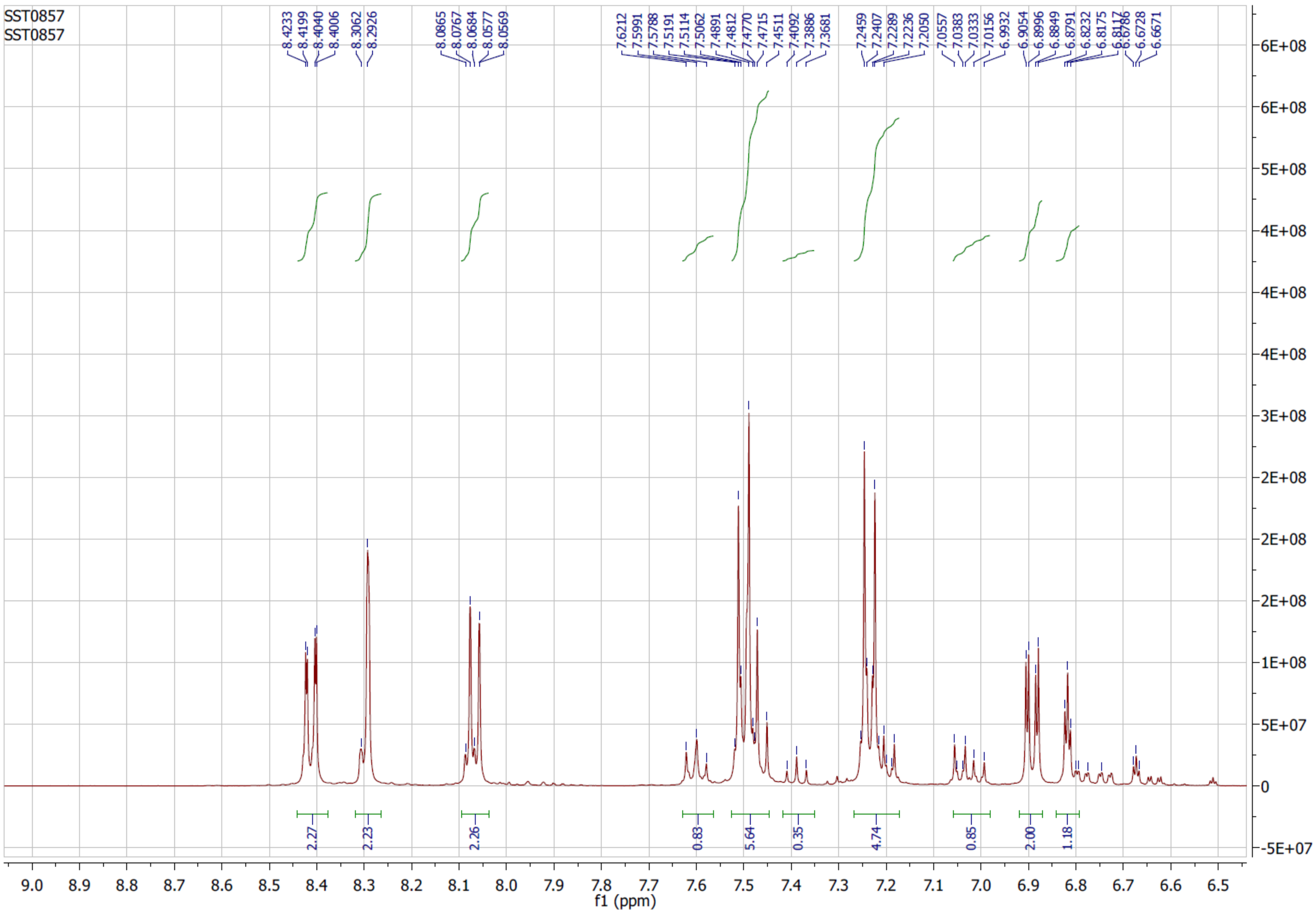
SST0857
SST0857
8.4233
8.4199
8.4040
8.4006
8.3062
8.2926
8.0865
8.0767
8.0684
8.0577
8.0569
7.6212
7.5991
7.5788
7.5191
7.5114
7.5062
7.4891
7.4812
7.4770
7.4715
7.4511
7.4092
7.3886
7.3681
7.2459
7.2407
7.2289
7.2236
7.2050
7.0557
7.0383
7.0333
7.0156
6.9932
6.9054
6.8996
6.8849
6.8791
6.8232
6.8175
6.8117
6.6786
6.6728
6.6671
2.27
2.23
2.26
0.83
5.64
0.35
4.74
0.85
2.00
1.18
f1 (ppm)

Compound **61**

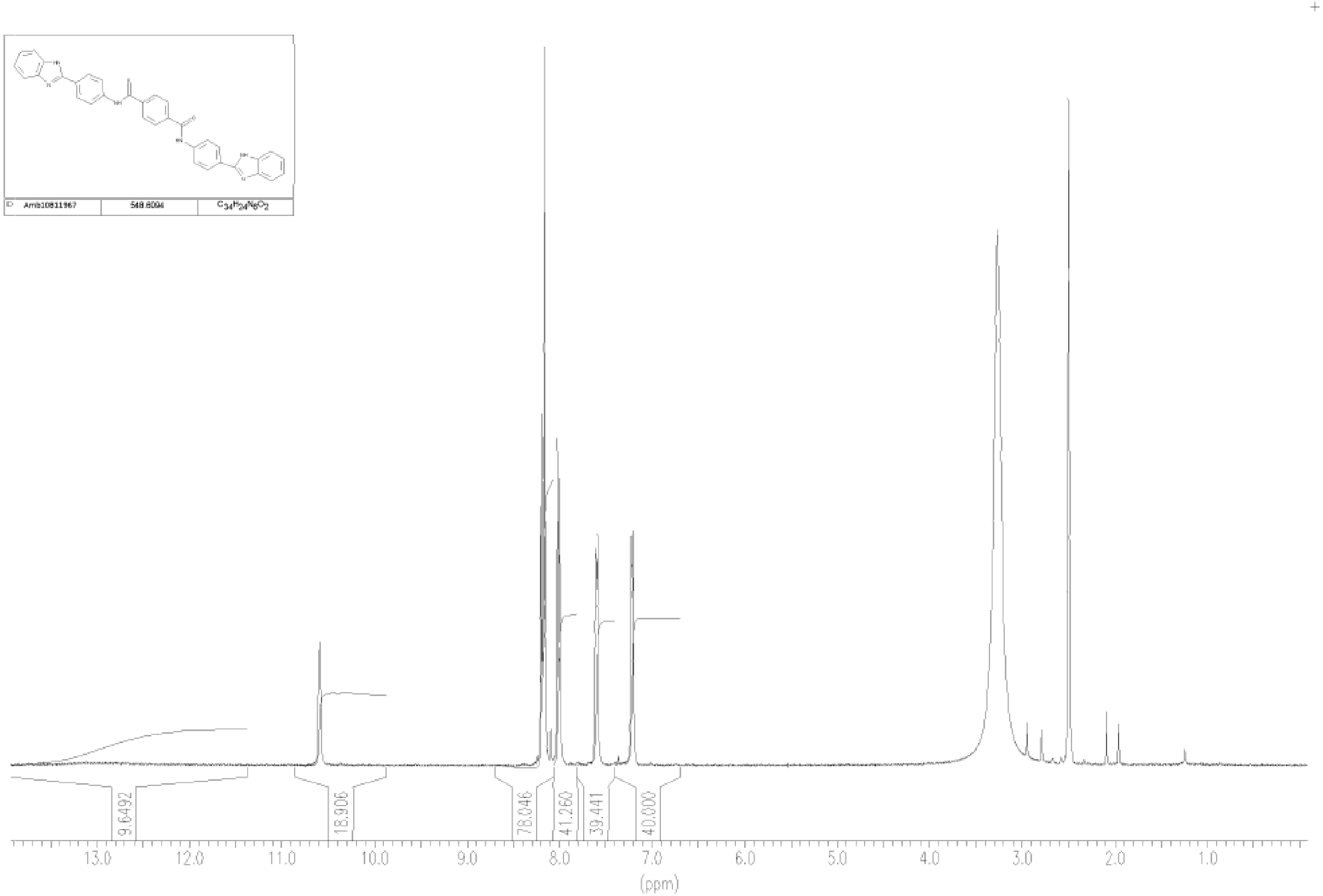

Amb10811967
548.6094
$C_{34}H_{24}N_6O_2$
9.6492
18.906
78.046
41.260
39.441
40.000
13.0
12.0
11.0
10.0
9.0
8.0
7.0
6.0
5.0
4.0
3.0
2.0
1.0
(ppm)

Compound **62**

NSV11852 | 1H NMR | Solvent: DMSO |17.07.2017

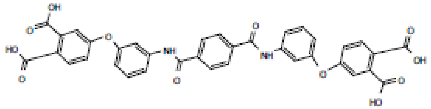

MW: 676.582
MF: $C_{36}H_{24}N_2O_{12}$

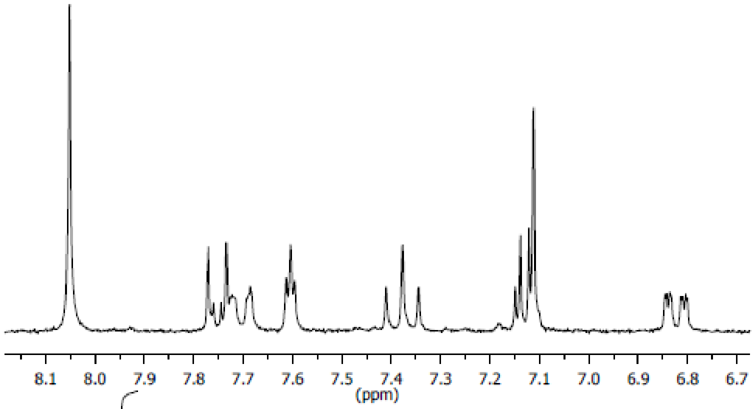

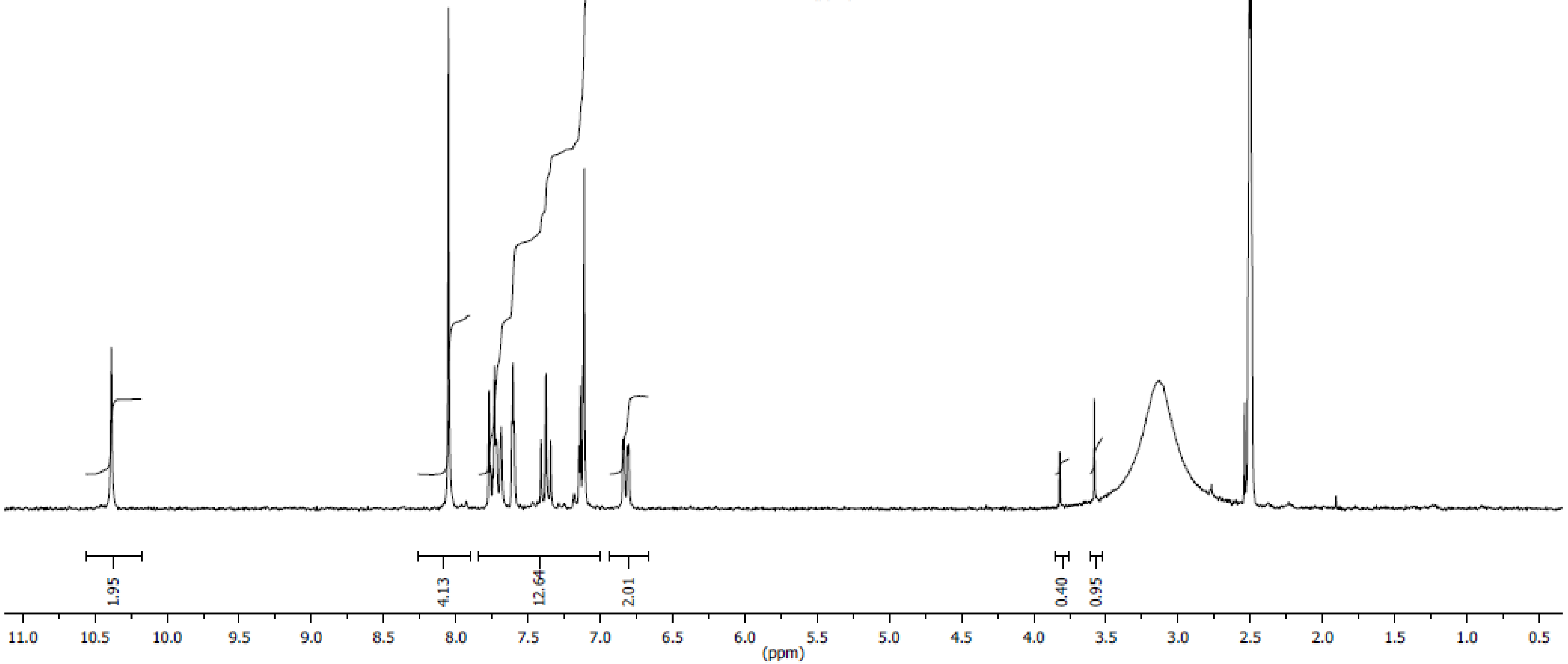

Compound **63**

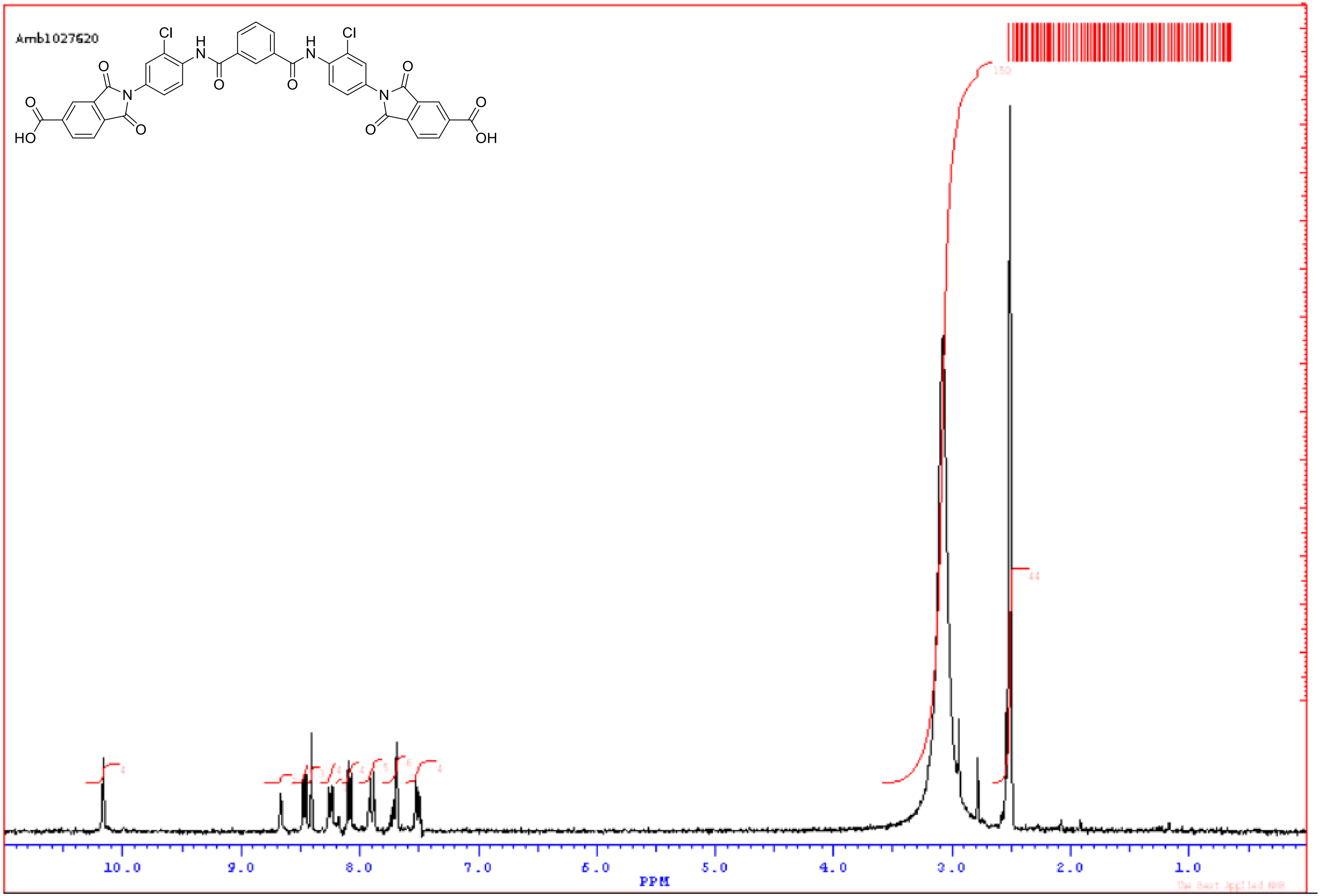

Supplement: Supplemental Material [file IENZ_A_1811701_SM0304.pdf]
